# Thoroughly Calibrated Modular Agent-Based Model of the Human Cardiovascular and Renal Systems for Blood Pressure Regulation in Health and Disease

**DOI:** 10.3389/fphys.2021.746300

**Published:** 2021-11-11

**Authors:** Elena Kutumova, Ilya Kiselev, Ruslan Sharipov, Galina Lifshits, Fedor Kolpakov

**Affiliations:** ^1^Department of Computational Biology, Sirius University of Science and Technology, Sochi, Russia; ^2^Laboratory of Bioinformatics, Federal Research Center for Information and Computational Technologies, Novosibirsk, Russia; ^3^Biosoft.Ru, Ltd., Novosibirsk, Russia; ^4^Specialized Educational Scientific Center, Novosibirsk State University, Novosibirsk, Russia; ^5^Laboratory for Personalized Medicine, Center of New Medical Technologies, Institute of Chemical Biology and Fundamental Medicine SB RAS, Novosibirsk, Russia

**Keywords:** mathematical modeling, agent-based model, modular model, cardiovascular system, renal system, blood pressure regulation

## Abstract

Here we present a modular agent-based mathematical model of the human cardiovascular and renal systems. It integrates the previous models primarily developed by A. C. Guyton, F. Karaaslan, K. M. Hallow, and Y. V. Solodyannikov. We performed the model calibration to find an equilibrium state within the normal vital sign ranges for a healthy adult. We verified the model’s abilities to reproduce equilibrium states with abnormal physiological values related to different combinations of cardiovascular diseases (such as systemic hypertension, chronic heart failure, pulmonary hypertension, etc.). For the model creation and validation, we involved over 200 scientific studies covering known models of the human cardiovascular and renal functions, biosimulation platforms, and clinical measurements of physiological quantities in normal and pathological conditions. We compiled detailed documentation describing all equations, parameters and variables of the model with justification of all formulas and values. The model is implemented in BioUML and available in the web-version of the software.

## Introduction

Mathematical models provide tools for understanding of human physiology via integration and analysis of biological data from multiple ranges and time scales under normal and pathological conditions. A number of such models have already been used to investigate different aspects of individual systems and processes of the human body (Guyton et al., 1972; Ikeda et al., 1979; Uttamsingh et al., 1985; Ottesen et al., 2004; Karaaslan et al., 2005, 2014; Proshin and Solodyannikov, 2006; Abram et al., 2007; Thomas et al., 2007; Hester et al., 2011; Paeme et al., 2011; Hallow et al., 2014, 2021; Hallow and Gebremichael, 2017a; Rosalina et al., 2019). Most of them originate from the control-theory block model of circulatory regulation proposed by Guyton et al. (1972). This model was implemented in different programming languages (Moss et al., 2012) and reused in further whole-body models (Abram et al., 2007; Hester et al., 2011). Guyton et al. (1972) established the foundation for our understanding of the relations between long-term blood pressure level and sodium and water balance. And in this sense, their model is innovative and revolutionary (Montani and Vliet, 2009; Beard, 2018). However, its application for research purposes is difficult due to the model complexity, the lack of a complete detailed description, physiological limitations for some parameters, and some misconceptions (Montani and Vliet, 2009).

To solve narrowly focused problems, more compact models have been created recently. Thus, Karaaslan et al. (2005) explored the renal sympathetic nerve activity related mechanisms affecting kidney functions and causing increase of arterial pressure in hypertension. Hallow et al. (2014) simulated pharmacodynamic effects of the antihypertensive therapy, and then created a detailed model of renal physiology (Hallow and Gebremichael, 2017a) applied for investigation of salt-sensitive hypertension (Hallow and Gebremichael, 2017b). A number of models focused on cardiovascular hemodynamics (Proshin and Solodyannikov, 2006; Thomas et al., 2007; Paeme et al., 2011; Rosalina et al., 2019).

Accumulation of the particular models of human physiology leads to the next logical step: “gluing” of these models (Kassab and Guccione, 2019). However, this step results in the creation of complex entities. The only way to deal with such complexity is dividing the biological systems into subsystems, and the corresponding models into modules. This approach simplifies the modeling process and, at the same time, allows you to gradually improve the model by replacement and addition of modules. Another problem that arises in the model integration is related to the combination of processes acting at significantly different time scales. For example, a single heartbeat takes less than a second, whereas a renal function is associated with the long-term regulation of the salt and water balance. Merging the equations for these processes gives a very stiff problem which in the specified case can be solved by application of an agent-based approach.

At the present time, there are several biosimulation platforms designed for modeling and analysis of the human physiological processes. For instance, the Entelos PhysioLab platforms are implemented for drug development and have been applied to projects in diabetes, rheumatoid arthritis, asthma, and skin sensitization (Rullmann et al., 2005; Maxwell and Mackay, 2008; Klinke, 2015). These projects are widely used by pharmaceutical companies (Pfizer, Novartis, etc.) in preclinical trials of new drugs, and capture the modeled physiology using a modular approach, assuming that smaller scale models are defined based on isolated components (cells or signaling pathways) connected together to reflect higher (intracellular) level behavior (Klinke, 2015). Another major project is the open-source, full-body human physiology engine BioGears (McDaniel et al., 2019). Its purpose is to provide realistic and comprehensive simulations for medical research and education. BioGears may be used as a standalone application or integrated with simulators, sensor interfaces, and other existing models. According to information from the developers, this platform operates with lumped parameters and, therefore, is not aimed at creating personalized models, but uses “average” person for analysis. One more environment for modeling and simulation of integrative human physiology is the HumMod software (Hester et al., 2011). It describes cardiovascular, respiratory, renal, neural, endocrine, skeletal muscle, and metabolic systems, and is constructed from empirical data obtained by authors from scientific literature. As in the case of BioGears, the user of HumMod defines a number of patient basic parameters (gender, height, weight, etc.) and operates with an average patient in the normal or pathological state. As an example of the environment for modeling of the circulatory system, we want to single out the Samara-Dialog platform designed on the basis of the cardiovascular hemodynamics model proposed by Proshin and Solodyannikov (2006)^1^. This environment is primarily intended to study the status of an athlete in the training process, but can also be used to simulate a wide range of the human cardiovascular system pathologies, including heart arrhythmias, ventricular dysfunctions, valvular failure, hypertension, etc.

All of the mentioned models and simulation platforms were created to investigate mechanisms of different cardiovascular and renal diseases in several abstract conditions, where parameter values are fixed on the basis of some average normal or pathological evaluations. However, cardiac models should account for the fact that humans vary (Wiśniowska et al., 2017). If we consider, in addition, the mathematical modeling to predict real-world effectiveness of drug interventions, we find that the most important limitation is the lack of external validation applying other data than those used for developing the models. In their review, Panayidou et al. (2016) concluded that such modeling is not widely used at present and not well validated.

Thus, in this work, we focus not only on creating a modular agent-based model of the cardiovascular and renal systems, but also perform detailed validation of parameters in order to conform to physiological ranges and reproduce equilibrium states corresponding to various combinations of cardiovascular diseases in real patients. This can later be used for personalized modeling and individual predictions on a case-by-case basis. For operation with the model, we used the BioUML software, which provided all the necessary tools for our research, and which our team has been developing since 2002 (Kolpakov et al., 2019).

## Materials and Methods

### Mathematical Formulation

The model contains a system of ordinary differential equations (ODEs):


(1) $$\begin{array}{c} \left\{ \begin{array}{c} \frac{\mathrm{dX}}{\mathrm{dt}}=\text{F}\left(X,Y,P,t\right),\\ \text{X}\left(0\right)={\text{X}}_{0},\\ \text{Y}\left(t\right)=\text{G}\left(X,Y,P,t\right) \end{array}\right.\\ \text{X}=\left(\begin{array}{c} {\text{X}}_{1}\\ \vdots \\ {\text{X}}_{n} \end{array}\right),Y=\left(\begin{array}{c} {\text{Y}}_{1}\\ \vdots \\ {\text{Y}}_{m} \end{array}\right),P=\left(\begin{array}{c} {\text{P}}_{1}\\ \vdots \\ {\text{P}}_{l} \end{array}\right) \end{array}$$

Where *X*(*t*) and *Y*(*t*) are the system variables determined by the functions *F* : *R*^*n* + *m* + *l* + 1^ to *R^n^* and *G* : *R*^*n* + *m* + *l* + 1^ to *R^m^*, respectively, and *P* denotes the system parameters. Note that *Y* can be expressed in terms of *X* and is selected into a separate set only for a clearer physiological interpretation of the model.

The system (1) describes continuous behavior of the model over time *t* and is accompanied by a number of discrete events corresponding to instantaneous changes in the model dynamics (for example, transition from systole to diastole). The event consists of a trigger and assignments. The trigger is the logical function *T* : *R*^*n*+*m*+*l*+1^ → {*true*, *false*}. The event is considered triggered at the time point *t’* if the value of this function changes from *false* to *true* at *t* = *t*′:


$$\begin{array}{c} \exists \delta >0:\forall \text{t}\in \left[{\text{t}}^{{}^{\prime }}-\delta ,{\text{t}}^{{}^{\prime }}\right)\text{T}\left(\text{X}\left(t\right),\text{Y}\left(t\right),P,t\right)\\ =\mathrm{false}\&\text{T}\left(\text{X}\left({\text{t}}^{{}^{\prime }}\right),\text{Y}\left({\text{t}}^{{}^{\prime }}\right),P,{\text{t}}^{{}^{\prime }}\right)=\text{true} \end{array}$$

The event assignments are defined by the functions *A*_*X*_ : *R*^*n*+*m*+*l*+1^ to *R^n^* and *A*_*P*_ : *R*^*n*+*m*+*l*+1^ to *R^l^* describing changes in *X* and *P*, respectively:


(2) $$\left\{ \begin{array}{c} {\text{X}}^{\prime }={\text{A}}_{X}\left(\text{X}\left({\text{t}}^{\prime }\right),\text{Y}\left({\text{t}}^{\prime }\right),P,{\text{t}}^{\prime }\right),\\ {\text{P}}^{\prime }={\text{A}}_{P}\left(\text{X}\left({\text{t}}^{\prime }\right),\text{Y}\left({\text{t}}^{\prime }\right),P,{\text{t}}^{\prime }\right) \end{array}\right.$$

If any event is triggered at the time point *t*′, then solving of the system (1) is automatically stopped, the event assignments (2) are performed, and solving of the Cauchy problem is restarted from *t* = *t*′ with new initial values *X*(*t*′) = *X*′ and new parameter values *P*   = *P*′.

Such models, coupling the continuous and discrete approaches, are called the hybrid models (Stéphanou and Volpert, 2016). Parameters of the Cauchy problem (1) changed by the events (2) constitute the set of the model variables together with *X* and *Y*.

We consider the hybrid model to be in an equilibrium state at *t* = *t*_*SS*_, if values of some variables *Q*_1_, …, *Q*_*k*_ (called equilibrium variables) does not change in time: ∀*t* > *t*_*SS*_*Q*_*i*_(*t*) = *Q*_*i*_(*t*_*SS*_), *i* = 1, …, *k*. It is clear that not every such model with discrete events would have an equilibrium state. For instance, in chaotic triggering of some event, holding the equality *F*(*X*, *Y*, *P*, *t*) = 0 at *t* = *t*_*SS*_ does not guarantee its holding for all *t* > *t*_*SS*_. In the case of modeling the cardiovascular system, discrete events represent switching between systole and diastole stages. Therefore, the model tends to fall into periodic behavior with a period equal to the cardiac cycle length. Thus, while most variables in *X* have non-zero derivatives, some variables in *P* changed by events are in equilibrium. For example, systemic arterial pressure is the dynamic variable dependent on differential equations. This variable increases in systole and decreases in diastole. At the same time, values of systolic and diastolic blood pressures are calculated at the moment of switching between those two stages. These quantities are in equilibrium (their value does not change in time).

To find numerical solutions of the direct problems, we applied the VODE solver (Brown et al., 1989; Cohen and Hindmarsh, 1996) supporting the automatic detection of time points at which discrete events are triggered.

### Modular Modeling

Modularity could be considered as a principle of biological organization (Hartwell et al., 1999; Alon, 2003). Therefore, a modular approach to the modeling of complex biochemical systems has been actively developing in the last few years (Blinov et al., 2008; Hernández et al., 2009; Neal et al., 2014).

**FIGURE 1:**
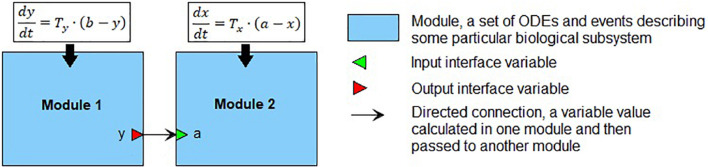
An example of the modular representation of a two-equation model. Module 1 includes one ODE for *y* (output interface variable), as well as two parameters *b* and *T*_*y*_. Module 2 comprises one ODE for *x*, one input interface variable *a*, and one parameter *T*_*x*_.

We define a module (Figure 1) as a part of a mathematical model describing a particular biological subsystem formulated as a separate block and integrated with the rest of the model using an explicit interface. Generally, modules can be treated as separate models with arbitrary mathematical formalism (ODE, stochastic, agent-based model, etc.) aggregated into modular models representing the larger biological systems. Such modules can also contain modular models, thus, forming a hierarchical structure with several nesting levels of modules. Every module defines interface variables used to connect modules with each other. In our case, the interface is defined using mathematical variables and parameters of the module. The input variables serve as the module parameters, these values must be calculated in other parts of the model and then passed to the module. The output variables are calculated inside the module but can be used outside it. Established connections in the modular model show which variables should be passed from one module to another. In the current study, we consider only modules containing sets of ODEs and discrete events (i.e., hybrid models described earlier). In that case, the modular model can be transformed into a “flat” hybrid model with the same formalism by aggregating all equations and events from all modules and resolving connections. For more details, see Kutumova et al. (2012) and Kiselev and Kolpakov (2013).

### Agent-Based Modeling

When modules function in significantly different time scales, an agent-based approach can be used to optimize the calculations. Applications of this approach span a broad range of areas from modeling the adaptive immune system to predicting the spread of epidemics (Macal and North, 2010). An agent is an autonomous entity which acts independently according to certain rules and interacts with other agents. Essentially, the agent is a black box that receives a signal, sends a response and has specific mathematical formalism and numerical methods inside.

When the agent-based approach is used for simulation of the modular models, each module is considered as the separate agent consisting of some mathematical model, numerical solver and time span (initial time, time step, and completion time). Coordination between agents is provided by a scheduler. The agent step corresponds to the model simulation from the current span point to the next one. Before a new step, the scheduler notifies the agent about last changes made to its input interface variables. When the step is finished, the agent sends to the scheduler the changes that it made to the output interface variables. Note that the agent spans are used only to determine time points of the agent interactions, while numerical calculations inside each agent involve automatic computation of time steps necessary for required accuracy.

The agent-based simulation goes as follows (scheme 1).

(1) Pick the agent with the least current model time value.
(2) Pass to the selected agent the changes done by other agents to its input interface variables.
(3) Perform the step in time for the selected agent (i.e., run simulation of the mathematical model incorporated in the agent).
(4) Send the changes made by the selected agent to its output interface variables to the scheduler (for further translation to other agents).
(5) Check if the simulation is finished. If not, go to 1.

The problem arises when two agents have drastically different time scales. For example, we have the “Cardiovascular system” module describing very fast processes of the heart pumping and blood flow across the vascular system, and the “Renal system” module with the long-term regulation of salt-water balance, total blood volume and hormone levels. If we combine both modules into one ODE system, we get a very stiff problem. When using the agent-based form of the model, we still have the problem related to the simulation of the “fast” model over a very large time span, which is very time consuming. However, if the “fast” model has an equilibrium state, we can use another approach to simulation of the agent-based models.

Let’s consider the model with two agents shown in Figure 1. For numerical simulation we will use time span *t*_0_, *t*_1_, *t*_2_, …, *t*_*N*_, where *t*_*i* + 1_ = *t*_*i*_ + Δ, *i* = 1, …, *N*. At each time point *t*_*i*_, the agent corresponding to Module 1 calculates a new value *y*(*t*_*i*_) and sends it to Agent 2 where it is used as a new value for the parameter *a*. Then, Agent 2 starts the next simulation step with the value *a* = *y*(*t*_*i*_). Exact solution of the agent-based model would be the exact solution of the ODE system represented by the “flat” version of the model. Thus, using the agent based approach is equivalent to solving both equations separately between the agent exchanges. That means that when solving the equation for *x* at each time step, we use the numerical value of *y* from the previous time point and introduce the local error *O*(Δ^2^). Let $\bar{x}\left(t\right)$ and $\bar{y}\left(t\right)$ be the simulation results of the flat model, whereas *x*(*t*) and *y*(*t*) are the simulation results of the agent-based model. Suppose, we have no error at the time point *t*_0_:


$$\text{x}\left({\text{t}}_{0}\right)=\bar{\text{x}}\left({\text{t}}_{0}\right),\text{y}\left({\text{t}}_{0}\right)=\bar{\text{y}}\left({\text{t}}_{0}\right)=\text{a}$$

Using the Taylor series for both solutions, we obtain:


$$\bar{\text{x}}\left({\text{t}}_{0}+\Delta \right)=\bar{\text{x}}\left({\text{t}}_{0}\right)+\Delta {\text{T}}_{X}\left(\bar{\text{y}}\left({\text{t}}_{0}\right)-\bar{\text{x}}\left({\text{t}}_{0}\right)\right)+\text{O}\left(\Delta^{2}\right)$$

$$\text{x}\left({\text{t}}_{0}+\Delta \right)=\text{x}\left({\text{t}}_{0}\right)+\Delta {\text{T}}_{X}\left(\text{a}-\text{x}\left({\text{t}}_{0}\right)\right)+\text{O}\left(\Delta^{2}\right)$$

When considering that $\bar{y}\left(t_{0}\right)=a$, we derive:


$$\text{x}\left({\text{t}}_{0}+\Delta \right)=\bar{\text{x}}\left({\text{t}}_{0}+\Delta \right)+\text{O}\left(\Delta^{2}\right)$$

**FIGURE 2:**
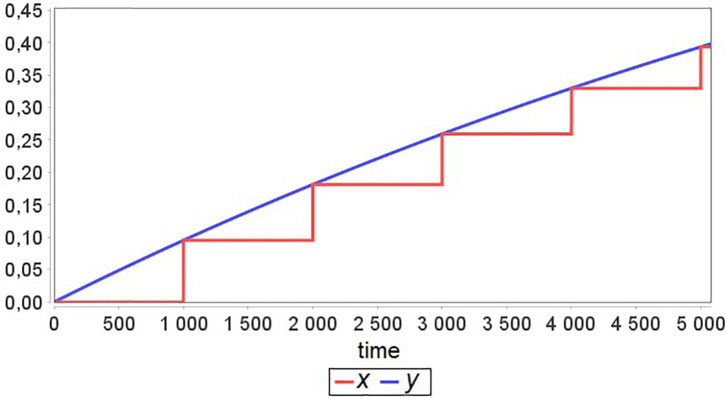
A simulation example for the model defined in Figure 1 with *T*_*x*_≫*T*_*y*_ and time Δ = 1000 between agent interactions.

Now let’s suppose that *T*_*x*_≫*T*_*y*_ and Agent 2 has an equilibrium point for all values of parameter *a*, i.e., if at some *t* = *t*_*i*_, the model is in equilibrium *x*(*t*_*i*_) with *a* = *a*_1_, then changing the value of *a* to *a*_*2*_ and solving the Cauchy problem for *x* with the new initial value equal to *x*(*t*_*i*_) gives new equilibrium *x*(*t*_*i*_ + *t*′). An example result of such model simulation is presented in Figure 2. One can see that after each exchange between agents, the “fast” agent rapidly approaches equilibrium. Therefore, this agent simulation until the next moment of exchange becomes excessive and unnecessary.

A way to optimize scheme 1 for the numerical calculation in this particular case is to stop simulation for the “fast” agent after it reaches the equilibrium at each agent step. Thus, at each time point *t*_*i*_, we set:


(3) $$\text{x}\left({\text{t}}_{i}\right)=\text{x}\left({\text{t}}_{i-1}+{\text{t}}^{{}^{\prime }}\right)$$

Where *t*′ is the time interval during which Agent 2 reaches the equilibrium after the exchange at the time point *t*_*i*–1_.

There is also a way to get a smaller error and simultaneously keep a large time step between agent exchanges. Obviously, the time step of agents Δ is much larger than the time interval *t*′. Therefore, we would introduce a smaller error if we use a new equilibrium value for the previous span point rather than the next one. Instead of (3), we set:


$$\text{x}\left({\text{t}}_{i}\right)=\text{x}\left({\text{t}}_{i}+{\text{t}}^{{}^{\prime }}\right)$$

Applying both described optimizations, we get scheme 2 for the agent modeling:

(1) Pick the agent with the least current model time value. “Fast” agents have a lower priority and, thus, should perform their steps after “slow” agents.
(2) If the selected agent is “slow,” use scheme 1.
(3) Pass to the selected agent the changes done by other agents to its interface variables (For the model in Figure 1: *a*   = *y*_*i*_).
(4) Perform the step in time for the selected agent (i.e., run simulation of the mathematical model incorporated in the agent).
  (a) If the agent is “slow”, perform the numerical calculations until the model reaches the next time step *x*(*t*_*i*_) → *x*(*t*_*i* + 1_) = *x*(*t*_*i*_ + Δ).
  (b) If the agent is “fast”, perform the numerical calculations until the model reaches the equilibrium *x*(*t*_*i*_) → *x*(*t*_*i*_ + *t*′). Set new value for the current time point *x*(*t*_*i*_) = *x*(*t*_*i*_ + *t*′). Set the model time of the agent to the next span point *t* = *t*_*i* + 1_ (thus, the value of *x*(*t*_*i* + 1_) will be calculated at the next step).
(5) Send changes made by the selected agent to its interface variables to the scheduler (for further translation to other agents).
(6) Check if the simulation is finished. If not, go to 1.

**FIGURE 3:**
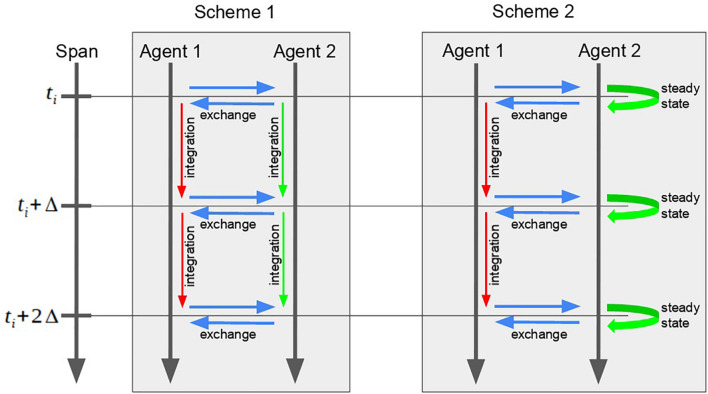
Comparison between two schemes of the two-agent model simulation. The second scheme, instead of the long and unnecessary numerical calculations of the “fast” Agent 2, searches for the equilibrium at each point of the time span.

Comparison between both schemes of the simulation is given in Figure 3.

### Parameter Estimation

Parameter estimates performed in this work for the model calibration were based on minimization of the distance function defined as a normalized sum of squared differences (Hoops et al., 2006) between simulated equilibrium values *Q*_1_, …, *Q*_*k*_ and clinical measurements $Q_{1}^{exp},\ldots ,Q_{k}^{exp}$of physiological quantities:


(4) fdist=∑i=1kωminωi(Qi(tss)-Qiexp)2,ωi=Qiexp

Where ω_*min*_ = *min*_*i*_ω_*i*_ and weights ω_*i*_ are used to make all quantities to have similar importance.

To keep certain model variables *W*_1_(*t*), …, *W*_*p*_(*t*) in physiological limits, we considered known constraints $W_{i}^{min}\leq W_{i}\left(t\right)\leq W_{i}^{max}$ and additionally minimized the penalty function (Runarsson and Yao, 2000):


(5) $$\begin{array}{c} f_{\mathrm{penalty}}=\sum_{t}(\sum_{i=1}^{P}\max\{0,W_{i}^{\min}-W_{i}\left(t\right){\}}^{2}\\ +\sum_{i=1}^{p}\max\{0,W_{i}\left(t\right)-W_{i}^{\max}{\}}^{2}) \end{array}$$

Calculated for *t* ∈ [*t*_*ss*_, *t*_*ss*_ + *d*], where *t*_*SS*_ is the model equilibrium time point and *d* is the length of the cardiac cycle. Note that, as mentioned above, though the model is in equilibrium, some of its variables (e.g., arterial pressure) are dynamic and change their values during the cardiac cycle due to the presence of discrete events.

The process of parameter fitting was based on the stochastic ranking method suitable for the constrained optimization (Runarsson and Yao, 2000).

### Modeling Platform

BioUML (homepage)^2^ is an integrated Java platform for modeling of biological systems (Kolpakov et al., 2019). It supports a comprehensive range of tools for systems biology, including visual modeling, simulation, parameter estimation and a number of numerical methods. Key features of the software used in this work:

• The opportunity to work independently in the local (standalone) version of the program or through the web interface in collaboration with other researchers.
• Plugin-based architecture of the platform allowing to design new types of models and to implement required methods for the numerical analysis of them.
• An editor for visual modeling of biological systems using modular and agent-based approaches.
• The embedded VODE solver (Cohen and Hindmarsh, 1996) ported to Java and suitable for the numerical simulation of hybrid models with ODE systems and discrete events.
• A number of embedded methods (in particular, the stochastic ranking evolution strategy (Runarsson and Yao, 2000) which we preferably used in this study) for the model parameter fitting based on the reference ranges of physiological parameters in normal and pathological states.
• Integration with the JupyterHub^3^ for interactive data analysis.
• Support of the SBML standard (Hucka et al., 2019) for model exchange.

### Visual Modeling

A visual approach to mathematical modeling involves the creation and work with mathematical models as diagrams. Thus, each element of the model (equation, event, interface variable, agent, connection) corresponds to an element of the diagram: edge or node. The visual modeling implies using some kind of formal graphical notation, so the visual representation of each particular element depends on their mathematical properties. A common standard for the graphical notation in systems biology is SBGN (Systems Biology Graphical Notation, Le Novère et al., 2009). However, it is mostly used for mathematical models comprising pathways of processes (e.g., biochemical reactions) and it lacks the elements representing the arbitrary differential or algebraic equations. Thus, in the current study, we use the visual notation developed in the BioUML platform. The visual representation makes the inner structure of the model more explicit and facilitates understanding of the model.

## Results

### Modular Structure of the Model

The comprehensive model of the human cardiovascular and renal systems is based on the range of models of renal hemodynamics (Karaaslan et al., 2005, 2014; Hallow et al., 2014; Hallow and Gebremichael, 2017a) originating from the Guyton model (Guyton et al., 1972), and the model of blood circulation system (Proshin and Solodyannikov, 2006). Below we provide a brief description of the modular representation, while details of the equations, parameters and variables for each module are given in the Supplementary Appendix Tables A1–A4. Totally, the model contains 20 modules, 10 discrete events, 185 equations, 132 parameters, and 160 variables.

**FIGURE 4:**
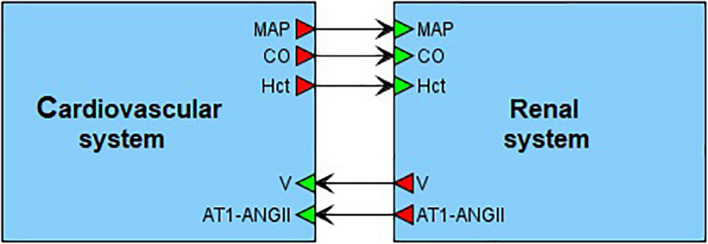
Modular modeling of the cardiovascular and renal systems. The original model decomposed into two modules in accordance with human physiology. The “Cardiovascular system” module is responsible for calculation of such variables as mean arterial pressure (MAP) and cardiac output (CO). The “Renal system” module in turn regulates total body blood volume (V) and concentration of angiotensin II bound to the AT1 receptors (AT1-ANG II). The value of hematocrit (Hct) is constant. It is determined in “Cardiovascular system” and passed to “Renal system” for use.

On the top level, the model can be introduced as an interaction of two main agents: the renal dynamics is determined in minutes, whereas the cardiovascular processes take a fraction of a second (Figure 4). These agents have five main connections corresponding to physiological quantities that are calculated in one agent and directly affect the dynamics of another one. In the cardiovascular system, such quantities are mean arterial pressure, cardiac output and a value of hematocrit. The renal system is responsible for regulation of the total body blood volume and concentration of angiotensin II bound to the AT1 receptors.

**FIGURE 5:**
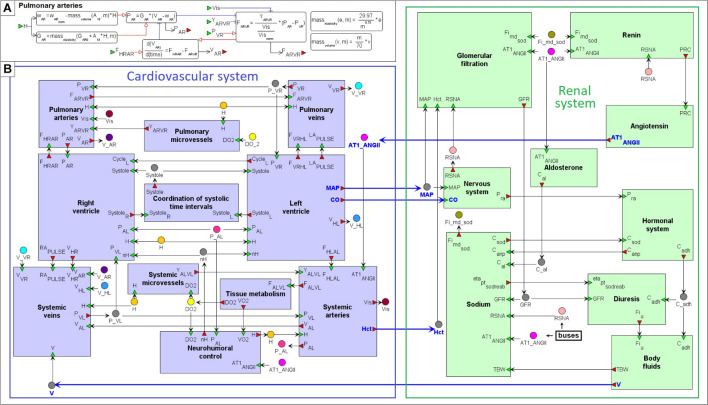
Modular agent-based physiological model of the cardiovascular and renal systems. **(A)** An example of the “Pulmonary arteries” module representation. **(B)** The model implemented in the BioUML platform is divided into nine modules responsible for the kidney function (green) and 11 modules that simulate dynamics of the cardiovascular system (purple). Blue arrows indicate directed connections between the renal and cardiovascular sub-diagrams (Figure 4). For visual simplicity of the diagram, we added transitional nodes (busses) which are used for connections between modules. The busses corresponding to one variable can be located far apart in the diagram. A complete list of equations for each module is given in the Supplementary Appendix Table A2.

For clarity, the model can be further decomposed into 20 functional modules: 11 in the cardiovascular sub-model and 9 in the renal sub-model (Figure 5). Six main cardiovascular modules form a circular system of compartments (Proshin and Solodyannikov, 2006): *Left ventricle*, LV (denoted *HL* in the model notation), *Systemic arteries* (*AL*), *Systemic veins* (*VL*), *Right ventricle*, RV (*HR*), *Pulmonary arteries* (*AR*), and *Pulmonary veins* (*VR*). Each *i*-th compartment, *i* ∈ {*HL*, *AL*, *VL*, *HR*, *AR*, *VR*}, is characterized by pressure *P*_*i*_, volume *V*_*i*_, unstressed volume ω_*i*_, and elastance *G*_*i*_. Blood flow *F*_*ij*_ between the *i*-th and *j*-th compartments is determined by the difference of their pressures, and can be simply written as:


$${\text{F}}_{\mathrm{ij}}={\text{Y}}_{\mathrm{ij}}\left({\text{P}}_{i}-{\text{P}}_{j}\right)$$

Where *Y*_*ij*_ is conductivity of the respective part of the circulatory system. Thus, an important variable affecting blood flow from arteries to veins is conductivity of microvessels (arterioles, capillaries and venules). We allocated the calculation of this variable for systemic and pulmonary circulation into separate modules “*Systemic microvessels*” and “*Pulmonary microvessels*,” respectively.

One of the main ideas of the model constructed by Proshin and Solodyannikov (2006) is the use of discrete events that determine the instantaneous change in the parameters of the left and right ventricles at the moment of a cardiac cycle start (diastole to systole transition), and at the moment when blood ejection from the ventricles stops. Since the LV and RV ejection time is different (Leighton et al., 1971; Hirschfeld et al., 1975), the model contains 2 discrete events of systole to diastole transition in the LV and RV modules, respectively. Each of these 2 events, in particular, changes the value of the corresponding indicator *Systole*_*L*_ or *Systole*_*R*_ from 1 (ejection is in progress) to 0 (ejection is finished). The common indicator *Systole* tracing the moment when blood ejection from both ventricles comes to the end is placed into the module “*Coordination of systolic time intervals*.” It is equal to 0 if *Systole*_*L*_ = 0 and *Systole*_*R*_ = 0, and 1 otherwise.

A model of oxygen exchange describing oxygen consumption in tissues and oxygen debt based on the amount *F*_*ALVL*_ was taken unchanged from the work (Proshin and Solodyannikov, 2006) and inserted into the module “*Tissue metabolism*.”

The last module “*Neurohumoral control*” in the heart sub-model defines the cardiac center as a control system forming the output value of a hypothetical neurohumoral factor *H* from the sum signal of receptor activities. The model by Proshin and Solodyannikov (2006) considers four types of receptors, namely stress (*nS*), weariness (*nD*), and respiratory receptors (*nV*), as well as baroreceptors (*nB*). Receptors respond to various internal factors of the body and external influences and transmit to the cardiac center the signals calculated by the formulas:


$$\begin{array}{c} \mathrm{nB}={\text{r}}^{-}\left({\text{P}}_{AL}\right),\mathrm{nV}={\text{r}}^{-}\left({\text{VO}}_{2}\right),\\ \mathrm{nD}={\text{r}}^{+}\left({\text{DO}}_{2}\right),\mathrm{nS}={\text{r}}^{+}\left(\mathrm{st}\right) \end{array}$$

Where *P*_*AL*_ is systemic arterial pressure (determined in the module “systemic arteries”), *VO*_*2*_ and *DO*_*2*_, respectively denote venous oxygen content and oxygen debt (“tissue metabolism”), *st* ∈ [0,1] define the level of steroid hormones in the blood, whereas *r*^−^(φ) = 1−*r*^+^(φ) and *r*^+^(φ) is the sigmoid function:


$${\text{r}}^{+}\left(\phi \right)={\text{r}}^{+}\left(\alpha ,\beta ,\phi ,\phi_{0}\right)=\frac{1-\text{exp}\left(-\alpha \left(\phi -\phi_{0}\right)\right)}{1+\beta \cdot \text{exp}\left(-\alpha \left(\phi -\phi_{0}\right)\right)}$$

The initial values of the constants α, β, and φ_0_ for all receptors are listed in the study (Proshin and Solodyannikov, 2006).

The renal sub-model incorporates the renin-angiotensin-aldosterone system (RAAS) pathway divided into three main modules:

• Secretion of renin (the “*Renin*” module).
• Generation of angiotensin I with consistent formation of angiotensin II, -IV, -(1–7), and activation of AT1/AT2 receptors (“*Angiotensin*”).
• Secretion of aldosterone (“*Aldosterone*”).

Dynamics of these modules depends on the renal sympathetic nerve activity calculated in the module “*Nervous system*,” whereas concentration of AT1-bound angiotensin II directly effects on tubular sodium reabsorption (*“Sodium”*) and renal vascular resistance (composed of resistances of afferent/efferent arterioles, and interlobar/arcuate/interlobular arteries), as well as renal blood flow and filtration (“*Glomerular filtration*”). The remaining modules are responsible for the calculation of atrial natriuretic peptide and antidiuretic hormone concentrations (“*Hormonal system*”), urine flow (“*Diuresis*”), and volumes of total body water, blood, and extracellular fluid (“*Body fluids*”).

### Details and Updates to the Base Models

Implementation of the model based on parts of the existing models required us to make a number of changes to them with the following goals:

• Relation of these parts with each other.
• Inclusion of the clinically measurable variables to physiological ranges.
• Getting the ability to reproduce pathologies.

Below we provide a description of the primary physiological processes involved in the model, while the detailed formulas can be found in Supplementary Appendices A (Table A2), B.

#### Targets of Angiotensin II

Angiotensin II exerts physiologic actions via binding to receptors on cells of different organs (the kidneys, heart, blood vessels, etc.). The leading role in such actions belongs to the AT1 receptors. Based on their localization (Allen et al., 2000), we have the following angiotensin II targets in the model.

• *Vascular Smooth Muscle* Angiotensin II exerts vasoconstrictor effect (Hughes, 1998). Thus, if the concentration of AT1-bound angiotensin II (*AT*1_*ANGII*) increases, then resistances of afferent/efferent arterioles (*R*_*aa*_ and *R*_*ea*_) and interlobar/arcuate/interlobular arteries (*R*_*preglom*_) rise resulting in the renal blood flow decline. Functions of *AT*1_*ANGII* effects on *R*_*aa*_, *R*_*ea*_, and *R*_*preglom*_ were suggested by Hallow et al. (2014). We assumed that the concentration of AT1-bound angiotensin II in the systemic arterioles is the same as that in the kidneys.
• *Cardiac Muscle* Angiotensin II-induced positive chronotropic and slight positive inotropic effects were demonstrated in the isolated dog heart (Kobayashi et al., 1978). At the same time, the action of angiotensin II in humans is associated either with no change in heart rate or with a reduction that is much smaller than that produced by other vasoconstrictors (Reid, 1996). Thus, when modeling the response to antihypertensive therapies, the influence of angiotensin II on cardiac muscle must be taken into account to simulate decrease in blood pressure without increasing heart rate, which, for instance, is the standard effect of such RAAS inhibitors as aliskiren, losartan, and enalapril (Konstam et al., 1993; Kamishirado et al., 1997; González-Abraldes et al., 2001; Stanton et al., 2003; Parrinello et al., 2009; Natarajan et al., 2016). In this regard, we considered in the model two targets of angiotensin II: baroreceptors and stress receptors. Such a decision was based on the facts that angiotensin II resets the baroreflex control of heart rate to a higher pressure (Reid, 1996) and increases the release of norepinephrine from the atria (Brasch et al., 1993).
• *Adrenal Glands* Angiotensin II stimulates aldosterone synthesis in adrenal zona glomerulosa cells via binding to the AT1 receptors (Bandulik et al., 2015). A function for modeling the *AT*1_*ANGII* effect on the aldosterone secretion rate was taken from the models by Karaaslan et al. (2005) and Hallow et al. (2014).
• *Kidney, Proximal Tubules* An increase in the level of *AT*1_*ANGII* leads to the rise of fractional proximal sodium reabsorption. The corresponding formula was found in the model by Karaaslan et al. (2005).
• *Kidney, Juxtaglomerular Cells In vitro* studies have suggested the expression of the AT1 receptors by juxtaglomerular cells. In this regard, there is the concept that angiotensin II directly inhibits renin secretion through a negative feedback. Although this concept has not yet been systematically proven in vivo and can be doubted (Neubauer et al., 2018), the factors affecting renin secretion (macula densa sodium sensing and renal sympathetic nerve activity) are not be sufficient to account for the rise in renin that occurs with therapeutic blockade of the RAAS (Hallow et al., 2014). Thus, following the model by Hallow et al. (2014) we used the formula of the direct negative feedback of AT1-bound angiotensin II on renin secretion introduced in the study (Hallow and Gebremichael, 2017a).
• *Kidney, Mesangial Cells* Angiotensin II provides a reduction in the glomerular filtration coefficient *K*_*FG*_ (Blantz et al., 1976) via decrease in total filtering surface area because of mesangial cell contraction (Schmitt et al., 1998). Angiotensin II receptors on mesangial cells belong to the AT1 subtype (Ardaillou et al., 1999). Thus, for the calculation of *K*_*FG*_, we considered a product of its normal value and a linear function expressing inversely proportional relationship between *K*_*FG*_ and the normalized concentration of *AT*1_*ANGII*.

#### Peak Flow Rates Through the Heart Valves

The rate of the LV filling through the mitral valve in diastole is characterized by two peaks (Boogers et al., 2011; Caudron et al., 2011; Zhang et al., 2019), which can be estimated from the time derivative of the LV volume by cardiovascular magnetic resonance (Maceira et al., 2006a). In normal subjects, the LV inflow is greatest immediately after opening of the mitral valve (early peak), while the left atrial contraction is responsible for smaller inflow (active peak) (Caudron et al., 2011). The moment of the mitral valve opening in the model is the LV transition to diastole, when the pressure in pulmonary veins becomes greater than the pressure in the ventricle. The left atrial contraction can be associated with a positive value of the left atrial pulse wave, proposed in the model (Proshin and Solodyannikov, 2006). The right ventricular filling through the tricuspid valve has similar dynamics (Maceira et al., 2006b). As for the transaortic and transpulmonary flows, they reach their maximum values at the transition from diastole to systole. Therefore, calculation of the peak transvalvular flow rates can be introduced by the formalism of discrete events.

#### Physiological Quantities

In our work, we strived to create a model focused not on average values of physiological parameters, but on a variety of values including reference intervals for healthy people and possible pathological deviations from the norm. Since the baseline models did not accept these ranges as valid for some variables, we used experimental formulas obtained in population studies for the following variables.

• *Plasma sodium concentration* is calculated by the formula derived in the study (Nguyen and Kurtz, 2003) and obtained on the basis of the Edelman equation (Edelman et al., 1958).
• *Antidiuretic hormone concentration* is defined depending on serum osmolality (Hammer et al., 1980), which value can be considered as function of plasma sodium concentration and levels of glucose and urea in blood (Dorwart and Chalmers, 1975; Bhagat et al., 1984).
• *Body fluids*: For the calculation of the total blood volume and the extracellular fluid volume we applied the regression functions on the total blood water (Moore, 1967).
• *Tubular water reabsorption rate* depends on the glomerular filtration rate. In the absence of significant amounts of poorly reabsorbable solutes, the fraction of the water load passively reabsorbed in the proximal tubule is equal to the fraction of the sodium load reabsorbed (Uttamsingh et al., 1985). Taking into account details of laboratory measurements, this fraction also includes sodium reabsorbed in the loop of Henle (Seidlerová et al., 2006). The rate of fluid reabsorption from the distal tubules and collecting ducts depends on the influence of plasma vasopressin concentration, which was determined in the Uttamsingh model (Uttamsingh et al., 1985) on the basis of experimental measurements (Dehaven and Shapiro, 1970).
• *Blood viscosity* is calculated according to the study (Hund et al., 2017) as a function of hematocrit. We took into account that values of blood flows through the systemic and pulmonary microvessels should be inversely proportional to the normalized blood viscosity (Guyton et al., 1972).

#### Aldosterone Secretion

When modeling the effects of potassium and sodium on the secretion of aldosterone, we considered the following facts:

• Extremely low potassium levels (2 mmol/l) actually reduce aldosterone production stimulated by angiotensin II, but does not stop it completely (Kojima et al., 1985; Chen et al., 1999).
• A low-sodium diet does not directly affect the aldosterone secretion, but indirectly through activation of the RAAS, upregulation of AT1 receptor levels, and hyperplasia of the zona glomerulosa (Bollag, 2014).
• A rise of 1 mmol/l in serum potassium concentration doubles the aldosterone secretion (Pralong et al., 1992; Bollag, 2014).

#### Glomerular Dynamics

The total vascular resistance through the kidneys (*RVR*) is determined by the sum of the resistances in the individual vasculature segments (Hall, 2011). Table 1 shows the different variants of the formula for the *RVR* calculation used in the different studies. Note, that Gómez (1951) gives out the major renal resistances which can be estimated from clinically available data. Therefore, following this research, we used the same formula for *RVR* and applied it to derive an equation for renal blood flow (Supplementary Appendix B). We also included to the model such clinical variables as effective renal plasma flow, filtration fraction, and total protein (Škrtić et al., 2015).

**TABLE 1:**
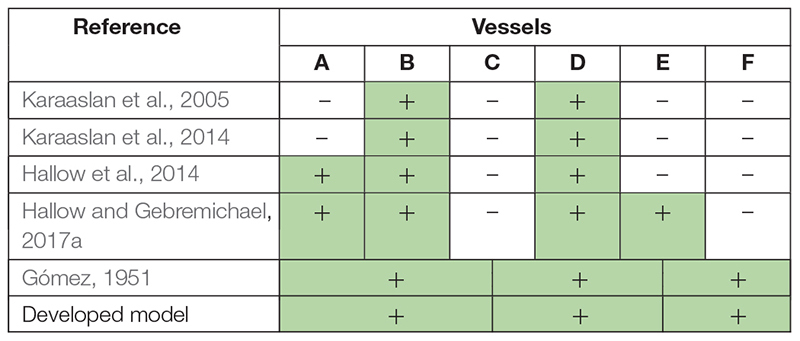
The *RVR* determination as the sum of the resistances of separate vessels: A, interlobar, arcuate, and interlobular arteries; B, afferent arterioles; C, glomerular capillaries; D, efferent arterioles; E, peritubular capillaries; F, interlobar, interlobular, and arcuate veins.

*The parts of RVR suggested by Gómez (1951) can be evaluated clinically. Thus, we used the same division in our model. The individual vasculature segments taken into account in the corresponding studies are marked with a plus sign and colored green.*

### Starting Values of Variables

Each equilibrium parameterization of the model can be considered as a unique virtual patient (Cheng et al., 2017). To deal with a variety of such patients (which is related to the search for different equilibriums of the model depending on the starting values), we need to have a tool for scaling of physiological variables. For this purpose, Proshin and Solodyannikov (2006) used the general scaling scheme representing the dependence of biological variables *a*_*i*_ (elasticity of vascular walls, conductivity of vessels, body oxygen demand, etc.) on body mass *m* by an allometric power-laws (West et al., 1997; Proshin and Solodyannikov, 2006):


$${\text{a}}_{i}={\text{c}}_{i}\cdot m^{b_{i}}$$

Where *b*_*i*_ are the scaling exponents and *c*_*i*_ are the normalization constants. In addition to this approach, we took into account the approximate distribution of blood (in percentage of total blood *V*) in the different parts of the circulatory system: 84% – systemic circulation (including 13% in systemic arteries) (Hall, 2011), 10% – pulmonary circulation (Gazioglu and Yu, 1967), 6% – heart. Pulmonary vessels include arterial (35% of pulmonary circulation), venous (45%) and capillary (20%) volumes (Gazioglu and Yu, 1967). Since in the model by Proshin and Solodyannikov (2006) pulmonary veins and capillaries are included in one compartment of the circulatory system, assuming equality of the starting *V*_*HL*_ and *V*_*HR*_, we get the following formulas for calculating the initial volumes of the compartments:


$$\begin{array}{c} {\text{V}}_{HL}=0.03\cdot \text{V},{\text{V}}_{\mathrm{AL}}=0.13\cdot \text{V},\\ {\text{V}}_{\mathrm{HR}}=0.03\cdot \text{V},{\text{V}}_{\mathrm{AR}}=0.035\cdot \text{V} \end{array}$$

$$\begin{array}{c} {\text{V}}_{\mathrm{VR}}=0.065\cdot \text{V},\\ {\text{V}}_{\mathrm{VL}}=\text{V}-V_{\mathrm{AL}}-{\text{V}}_{\mathrm{AR}}-V_{\mathrm{HL}}-{\text{V}}_{\mathrm{HR}}-{\text{V}}_{\mathrm{VR}} \end{array}$$

The corresponding unstressed volumes we determined as:


$$\omega_{i}={\text{k}}_{i}\cdot {\text{V}}_{i},\text{i}\in \left\{\mathrm{HL},\mathrm{AL},\mathrm{VL},\mathrm{HR},\mathrm{AR},\mathrm{VR}\right\}$$

Where estimated constants *k*_*AL*_, *k*_*VL*_, *k*_*AR*_, *k*_*VR*_ ∈ [0.7, 1.0], and *k*_*HL*_, *k*_*HR*_ ∈ [0.0, 0.3] (see the Supplementary Appendix Table A3 for details of such intervals selection).

### The Model Calibration

Many parameters of the model correspond to physiological quantities, which values can be evaluated by the laboratory measurements. Thus, the model calibration consists in finding an equilibrium state satisfying the set of physiological constraints. The explicit form of these constraints depends on the studied problem and the status of the simulated patient (healthy or sick). For example, the normal range of the systolic/diastolic pulmonary artery pressure is 15–30/4–12 mmHg (Pagani et al., 1988). In patients with class II heart failure according to the New York Heart Association classification, the corresponding values are higher: 35.4 ± 8.8/14.4 ± 5.8 mmHg (Murch et al., 2015), whereas in patients with pulmonary arterial hypertension these values can achieve 84.0 ± 23.0/37.0 ± 13.0 mmHg (Gan et al., 2006).

In this work, we present the model’s ability to simulate healthy subjects as well as patients with the most common cardiovascular diseases. We considered the following test cases:

• Patient 1: Uncomplicated hypertension (Ferlinz, 1980).
• Patient 2: non-hypertensive diastolic heart failure (Fujimoto et al., 2008).
• Patient 3: Hypertension and LV hypertrophy without heart failure (Melenovsky et al., 2007).
• Patient 4: Hypertensive diastolic heart failure without LV hypertrophy (Fujimoto et al., 2008).
• Patient 5: Pulmonary hypertension and left heart disease (Wright et al., 2017).

The model calibration included two main steps.

(1) *Search for the equilibrium state matching physiology values in a healthy human*: We considered the normal value ranges for clinically measurable quantities used in the model. Such quantities cover 49 of 132 model parameters (Supplementary Appendix Table A3) and 69 of 160 model variables (Supplementary Appendix Table A4). The remaining parameters and variables of the model either cannot be measured in the laboratory, or we could not find available data to estimate their ranges. Then, 132 parameters of the model were determined in the following way.
  (a) The values of 92 parameters were taken from the basic models (Guyton et al., 1972; Karaaslan et al., 2005; Proshin and Solodyannikov, 2006; Paeme et al., 2011; Hallow et al., 2014; Hallow and Gebremichael, 2017a; Rosalina et al., 2019) or found in the research articles (Gómez, 1951; Škrtić et al., 2015; Digne-Malcolm et al., 2016; Hund et al., 2017; Neal et al., 2018).
  (b) The rest 7 new and 33 reused parameters, whose values directly affected the compliance with the normal ranges, were fitted by zeroing of the penalty function (5) using the constrained optimization evolutionary algorithm (see section “Materials and Methods”).
(2) *Calibration of the model to the pathological equilibrium states*.
  (a) We defined a set of 65 fitting parameters, whose values can reasonably vary depending on the diseases of the abstract patients given above (Supplementary Appendix Table C1). These parameters are either directly related to the diseases, and therefore their values can deviate from the norm, or vary within the known normal ranges, if the diseases do not affect them.
  (b) We determined a set of 57 physiological constraints imposed on the model variables in conformity with the clinical measurements in the diseases or in the normal state, if there was no available data confirming the effect of the diseases on these variables (Supplementary Appendix Table C2). Note that at the first step, we considered constraints for 69 clinical variables, whereas at this step the number of constraints is lower. This is due to the fact that we eliminated some of the excessive constraints to accelerate the optimization process. For example, the heart rate is related to the cardiac cycle length. Thus, we took into account the physiological range for the first variable and excluded the range for the second one. The cardiac output is equal to the product of the stroke volume and the heart rate. Therefore, we considered the constraints for the first two variables and skipped the constraints for the last one, etc.
  (c) We chose standard patient characteristics provided in clinical studies of the diseases (references in definition of patients 1–5): age, weight, height, gender, systolic/diastolic blood pressure, heart rate, stroke volume, ejection fraction, and hemoglobin. We used the average values of these characteristics reported in the studies (upon availability): weight and hemoglobin were directly set to the model; weight, height, and gender were taken to evaluate total body water of abstract patients (by the Nadler equation, Nadler et al., 1962); remaining values were used to estimate the corresponding model variables by the minimization of the distance function (4).
  (d) We specified the optimization problem with the set of fitting parameters (a), the penalty function (b), and the objective function (c), and solved this problem as described in the section “Materials and Methods” for each test case.

The resulting equilibrium values of the model within the ranges of a healthy person are listed in the Supplementary Appendix Tables A3, A4. These values are also provided in the BioUML web-implementation of the model. Physiological quantities of the equilibrium states representing abstract patients 1–5 are given in Table 2 and introduced as the model states stored as separate documents and applicable to the model (see the Availability section below).

**TABLE 2:**
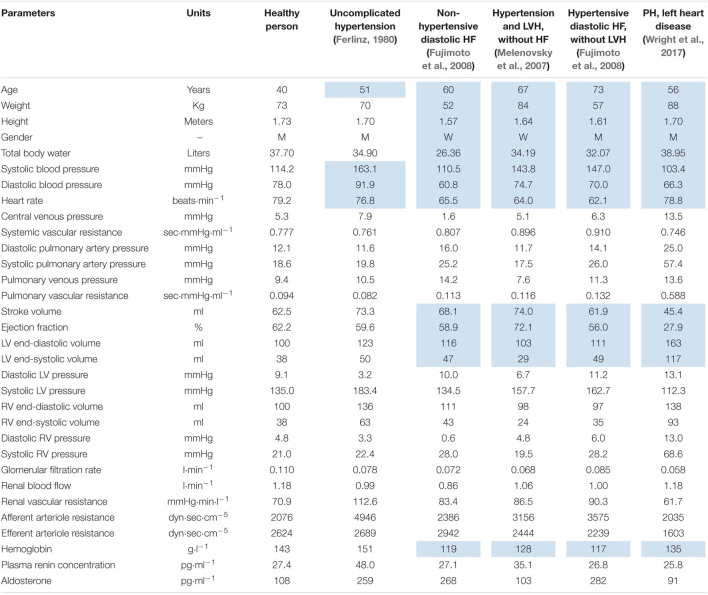
Equilibrium values representing abstract patients with different cardiovascular diseases.

*Values, which we evaluated using average clinical characteristics of patients in corresponding experimental studies, are marked with blue color. LV, left ventricle; RV, right ventricle; HF, heart failure; LVH, left ventricular hypertrophy; PH, pulmonary hypertension.*

### Use Cases

As examples of using the model, we consider two standard problems faced by researchers in the study of human physiology: comparison of different physiological states and comparison of sensitivity of different patients to the change in physiological conditions. To produce the comparative plots, we used the capabilities of the Jupyter notebook embedded in BioUML. This application is designed to create and share documents that contain live code, equations, visualizations and narrative text. The Jupyter files comprising the implementation of both use cases described below are available in the web-version of our software (see the Availability section).

#### Comparison of Different Physiological States

As the typical examples, consider the following:

• Comparison of normal and abnormal physiology (uncomplicated hypertension vs. normal state).
• Comparison of states with the same disease but different underlying pathophysiologic mechanisms (non-hypertensive vs. hypertensive diastolic heart failure).
• Comparison of the pathological states reproducing complex cardiovascular diseases (pulmonary hypertension and left heart disease vs. systemic hypertension and left ventricular hypertrophy).

**FIGURE 6:**
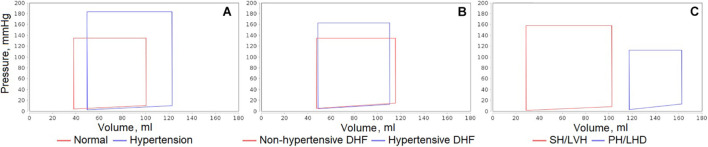
Comparison of left ventricular pressure-volume loops simulated for different equilibrium states of the model. **(A)** Uncomplicated hypertension vs. normal state. **(B)** Non-hypertensive vs. hypertensive diastolic heart failure (DHF). **(C)** Pulmonary hypertension and left heart disease (PH/LHD) vs. systemic hypertension and left ventricular hypertrophy (SH/LVH).

Figure 6 shows the sample plots of the left ventricular pressure-volume loops simulated for all these cases. Similar plots can be automatically generated by the code in JavaScript included into the Jupyter file for any equilibrium states of the model and any variables of interest. Specifically, in the given plots, we can see the dynamics corresponding to the considered patient diseases:

• In the untreated hypertensive patients, left ventricular systolic pressure can be substantially higher than in normal subjects (Antony et al., 1993).
• Left ventricular end-systolic pressure is higher in hypertensive than in non-hypertensive patients with diastolic heart failure, while left ventricular end-diastolic pressure is elevated in the both groups (Fujimoto et al., 2008).
• Patients with congestive heart failure (generally accompanying pulmonary hypertension) can have significantly higher left ventricular end-diastolic and end-systolic volumes (Mehta et al., 2000).

#### Comparison of Sensitivity of Different Patients to the Change in Physiological Conditions

As the test case of this problem, we used the experiment with varying sodium intake presented in He et al. (2001). The study by He et al. (2001) involved a 5-day high sodium diet (≈ 350 mmol/d) followed by a 5-day low sodium diet (10–20 mmol/d) in normotensive and hypertensive individuals.

We designed the corresponding simulation experiment by the following way:

• 0–1 day – the normal diet with initial equilibrium values of sodium intake in the model states.
• 1–6 days – high sodium diet with sodium intake equal to 0.24306 mEq/min ≈ 350 mmol/d.
• 6–11 days – low sodium diet with sodium intake equal to 0.01042 mEq/min ≈ 15 mmol/d.
• 11–21 days – the normal diet returning the model dynamics to the initial equilibrium.

**FIGURE 7:**
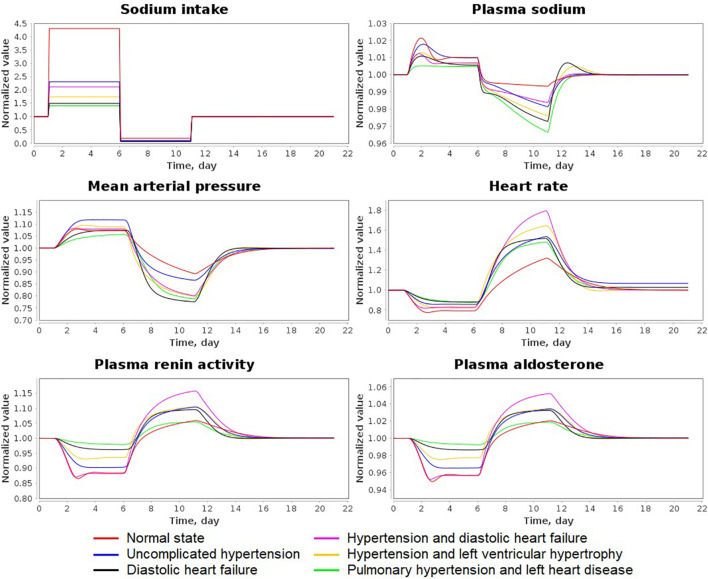
Simulation results of the sodium load experiment for all states of the model. The simulation experiment was designed in accordance with experimental study by He et al. (2001) and involves a 5-day high sodium diet followed by a 5-day low sodium diet. Variable values are normalized to the initial equilibrium values declared in the states.

Figure 7 shows the dynamic results of the model in all states for relative values (normalized to the initial value) of sodium intake, plasma sodium, mean arterial pressure, heart rate, plasma renin activity and aldosterone concentration. Table 3 reveals the exact and relative difference in these values between high (6 days) and low (11 days) salt diets. As can be seen from these data, hypertensive patients have a greater fall in blood pressure from high-salt to low-salt diet than normotensive subjects. This result is consistent with the conclusions by He et al. (2001). However, the laboratory measurements by the authors also demonstrated that supine pulse rate on average did not change with acute salt restriction, whereas plasma renin activity and aldosterone concentration had higher growth in normotensive than in hypertensive individuals. In our simulation test, we observe another dynamic: a decrease in blood pressure is accompanied by an increase in heart rate. Such dynamics is in line with the fact that sodium reduction can increase heart rate (Graudal et al., 2016). For plasma renin activity and aldosterone concentration, we get the results opposite to conclusions by He et al. (2001). The model shows higher growth of these quantities in hypertensive patients. However, while plasma renin activity responses on average are stronger in normotensive than in hypertensive populations (Graudal et al., 2017), it can be weaker in some individual cases (Graudal et al., 2021). Therefore, the generated model states give acceptable dynamics.

**TABLE 3:** Exact and relative difference in sodium intake (mEq/min), plasma sodium (mEq/l), mean arterial pressure (MAP, mmHg), heart rate (HR, beats/min), plasma renin activity (PRA, fmol/ml/min) and plasma aldosterone concentration (PAC, pg/ml) between high (day 6) and low (day 11) salt diets.

Model state	Sodium intake	Plasma sodium	MAP	HR	PRA	PAC
Normal state	−0.23264 (−95.71%)	−2.4 (−1.66%)	−16.1 (−16.66%)	41.4 (66.06%)	4.8 (19.65%)	6.8 (6.61%)
Uncomplicated hypertension	−0.23264 (−95.71%)	−4.1 (−2.84%)	−29.1 (−22.49%)	51.9 (78.89%)	6.9 (22.17%)	17.8 (7.12%)
Diastolic heart failure	−0.23264 (−95.71%)	−4.8 (−3.27%)	−23.0 (−27.69%)	41.6 (72.09%)	3.8 (13.82%)	12.3 (4.67%)
Hypertension and diastolic heart failure	−0.23264 (−95.71%)	−3.3 (−2.29%)	−26.6 (−25.73%)	60.1 (117.23%)	8.2 (31.19%)	27.0 (9.99%)
Hypertension and left ventricular hypertrophy	−0.23264 (−95.71%)	−4.4 (−3.08%)	−28.3 (−26.57%)	51.5 (95.97%)	5.6 (17.99%)	5.9 (5.86%)
Pulmonary hypertension and left heart disease	−0.23264 (−95.71%)	−5.3 (−3.80%)	−21.1 (−25.39%)	47.7 (69.11%)	2.1 (7.71%)	2.4 (2.66%)

## Discussion

The main goal of our investigation is the modeling of a hypertensive human personal response to antihypertensive therapy. Since arterial hypertension is the multifactorial disease, which can take different forms (pulmonary or systemic) and can be complicated by related pathologies, such as heart or renal failure, the modeling process is divided into several steps.

• *Step 1*: *Creation of a mathematical model of human physiology with a level of detail sufficient to study the issues of arterial hypertension*. True personalization of drug therapies should rely on a virtual patient, the digital twin of a real individual, which is formed and accumulated throughout his life as a result of interaction with the health care system (Lehrach, 2016). We suppose that it is unrealistic to build a virtual patient for all occasions now. Thus, our approach is to construct a set of basic modules (blocks) and assemble a model from them (as from Lego blocks) for a given patient and disease.
• *Step 2*: *Pharmacokinetic/pharmacodynamic (PK/PD) modeling of antihypertensive drugs*. First-line antihypertensive medications include angiotensin-converting enzyme inhibitors, angiotensin II receptor blockers, calcium-channel blockers, thiazide diuretics and β-Adrenoreceptor blockers (Oparil et al., 2018). It is possible to determine the certain points of influence on the model for each of these classes, construct the corresponding PK/PD models and validate unknown dynamic constants using related clinical trials (Hallow et al., 2014).
• *Step 3*: *Personalizing the model*. Each equilibrium parameterization of the model within physiological ranges can be considered as a virtual patient. To relate him with a real person, we can use some values from the laboratory analyses. However, this allows us to get only a small part of the model quantities. To solve the problem with unknown personal parameters, we can build a set of virtual patients and consider significant variation of unknown physiological values. Treatment simulation of such a virtual population makes it possible to identify virtual groups with a similar reaction to the drugs and analyze which features of the patient can contribute to the effectiveness (or ineffectiveness) of the antihypertensive therapy.

This article provides implementation of the first step and presents the model of cardiovascular and renal systems. As the basis for the model construction, we used early published models developed for each of these physiology systems separately (Karaaslan et al., 2005, 2014; Proshin and Solodyannikov, 2006; Hallow et al., 2014; Hallow and Gebremichael, 2017a). Note that the reduction in dietary salt intake leads to a decrease in blood pressure and therefore, can be viewed as a simple antihypertensive therapy (Mahtani, 2009; Frisoli et al., 2012). As follows from Figure 7, the model reproduces the dynamics consistent with this statement. As for the modeling of the individual response to complex therapy including antihypertensive medications with different mechanisms of action (Step 2 and 3), this is a task for future studies.

### The Model Strength

#### Two Physiological Systems Instead of One

The strong connection between renal and cardiovascular disease reflects the complex interactions between heart and kidneys (Stefanadis, 2010). Therefore, it is reasonable to model in detail these body systems together. In relation to the study of personal response to antihypertensive therapies, simultaneous consideration of these systems allows simulating virtual patients with different combinations of heart and renal diseases associated with hypertension. This also provides a tool for modeling the hypotensive effect in response to β-blockers treatment, which is not possible with consideration of the renal function alone (Hallow et al., 2014).

#### Modular Representation

The modular approach facilitates development of complex models by representing them as combinations of submodels (Kiselev and Kolpakov, 2013). The structure of the model is clear and understandable. The model can be easily expanded. The separate modules can be independently modified and improved.

#### Agent-Based Approach

Agent-based models are unique in their ability to integrate combinations of heterogeneous processes and investigate their respective dynamics. These models are flexible in their execution and permit the aggregation of processes across time scales (Glen et al., 2019). So in our case, the cardiovascular system modules are simulated in fraction of seconds which is due to the work of the heart, while the renal system modules are measured in minutes.

#### Wide Range of Parameter Values

Our model is not geared toward parametrizations representing a limited group of healthy people or patients with the same combination of diseases, but has a wide parameter space, which is sufficient for simulation of patients with systemic/pulmonary arterial hypertension and main concomitant cardiovascular diseases.

### The Model Limitations

#### Limitations by the Parameter Constraints

Parameter bounds and constraints, which we collected on the basis of known clinical studies and listed in the Supplementary Appendix Tables C1, C2, are suitable for simulation of a wide range of patients with cardiovascular diseases, but still do not reflect all possible cases and require further extension and systematization to detail physiological ranges depending on patient features.

#### Limitations by the Model Equations

We use the same form of equations to simulate different pathological conditions. However, in some diseases, a part of variables may be outside the acceptable ranges. For example, in the study (Riegger et al., 1982), one group of patients with congestive heart failure had inappropriately high values of plasma antidiuretic hormone (14.5 ± 8.8 pg/ml) in relation to their plasma osmolality, which was well below normal values (276 ± 23 mOsmol/kg water). At the same time, the equation used in the model, does not allow the analysis of osmolality values less than 271 mOsmol/kg. Thus, to simulate such patients, the function of calculation of plasma antidiuretic hormone must be advanced.

### Possible Directions of the Model Development

#### Aging as a Key Factor in Cardiovascular Diseases

Aging-mediated structural and biochemical modifications coupled with gradual loss of autonomic nervous system regulation and vascular stiffening are consistently implicated in the progressive increase in mechanical burden and functional breakdown of the heart and vessels (Fajemiroye et al., 2018). Thus, it seems to us very important to introduce age in the equations modeling the age-dependent variables.

#### Genetic Contribution

Estimated heritability of systolic and diastolic blood pressure lies in the ranges of 15–40% and 15–30%, respectively (Delles and Padmanabhan, 2012). More than 900 known genetic loci indicate that sites for blood pressure control involve various organs, including the kidneys and nervous system (Lin et al., 2020). Therefore, extension of the model to take into account personal data of genetic testing can help resolve the questions regarding individual blood pressure regulation.

#### Personalized Cardiac Electrophysiology Modeling

To personalize the cardiac parameters of the model, it is possible to use data obtained from clinical imaging. However, when imaging data is noisy, an alternative rule-based methodology can be utilized to simulate electrical wave propagation and mechanical contraction in the heart (Bayer et al., 2012; Lopez-Perez et al., 2015; Nguyen et al., 2020). Application of this methodology can be useful for extension the model to involve the personalized anatomy of the heart.

Finally, note that we did not aim to reproduce all possible variations of cardiovascular diseases. Nevertheless, we suppose that the model can be easily extended to any group of patients, depending on the study purpose. Summarizing the above, we believe that the composite model of cardiovascular and renal systems could be useful for further investigation of cardiovascular diseases and drug development. The BioUML implementation of the model is available at: https://gitlab.sirius-web.org/virtual-patient/blood-pressure-regulation.

## Data Availability Statement

The datasets presented in this study can be found in online repositories. The names of the repository/repositories and accession number(s) can be found below: gitlab.sirius-web.org/virtual-patient/blood-pressure-regulation.

## Author Contributions

IK and EK designed the agent-based model of cardiovascular and renal systems, and implemented necessary tools in the BioUML software (agent-based modeling and simulation tools, and optimization tools). EK validated the model based on the clinical data from scientific literature. RS consulted on the physiological questions. GL consulted on the questions of hypertension and antihypertensive therapy, coordinated the medical part of the study. FK coordinated creation of the model and development of BioUML. EK and RS prepared of the model documentation (Supplementary Material). EK, IK, RS, GL, and FK wrote the manuscript. All authors contributed to the article and approved the submitted version.

## Conflict of Interest

EK, IK, RS, and FK were employed by company Biosoft.Ru, Ltd., which develops and supports the BioUML platform. During the research the BioUML platform was improved by authors for the purpose of the research. The remaining author declares that the research was conducted in the absence of any commercial or financial relationships that could be construed as a potential conflict of interest.

## Publisher’s Note

All claims expressed in this article are solely those of the authors and do not necessarily represent those of their affiliated organizations, or those of the publisher, the editors and the reviewers. Any product that may be evaluated in this article, or claim that may be made by its manufacturer, is not guaranteed or endorsed by the publisher.

## Footnotes

1
www.samara-dialog.ru/help/eng/help.htm

2
www.biouml.org

3
jupyter.org
