# Supplementary material for: Thoroughly Calibrated Modular Agent-Based Model of the Human Cardiovascular and Renal Systems for Blood Pressure Regulation in Health and Disease
Source: Front Physiol. 2021 Nov 11;12:746300. doi: 10.3389/fphys.2021.746300 (PMC8632703; doi:10.3389/fphys.2021.746300)
Supplement: Supplementary file 1 [file Data_Sheet_1.pdf]

APPENDIX A. DESCRIPTION OF THE MODEL OF THE HUMAN CARDIOVASCULAR AND RENAL SYSTEMS

Table A1. List of the model mathematical functions.

Functions	Description
$mass_{elasticity}(e,m) = \frac{29.97 \cdot e}{m^{0.75}}$	Allometric scaling formula for calculating wall elasticity of six compartments of the circulatory system depending on the body weight *m* and the average normal elasticity value *e* taking into account the allometric index -0.75.
$mass_{volume}(v,m) = \frac{m}{70} \cdot v$	Formula for allometric scaling relative to the body weight *m* taking into account the average human value of 70 kg. It is used to calculate the theoretical maximum stroke volume $SV_{\max}$, the Frank-Starling law threshold $FS_{threshold}$ and the body oxygen demand RO_2.
$mass_{conductivity}(c,m) = 0.042 \cdot m^{0.75} \cdot c$	Allometric scaling formula for calculating conductivity of individual sections of the vascular bed depending on the body weight *m* and the average normal value *c* taking into account the allometric index 0.75.
$r_{plus}(a,b,x,x0) = \frac{1 - e^{-a\cdot(x-x0)}}{1 + b \cdot e^{-a\cdot(x-x0)}}$	Sigmoid function of receptor activity of the fatigue factor (*nD*), the stress factor (*nS*), and the neurohumoral factor (*H*).
$r_{minus}(a,b,x,x_0) = 1 - r_{plus}(a,b,x,x_0)$	Activity function of respiratory center receptors (*nV*) and baroreceptors (*nB*).
$atrium_{pulse}(T,T_S,P_{AL},t) = \begin{cases} 0, & t \le T - 0.2\cdot(T - T_S) \\ 0.1 \cdot P_{AL} \cdot \lvert T - t \rvert^{1/2}, & else \end{cases}$	The function for calculating values of pulse waves of the left and right atria based on the duration of cycle *T*, actual duration of the left or right ventricular systole T_S, pressure in the systemic arteries P_{AL} and the current time of the cardiac cycle *t*.
$valve(P_{in},P_{out},R_{factor},YY) = \begin{cases} YY\cdot(P_{in} - P_{out}), & P_{in} \ge P_{out} \\ R_{factor}\cdot YY \cdot (P_{in} - P_{out}), & else \end{cases}$	The function for calculating an amount of blood flow through heart valves (aortic, tricuspid, pulmonary and mitral) taking into account the inlet pressure P_{in}, outlet pressure P_{out}, conductivity *YY* and regurgitation coefficient R_{factor}. The second part of the formula describes the reverse blood flow through a valve (regurgitation) in the case of a defect in the locking element ($0 < R_{factor} < 0.3$). In normal conditions, $R_{factor} = 0$.
$sigm(x) = \frac{1}{1+e^{-x}}$	Logistic function.
$\mathrm{sgn}(x,y) = \begin{cases} 0, & x \le 0 \ u \ y \le 0 \\ y, & else \end{cases}$	The function for calculating the oxygen debt level.

Table A2. List of the model equations divided into 20 modules.

№	Equations	Description	Ref
**Left ventricle (LV)**			
001	$\frac{dV_{HL}}{dt} = F_{VRHL} - F_{HLAL}$	Change in blood volume in the LV due to the difference between the input blood flow F_{VRHL} and the output blood flow F_{HLAL}.	Proshin
002	$V_{HL}(0) = 0.03 \cdot V$	Starting value[1] of blood volume in the LV is 3% of the total blood volume V (systemic circulation – 84% [Hall], pulmonary – 10% [Gazioglu], and values $V_{HL}(0)$ and $V_{HR}(0)$ are assumed to be equal).	Hall<br>Gazioglu
003	$K_L = K_{L0} + 0.25 \cdot nH \cdot sigm(20 \cdot (K_{L0} - 0.4)) - $<br>$- 0.25 \cdot nH \cdot sigm(20 \cdot (K_{L0} - 0.7))$	Inotropic factor of the LV. Numerical characteristic of the myocardial inotropism (contractility). K_{L0} – inotropic status of the LV.<br>nH – myocardial sympathetic inotropic sensitivity.	Proshin
004	$\omega_{HL}(0) = k_{HL} \cdot V_{HL}$	Unstressed LV volume – the LV volume at zero diastolic pressure [S-D][2], calculated as a fraction k_{HL} of V_{HL}.	–
005	$FS_{threshold}(0) = mass_{volume}(FS_{threshold0}, m)$	The Frank-Starling law threshold $FS_{threshold}$ is the normal average value $FS_{threshold0}$ normalized to the body weight *m*.	Proshin[3]<br>MATLAB
006	$SV_{\max}(0) = mass_{volume}(SV_{\max 0}, m)$	Theoretical maximum of the VL stroke volume $SV_{\max}$ is the average value $SV_{\max 0}$ normalized to the body weight *m*.	Proshin<br>MATLAB
007	If:<br>$Cycle_{Time} \geq Cycle_{Length}$<br>Then:<br>1. $Cycle_{Length} = \frac{1}{H}$<br>2. $Cycle_{Time} = 0$<br>3. $V_{HL\_KD} = V_{HL}$<br>4. $SV = K_L \cdot SV_{\max} \cdot [sigm(0.03 \cdot (V_{HL} - FS_{threshold} - 80)) -$<br>$- sigm(0.03 \cdot (V_{HL} - FS_{threshold} - 260))]$	Transition "diastole – systole". A discrete event determined by the instantaneous change of the LV parameters at the beginning of the cardiac cycle. The event is triggered when a current cycle time $Cycle_{Time}$ reaches a cycle length $Cycle_{Length}$.<br>1. $Cycle_{Length}$ – a value inversely proportional to the value of the neurohumoral factor *H*.<br>2. Starting a new cycle corresponds to the zeroing $Cycle_{Time}$.<br>3. V_{HL_KD} – the LV blood volume at the end of diastole.<br>4. The LV stroke volume *SV* is defined as the product of the inotropic factor K_L, theoretical maximum $SV_{\max}$ and value of a bell-shaped	Proshin<br>MATLAB

[1] Formulas for calculating starting values of variables are used to reconcile them in the case of changing the initial parameter values, for example, during generation of a virtual patient. When we consider the model in equilibrium, these formulas are not taken into account.
[2] Here and below, the reference "S-D" means the educational resource: http://www.samara-dialog.ru/help/eng/help.htm.
[3] Mention of MATLAB hereinafter means that the detailed formula is not available in open sources and is taken from the Proshin and Solodyannikov model implementation in MATLAB obtained in correspondence with the authors.

	5. $V_{HL\_KS} = V_{HL} - K_L \cdot SV_{\max} \cdot \left[sigm\left(0.03 \cdot \left(V_{HL} - FS_{threshold} - 80\right)\right) - \right.$ $\left. - sigm\left(0.03 \cdot \left(V_{HL} - FS_{threshold} - 260\right)\right)\right]$<br>6. $Systole_{Length\_L\_Exp} = \frac{0.25}{H} + 0.2 \cdot \left(1 - K_L\right)$<br>7. $Systole_{Length\_L} = \frac{0.25}{H} + 0.2 \cdot \left(1 - K_L\right)$<br>8. $Systole_L = 1$<br>9. $P_D = P_{AL}$	function, which is equal to the difference of two logistic functions, depending on $V_{HL} = V_{HL\_KD}$ and threshold of the Frank-Starling law $FS_{threshold}$.<br>5. End systolic volume of the LV V_{HL_KS} is equal to the difference between $V_{HL} = V_{HL\_KD}$ and *SV*.<br>6. $Systole_{Length\_L\_Exp}$ – the nominal duration of the LV systole.<br>7. $Systole_{Length\_L}$ – the actual duration of the LV systole. It changes value at the transition "LV systole – LV diastole" resulting in a possible mismatch between $Systole_{Length\_L\_Exp}$ and $Systole_{Length\_L}$.<br>8. $Systole_L$ – indicator of the actual LV systole equals 1 when $Systole_{Length\_L\_Exp}$ and $Systole_{Length\_L}$ are matched.<br>9. Diastolic pressure P_D is the systemic arterial pressure P_{AL} at the end of diastole.	
008	If: $Systole = 1$<br>Then: $F_{HLAL\_p} = F_{HLAL}$	Systole start. Discrete event triggered after the transition "diastole – systole" (*Systole* = 1). At this point, blood flow through the aortic valve F_{HLAL} reaches its peak F_{HLAL_p}.	–
009	If:<br>$V_{HL} < V_{HL\_KS}$ and $Systole_L = 1$,<br>Then:<br>1. $Systole_L = 0$<br>2. $Systole_{Length\_L} = Cycle_{Time}$<br>3. $P_S = P_{AL}$	Transition "LV systole – LV diastole". A discrete event set by the instantaneous change of the LV parameters at the moment of switching the LV from systole to diastole. It fires when the LV volume V_{HL} reaches the LV end-systolic volume V_{HL_KS} in condition: the LV is in a systolic state.<br>1. $Systole_L = 0$, that is, the LV is in the state of diastole.<br>2. Actual duration of the LV systole $Systole_{Length\_L}$ corresponds to the transition time from diastole to systole.<br>3. P_S – blood pressure in the systemic arteries P_{AL} at the end of systole.	Proshin MATLAB
010	If:<br>$P_{VR} > P_{HL}$<br>Then:<br>$F_{VRHL\_ep} = F_{VRHL}$	Mitral valve opening. A discrete event triggered at the moment of the LV transition to diastole, when the pressure in pulmonary veins P_{VR} becomes greater than the pressure in the LV P_{HL}. In normal subjects, the LV filling rate reaches its highest value (early peak) F_{VRHL_ep} immediately after valve opening [Caudron].	–

011	$P_{HL\_D} = mass_{elasticity}\left(G_{HL}, m\right)\cdot\left(V_{HL} - \omega_{HL}\right)$	Diastolic pressure in the LV P_{HL_D} depends on the blood volume V_{HL}, unstressed volume ω_{HL} and the LV wall elasticity G_{HL} (characterizing stiffness of the LV wall in the phase of its relaxation). The allometric scaling formula for G_{HL} is taken from the MATLAB implementation of the model by Proshin and Solodyannikov.	Ottesen[4] Suga
012	$Systole_{L\_Exp} = \begin{cases} 0, & Cycle_{Time} \geq Systole_{Length\_L\_Exp} \\ 1, & else \end{cases}$	Nominal LV systole indicator.	Proshin MATLAB
013	$DTS_L = Systole_L - Systole_{L\_Exp}$	LV systolic mismatch.	Proshin MATLAB
014	$\frac{dCycle_{Time}}{dt} = 1$	Mapping time of the current cardiac cycle $Cycle_{Time}$ to a model time of the LV module.	–
015	$\frac{dP_{HL\_S}}{dt} = A_5 \cdot DTS_L$	LV systolic pressure P_{HL_S} depending on a magnitude of mismatch DTS_L and sensitivity coefficient A_5.	Proshin MATLAB
016	$P_{HL} = \left(Systole - Systole_L\right)\cdot P_{AL} + \left(1 - Systole\right)\cdot P_{HL\_D} + P_{HL\_S}\cdot Systole_L$	LV blood pressure P_{HL}:<br>$P_{HL} = P_{HL\_S}$, if the LV is in the state of systole;<br>$P_{HL} = P_{AL}$, if the LV is in the state of diastole, and the RV is in the state of systole;<br>$P_{HL} = P_{HL\_D}$, if both the LV and the RV are in the state of diastole.	Proshin MATLAB
017	$Y_{VRHL} = mass_{conductivity}\left(Y_{VRHL0} + A_{16}\cdot P_{VR}, m\right)$	Conductivity of the mitral valve and pulmonary veins Y_{VRHL} is the inverse of resistance to blood flow in the specified section of the bloodstream. The value of Y_{VRHL} is calculated according to the allometric law depending on the body weight *m*. Y_{VRHL0} – basic conductivity.<br>P_{VR} – pulmonary vein and left atrial pressure. A_{16} – constant.	Proshin MATLAB

[4] In the work [Ottesen], when describing the model of a pumping heart, the unified formula for relation between left ventricular cavity pressure p_{lv} and ventricular volume V_{lv} is given by: $p_{lv} = E_{lv}(t)\cdot\left(V_{lv} - V_{d,lv}\right)$, where $V_{d,lv}$ is the LV volume at $p_{lv} = 0$, and the elasticity function $E_{lv}(t)$ remains constant and takes the minimum value during diastolic filling of the LV. In the MATLAB implementation of the model by Proshin and Solodyannikov, the value P_{HL_D} is calculated taking into account residual pressure in the LV P_{HL0} as a quadratic function: $P_{HL\_D} = P_{HL0} + mass_{elasticity}\left(G_{HL}, m\right)\cdot\left(V_{HL} - \omega_{HL}\right)^2$. In addition, exponential and logarithmic functions [Zhang, 2008], cubic and power functions [Burkhoff] can be used to model the variable P_{HL_D} corresponding to the end-diastolic pressure-volume relationship (EDPVR).

018	$F_{VRHL} = valve\left(P_{VR}, P_{HL}, K_{VRHL}, Y_{VRHL}\right)$	Amount of blood flow through the mitral valve F_{VRHL} is characterized by the difference between pressure in the pulmonary vein and the left atrium P_{VR} and pressure in the LV P_{HL}.<br>Y_{VRHL} – conductivity of the mitral valve and pulmonary veins.<br>K_{VRHL} – regurgitation coefficient describes the mitral valve closing function failure that leads to the reverse blood flow from the LV into the left atrium and pulmonary vein in the systole phase in conditions of the valve defect.	S-D
019	$F_{HLAL} = valve\left(P_{HL}, P_{AL}, K_{HLAL}, mass_{conductivity}\left(Y_{HLAL}, m\right)\right)$	Amount of blood flow through the aortic valve F_{HLAL} is characterized by the difference between pressure in the LV P_{HL} and pressure in the upper part of the arterial bed (the aorta and large arterial vessels extending from it) P_{AL}.<br>Y_{HLAL} – conductivity of the aortic valve and systemic arteries taken with the allometric scaling relative to the body weight *m*.<br>K_{HLAL} – regurgitation coefficient describes the aortic valve closing function failure that leads to the reverse blood flow from the aorta to the LV in the diastole phase in conditions of the valve defect.	S-D
020	$LA_{PULSE} = atrium_{pulse}\left(Cycle_{Length}, Systole_{Length\_L}, P_{AL}, Cycle_{Time}\right)$	Left atrial pulse wave depends on the cycle length $Cycle_{Length}$, actual duration of the LV systole $Systole_{Length\_L}$, blood pressure P_{AL}, and current cycle time $Cycle_{Time}$.	Proshin MATLAB
021	If:<br>$Cycle_{Time} > Cycle_{Length} - 0.2 \cdot \left(Cycle_{Length} - Systole_{Length\_L}\right)$<br>Then:<br>$F_{VRHL\_ap} = F_{VRHL}$	A discrete event that defines the active peak F_{VRHL_ap} in blood flow through the mitral valve F_{VRHL}. This peak is achieved with left atrial contraction [Caudron] at the time when LA_{PULSE} becomes positive.	–
022	$MAP = \frac{P_S + 2 \cdot P_D}{3}$	Mean arterial pressure *MAP* depends on systolic blood pressure P_S and diastolic blood pressure P_D.	Moran
023	$Heart_{Rate} = \frac{60}{Cycle_{Length}}$	Length of the cardiac cycle $Cycle_{Length}$, by definition, corresponds to the time of one heartbeat. Heart rate $Heart_{Rate}$ is the number of heart beats per minute.	–
024	$CO = \frac{SV \cdot Heart_{Rate}}{1000}$	Cardiac output *CO* (l/min) is equal to the product of stroke volume *SV* (ml) and $Heart_{Rate}$ (bpm).	Cattermole

025	$TPR = MAP/CO$	Total peripheral resistance *TPR* is the ratio of mean arterial pressure *MAP* to cardiac output *CO*.	Cattermole Daly
026	$EF = \frac{SV}{V_{HL\_KD}} \cdot 100$	Ejection fraction (*EF*) is stroke volume *SV* divided by the LV end-diastolic volume V_{HL_KD} and multiplied by 100%.	Maceira 2006a
027	$V_{HL\_KS}(0) = V_{HL\_KD} - K_L \cdot SV_{\max} \cdot \Big[sigm\Big(0.03 \cdot \big(V_{HL\_KD} -$ $- FS_{threshold} - 80\big)\Big) - sigm\Big(0.03 \cdot \big(V_{HL\_KD} - FS_{threshold} - 260\big)\Big)\Big]$ $Systole_{Length\_L\_Exp}(0) = 0.25 \cdot Cycle_{Length} + 0.2 \cdot (1 - K_L)$ $Systole_{Length\_L}(0) = 0.25 \cdot Cycle_{Length} + 0.2 \cdot (1 - K_L)$	Initialization of parameters according to the implementation of the model by Proshin and Solodyannikov in MATLAB received from the authors. See equation group 007 for explanation.	Proshin MATLAB
**Systemic arteries**			
028	$\frac{dV_{AL}}{dt} = F_{HLAL} - F_{ALVL}$	Change in blood volume in arteries of the systemic circulation is the difference between input (F_{HLAL}) and output (F_{ALVL}) blood flow.	Proshin
029	$V_{AL}(0) = 0.13 \cdot V$	13% of the total blood volume *V* is contained in the systemic arteries.	Hall
030	$G_{AL} = mass_{elasticity}(G_{AL0} + A_9 \cdot H, m)$	Systemic arterial elastance G_{AL} is calculated according to the allometric law depending on the body weight *m*. G_{AL0} – basic (independent of nervous and hormonal influences) systemic arterial elastance. A_9 – degree of the G_{AL0} dependence on nerve and hormonal influences. *H* – neurohumoral factor.	Proshin MATLAB
031	$\omega_{AL} = \omega_{AL\_nom} - mass_{volume}(A_8, m) \cdot H$	Unstressed volume of the systemic circulation arteries ω_{AL} is a volume of blood in the arteries at pressure equal to atmospheric pressure [S-D]. ω_{AL_nom} – nominal unstressed volume of the arteries. A_8 – sympathetic sensitivity of the systemic arteries normalized to the body weight *m*. *H* – neurohumoral factor.	Proshin MATLAB
032	$\omega_{AL\_nom}(0) = k_{AL} \cdot V_{AL}$	ω_{AL_nom} – unstressed volume of the systemic arteries without nervous and hormonal influences calculated as a fraction k_{AL} of V_{AL}.	–
033	$P_{AL} = G_{AL} \cdot (V_{AL} - \omega_{AL})$	Arterial blood pressure P_{AL} depends on the systemic arterial elastance G_{AL}, blood volume V_{AL} and unstressed volume ω_{AL}.	Proshin Ottesen
034	$Vis = 1.23 \cdot \left(1 - \frac{Hct}{99}\right)^{-n}, \; n = 1.7 + 9.86 \cdot \exp(-0.0607 \cdot Hct)$	Blood viscosity *Vis* is calculated as the function of hematocrit (*Hct*) with the plasma viscosity of 1.23 cP.	Hund

035	$F_{ALVL} = \dfrac{Y_{ALVL} \cdot (P_{AL} - P_{VL})}{\dfrac{Vis}{Vis_{norm}} \cdot \psi_{AT1\_ALVL}}$	Amount of blood flow from arterial to venous bed of the systemic circulation depends on the conductivity of the systemic microvessels (arterioles, capillaries and venules) Y_{ALVL}, as well as the difference between pressures in the systemic arteries P_{AL} and veins P_{VL} [Proshin]: $$F_{ALVL} = Y_{ALVL} \cdot (P_{AL} - P_{VL}).$$ We modified this formula based on the following facts.<br>1. Value F_{ALVL} is inversely proportional to the normalized blood viscosity Vis/Vis_{norm} [Guyton].<br>2. Conductivity Y_{ALVL} is the inverse of microvessel resistance influenced by calcium ions and angiotensin II binding to their AT1 receptors in the vascular smooth muscle [Hughes] (mainly in the arteries [Consigny]). To take into account the vasodilating effect of antihypertensive drugs affecting the renin-angiotensin-aldosterone system (and, as a consequence, the concentration of AT1-bound angiotensin II), we determined the function ψ_{AT1_ALVL}.	Proshin<br>Guyton
036	$\psi_{AT1\_ALVL} = A_{AT1\_ALVL} + B_{AT1\_ALVL} \cdot AT1\_ANGII - \dfrac{C_{AT1\_ALVL}}{AT1\_ANGII}$	Effect of AT1-bound angiotensin II (*AT1_ANGII*) on Y_{ALVL} is modeled by analogy with the influence functions of *AT1_ANGII* on resistance of efferent/afferent arterioles, as well as interlobar, arcuate, and interlobular arteries in the model [Hallow, 2014]. We assume that a concentration of AT1-bound angiotensin II in the systemic arterioles has the same value as in the kidneys. A_{AT1_ALVL}, B_{AT1_ALVL} and C_{AT1_ALVL} are constant.	–
**Systemic microvessels**			
037	$Y_{ALVL} = mass_{conductivity}(Y_{ALVL0} - A_3 \cdot H + A_4 \cdot DO_2, m)$	Conductivity of the systemic microvessels (arterioles, capillaries and venules) Y_{ALVL}, the inverse of resistance to blood flow in the specified section of the bloodstream, is calculated according to the allometric law depending on the body weight *m*.<br>Y_{ALVL0} – basic conductivity.<br>A_3 – sympathetic sensitivity of the systemic circulation microvessels characterizes a degree of the Y_{ALVL} dependence on nervous and hormonal influences. *H* – neurohumoral factor.<br>A_4 – oxygen-deficient sensitivity of the systemic circulation microvessels characterizes a degree of the Y_{ALVL} dependence on the oxygen debt DO_2 [S-D].	Proshin<br>MATLAB

**Systemic veins**			
038	$V_{VL} = V - V_{AL} - V_{AR} - V_{HL} - V_{HR} - V_{VR}$	Volume of blood in the systemic veins V_{VL} is calculated by reducing the total circulating blood volume V by the volumes of all other compartments representing the cardiovascular system.	Proshin
039	$G_{VL} = mass_{elasticity}\left(G_{VL0} + A_{11} \cdot H, m\right)$	Systemic vein elastance G_{VL} is calculated by the allometric function taking the body weight *m*.<br>G_{VL0} – basic elasticity of the systemic vein walls independent of nervous and hormonal influences.<br>A_{11} – venous tone, the G_{VL} dependence on the indicated influences. *H* – neurohumoral factor.	Proshin<br>MATLAB
040	$\omega_{VL}(0) = k_{VL} \cdot V_{VL}$	Unstressed volume of the systemic circulation veins ω_{VL} is calculated as a fraction k_{VL} of V_{VL}.	–
041	$P_{VL} = P_0 + RA_{PULSE} + G_{VL} \cdot \left(V_{VL} - \omega_{VL}\right)$	Blood pressure in the systemic circulation veins P_{VL} is the sum of the base pressure P_0, pulse wave of the right atrium RA_{PULSE}, as well as the product of the systemic vein elastance G_{VL} and the difference between the blood volume V_{VL} and unstressed volume ω_{VL}.	Proshin<br>MATLAB
**Right ventricle (RV)**			
042	$\frac{dV_{HR}}{dt} = F_{VLHR} - F_{HRAR}$	Change in blood volume in the RV due to the difference between the input blood flow F_{VLHR} and the output blood flow F_{HRAR}.	Proshin
043	$V_{HR}(0) = 0.03 \cdot V$	Starting value of blood volume in the RV is 3% of the total blood volume *V*, because the systemic circulation – 84% [Hall], the pulmonary circulation – 10% [Gazioglu], and values $V_{HL}(0)$ and $V_{HR}(0)$ are assumed to be equal.	Hall<br>Gazioglu
044	$K_R = K_{R0} + 0.25 \cdot nH \cdot sigm\left(20 \cdot \left(K_{R0} - 0.4\right)\right) - 0.25 \cdot nH \cdot sigm\left(20 \cdot \left(K_{R0} - 0.7\right)\right)$	Inotropic factor of the RV. Numerical characteristic of the myocardial inotropism (contractile ability). K_{R0} – inotropic status of the RV.<br>nH – myocardial sympathetic inotropic sensitivity.	Proshin
045	$\omega_{HR}(0) = k_{HR} \cdot V_{HR}$	Unstressed RV volume ω_{HR} is the RV volume at zero diastolic pressure [S-D], calculated as a fraction k_{HR} of V_{HR}.	–

046	If:<br>$Cycle_{Time} \geq Cycle_{Length}$<br><br>Then:<br>1. $Cycle_{Time} = 0$<br>2. $V_{HR\_KD} = V_{HR}$<br>3. $V_{HR\_KS} = V_{HR} - K_R \cdot SV_{\max} \cdot \left[sigm\left(0.03 \cdot \left(V_{HR} - FS_{threshold} - 80\right)\right) - sigm\left(0.03 \cdot \left(V_{HR} - FS_{threshold} - 260\right)\right)\right]$<br>4. $Systole_{Length\_R\_Exp} = \frac{0.25}{H} + 0.2 \cdot (1 - K_R)$<br>5. $Systole_{Length\_R} = \frac{0.25}{H} + 0.2 \cdot (1 - K_R)$<br>6. $Systole_R = 1$<br>7. $P_{AR\_D} = P_{AR}$	Transition "diastole – systole" is the instantaneous change of the RV parameters at the beginning of the cardiac cycle (a current cycle time $Cycle_{Time}$ reaches a cycle duration $Cycle_{Length}$).<br>1. Starting a new cycle corresponds to the zeroing $Cycle_{Time}$.<br>2. End diastolic volume of the RV V_{HR_KD} is equal to the RV blood volume at the end of diastole.<br>3. End systolic volume of the RV V_{HR_KS} is equal to the difference between $V_{HR} = V_{HR\_KD}$ and the stroke volume of the RV, calculated as the product of K_R, theoretical maximum $SV_{\max}$ and the difference of two logistic functions, depending on V_{HR} and $FS_{threshold}$ (threshold of the Frank-Starling law).<br>4. $Systole_{Length\_R\_Exp}$ – the nominal duration of the RV systole.<br>5. $Systole_{Length\_R}$ – the actual duration of the RV systole. It changes a value at the transition "RV systole – RV diastole", after which a mismatch may occur between $Systole_{Length\_R\_Exp}$ and $Systole_{Length\_R}$.<br>6. $Systole_R$ – indicator of the actual RV systole. It equals 1 when $Systole_{Length\_R\_Exp}$ and $Systole_{Length\_R}$ are matched.<br>7. P_{AR_D} is diastolic pressure in the pulmonary arteries.	Proshin MATLAB
047	If: $Systole = 1$<br>Then: $F_{HRAR\_p} = F_{HRAR}$	Systole start. A discrete event triggered after the transition "diastole – systole" (*Systole* = 1). At this time point, blood flow through the pulmonary valve F_{HRAR} achieves a peak value F_{HRAR_p}.	–
048	If:<br>$V_{HR} < V_{HR\_KS}$ and $Systole_R = 1$<br>Then:<br>1. $Systole_R = 0$<br>2. $Systole_{Length\_R} = Cycle_{Time}$<br>3. $P_{AR\_S} = P_{AR}$	Transition "RV systole – RV diastole". A discrete event characterized by the instantaneous change of the RV parameters at time of the diastole onset; it is triggered when the RV volume V_{HR} reaches the RV end-systolic volume V_{HR_KS} provided that the RV is in the state of systole.<br>1. $Systole_R = 0$, i.e. the RV is in the state of diastole.<br>2. Actual duration of the RV systole $Systole_{Length\_R}$ corresponds to the transition time from diastole to systole.<br>3. P_{AR_S} – systolic pressure in the pulmonary arteries.	Proshin MATLAB

049	If:<br>$P_{VL} > P_{HR}$<br>Then:<br>$F_{VLHR\_ep} = F_{VLHR}$	Opening of the tricuspid valve. A discrete event triggered as the RV enters the diastole state, when pressure in the systemic veins P_{VL} becomes greater than pressure in the RV P_{HR}. F_{VLHR_ep} is the early peak of the RV filling [van Straten].	–
050	$P_{HR\_D} = mass_{elasticity}\left(G_{HR}, m\right) \cdot \left(V_{HR} - \omega_{HR}\right)$	Diastolic pressure in the RV P_{HR_D} depends on the blood volume V_{HR}, unstressed volume ω_{HR} and the RV wall elasticity G_{HR} (characterizing stiffness of the RV wall in the phase of its relaxation) taking into account the allometric scaling [Proshin, MATLAB].	Ottesen
051	$Systole_{R\_Exp} = \begin{cases} 0, & Cycle_{Time} \ge Systole_{Length\_R\_Exp} \\ 1, & else \end{cases}$	Nominal RV systole indicator.	Proshin MATLAB
052	$DTS_R = Systole_R - Systole_{R\_Exp}$	RV systolic mismatch.	Proshin MATLAB
053	$\frac{dCycle_{Time}}{dt} = 1$	Mapping time of the current cardiac cycle $Cycle_{Time}$ to the model time of the RV module.	–
054	$\frac{dP_{HR\_S}}{dt} = A_{15} \cdot DTS_R$	Systolic pressure in the RV P_{HR_S} depending on a magnitude of mismatch DTS_R and coefficient of sensitivity A_{15}.	Proshin MATLAB
055	$P_{HR} = Systole_R \cdot P_{HR\_S} + P_{AR} \cdot \left(Systole - Systole_R\right) +$<br>$+ \left(1 - Systole\right) \cdot P_{HR\_D}$	RV pressure P_{HR} is calculated by the following way:<br>$P_{HR} = P_{HR\_S}$, if the RV is in the state of systole;<br>$P_{HR} = P_{AR}$, if the RV is in the state of diastole, and the LV is in the state of systole;<br>$P_{HR} = P_{HR\_D}$, if both the RV and the LV are in the state of diastole.	Proshin MATLAB
056	$Y_{VLHR} = mass_{conductivity}\left(Y_{VLHR0} + P_{VL} \cdot A_6 + RO_{20} \cdot A_7 - P_{HR\_S} \cdot A_{10}, m\right)$	Conductivity of the tricuspid valve and systemic veins Y_{VLHR} is the inverse of resistance to blood flow in the specified area of the bloodstream. It is calculated taking into account the allometric scaling relative to the body weight *m*. Y_{VLHR0} – basic conductivity,<br>P_{VL} – pressure in the inferior vena cava and the right atrium,<br>RO_{20} – average normal value of oxygen demand,<br>P_{HR_S} – systolic pressure in the RV, A_6, A_7, A_{10} – constants.	Proshin MATLAB

057	$F_{VLHR} = valve\left(P_{VL}, P_{HR}, K_{VLHR}, Y_{VLHR}\right)$	Amount of blood flow through the tricuspid valve F_{VLHR} is characterized by the difference between pressure in the inferior vena cava and the right atrium P_{VL} and pressure in the RV P_{HR}.<br>Y_{VLHR} – conductivity of the tricuspid valve and systemic veins.<br>K_{VLHR} – regurgitation coefficient describes the tricuspid valve closing function failure that leads to the reverse blood flow from the RV to the right atrium and vena cava in the systole phase in conditions of the valve defect.	S-D
058	$F_{HRAR} = valve\left(P_{HR}, P_{AR}, K_{HRAR},\ mass_{conductivity}\left(Y_{HRAR}, m\right)\right)$	Amount of blood flow through the pulmonary valve and pulmonary arteries F_{HRAR} is characterized by the difference between pressure in the RV P_{HR} and pressure P_{AR} in the upper part of the pulmonary artery and large arterial vessels extending from it.<br>Y_{HRAR} – conductivity of the pulmonary valve and pulmonary arteries taking into account the allometric scaling relative to the body weight *m*.<br>K_{HRAR} – regurgitation coefficient describing the pulmonary valve closing function failure that leads to the reverse blood flow from the pulmonary artery to the RV in the diastole phase in conditions of the valve defect.	S-D
059	$RA_{PULSE} = atrium_{pulse}\left(Cycle_{Length}, Systole_{Length\_R}, P_{AL}, Cycle_{Time}\right)$	Right atrium pulse wave depends on the cycle duration $Cycle_{Length}$, actual duration of the RV systole $Systole_{Length\_R}$, pressure in arteries of the systemic circulation P_{AL} and the current time of the cardiac cycle $Cycle_{Time}$.	Proshin MATLAB
060	If:<br>$Cycle_{Time} > Cycle_{Length} - 0.2\cdot\left(Cycle_{Length} - Systole_{Length\_R}\right)$<br>Then:<br>$F_{VLHR\_ap} = F_{VLHR}$	A discrete event that defines the active peak F_{VLHR_ap} in blood flow through the tricuspid valve F_{VLHR}. This peak is achieved with the right atrial contraction [van Straten] at the time when the value RA_{PULSE} becomes positive.	–
061	$V_{HR\_KS}\left(0\right) = V_{HR\_KD} - K_R \cdot SV_{\max}\cdot\left[sigm\left(0.03\cdot\left(V_{HR\_KD} -\right.\right.\right.$<br>$\left.\left.\left.-FS_{threshold} - 80\right)\right) - sigm\left(0.03\cdot\left(V_{HR\_KD} - FS_{threshold} - 260\right)\right)\right]$<br>$Systole_{Length\_R\_Exp}\left(0\right) = 0.25\cdot Cycle_{Length} + 0.2\cdot\left(1 - K_R\right)$<br>$Systole_{Length\_R}\left(0\right) = 0.25\cdot Cycle_{Length} + 0.2\cdot\left(1 - K_R\right)$	Initialization of parameters, according to the implementation of the model by Proshin and Solodyannikov in MATLAB, received from the authors. See equation group 045 for explanation.	Proshin MATLAB

Pulmonary arteries			
062	$\frac{dV_{AR}}{dt} = F_{HRAR} - F_{ARVR}$	Blood volume change in the pulmonary circulation arteries due to the difference between the input blood flow F_{HRAR} and the output blood flow F_{ARVR}.	Proshin
063	$V_{AR}(0) = 0.035 \cdot V$	Starting value of blood volume in the pulmonary arteries is 3.5% of the total blood volume *V*, because the pulmonary circulation is 10% of *V*, and the pulmonary arterial volume is 35% of the pulmonary vessels.	Gazioglu
064	$G_{AR} = mass_{elasticity}(G_{AR0} + A_{19} \cdot H, m)$	Pulmonary arterial elastance G_{AR} is calculated by the allometric function taking the body weight *m*.<br>G_{AR0} – basic pulmonary arterial elastance.<br>A_{19} – pulmonary arterial tone, the degree of the G_{AR} dependence on nervous and hormonal influences.<br>*H* – neurohumoral factor.	Proshin<br>MATLAB
065	$\omega_{AR} = \omega_{AR\_nom} - mass_{volume}(A_{18}, m) \cdot H$	Unstressed volume of the pulmonary circulation arteries ω_{AR} is the volume of blood in these arteries at pressure equal to the atmospheric pressure [S-D].<br>ω_{AR_nom} – nominal unstressed volume.<br>A_{18} – sympathetic sensitivity of the pulmonary circulation arteries.<br>*H* – neurohumoral factor.	Proshin<br>MATLAB
066	$\omega_{AR\_nom}(0) = k_{AR} \cdot V_{AR}$	Unstressed volume of the pulmonary arteries without hormonal and nervous influences ω_{AR_nom} is calculated as a fraction k_{AR} of V_{AR}.	–
067	$P_{AR} = G_{AR} \cdot (V_{AR} - \omega_{AR})$	Pressure in the pulmonary arteries P_{AR} depends on the pulmonary arterial elastance G_{AR}, blood volume V_{AR}, and unstressed volume ω_{AR}.	Proshin<br>Ottesen
068	$F_{ARVR} = \frac{Y_{ARVR}}{Vis/Vis_{norm}} \cdot (P_{AR} - P_{VR})$	Blood flow through the pulmonary microvessels F_{ARVR} depends on their conductivity Y_{ARVR} and the difference between pressures in the pulmonary arteries P_{AR} and veins P_{VR} [Proshin]:<br>$F_{ARVR} = Y_{ARVR} \cdot (P_{AR} - P_{VR})$.<br>We modified this formula so that the value of F_{ARVR} should be inversely proportional to the normalized blood viscosity Vis/Vis_{norm} [Guyton].	Proshin<br>Guyton

**Pulmonary microvessels**			
069	$Y_{ARVR} = mass_{conductivity}\left(Y_{ARVR0} - A_{13} \cdot H + A_{14} \cdot DO_2, m\right)$	Conductivity of the pulmonary microvessels (venules, capillaries and arterioles) Y_{ARVR}, the inverse of resistance to blood flow in the indicated section of the bloodstream, is calculated according to the allometric law depending on the body weight *m*.<br>Y_{ARVR0} – basic conductivity.<br>A_{13} – sympathetic sensitivity of the pulmonary microvessels characterizes dependence of Y_{ARVR} on nervous and hormonal influences.<br>*H* – neurohumoral factor.<br>A_{14} – pulmonary microvessels sensitivity to the oxygen debt DO_2 [S-D].	Proshin MATLAB
**Pulmonary veins**			
070	$\frac{dV_{VR}}{dt} = F_{ARVR} - F_{VRHL}$	Blood volume change in the pulmonary circulation veins due to the difference between the input blood flow F_{ARVR} and the output blood flow F_{VRHL}.	Proshin
071	$V_{VR}\left(0\right) = 0.065 \cdot V$	Starting value of blood volume in the pulmonary veins is 6.5% of the total blood volume *V*, because the pulmonary circulation is 10% of *V*, pulmonary veins – 45%, and capillaries – 20% of pulmonary vessels [Gazioglu]. In the model by Proshin and Solodyannikov, the pulmonary veins and capillaries are included in one compartment of the circulatory system.	Gazioglu Proshin
072	$G_{VR} = mass_{elasticity}\left(G_{VR0} + A_{11} \cdot H, m\right)$	Pulmonary vein elastance G_{VR} is calculated by the allometric function taking the body mass *m*.<br>G_{VR0} – basic elasticity of the pulmonary vein walls independent of nervous and hormonal influences.<br>A_{11} – venous tone, the G_{VR} dependence on the indicated influences.<br>*H* – neurohumoral factor.	Proshin MATLAB
073	$\omega_{VR}\left(0\right) = k_{VR} \cdot V_{VR}$	Unstressed volume of the pulmonary circulation veins ω_{VR} is calculated as a fraction k_{VR} of V_{VR}.	–
074	$P_{VR} = P_0 + LA_{PULSE} + G_{VR} \cdot \left(V_{VR} - \omega_{VR}\right)$	Blood pressure in the pulmonary circulation veins P_{VR} is the sum of the base pressure P_0, left atrium pulse wave LA_{PULSE}, and the product of the pulmonary vein elastance G_{VR} and the difference between the blood volume V_{VR} and unstressed volume ω_{VR}.	Proshin MATLAB

**Tissue metabolism**			
075	$gO_2 = F_{ALVL} \cdot \min\left(AO_2 - VO_2, 1\right)$	Amount of oxygen consumption gO_2 in tissues is proportional to blood flow through the tissues F_{ALVL} and the arteriovenous oxygen difference $AO_2 - VO_2$.	Proshin MATLAB
076	$AO_2 = \frac{He \cdot C_H \cdot SpO_2}{1000}$	Arterial oxygen content AO_2 is calculated as the product of the total amount of hemoglobin *He* (g/l), oxygen capacity of hemoglobin C_H (mg/ml) and arterial oxygen saturation SpO_2, which characterizes a percentage of hemoglobin saturated with oxygen.	S-D
077	$\frac{dDO_2}{dt} = \mathrm{sgn}\left(DO_2, A_2 \cdot \left(RO_2 - gO_2\right)\right)$	Rate of change of the accumulated amount of unmet oxygen requirement (oxygen debt) DO_2 is proportional to the difference between the oxygen demand RO_2 and oxygen consumption gO_2. Oxygen debt decreases with $RO_2 < gO_2$, and increases with $RO_2 > gO_2$. Parameter A_2 is the organism functional status. It describes the rate of change of the weariness factor under the change of physical activity, with advent of the non-zero difference between RO_2 and gO_2. The parameter does not effect on periodical modes in the absence of changes in physical activity.	Proshin MATLAB
078	$\frac{dVO_2}{dt} = A_1 \cdot \left(gO_2 - RO_2\right)$	Rate of change of the venous oxygen content VO_2 is proportional to the difference between the oxygen consumption gO_2 and oxygen demand RO_2. A_1 is the total metabolic intensity describing the rate of transient processes under the change of physical activity, i.e. with advent of the non-zero difference between RO_2 and gO_2.	Proshin
079	$RO_2\left(0\right) = mass_{volume}\left(RO_{20}, m\right)$	Oxygen demand is calculated according to the allometric law at the initial moment of time and depends on the average normal value RO_{20} and the body weight *m*.	Proshin MATLAB
**Neurohumoral control**			
080	$nB = \min\left(r_{minus}\left(0.07, 30, P_{AL}, 50 \cdot \psi_{AT1\_Baro}\right), 1\right)$	In the model by Proshin and Solodyannikov, the baroreceptor activity is determined by the function $nB = \min\left(r_{minus}\left(0.07, 30, P_{AL}, 50\right), 1\right)$, where P_{AL} is arterial pressure in the systemic circulation, and $P_0 = 50$ mmHg is the baroreceptor sensitivity threshold, i.e. the lower bound for P_{AL}, at which *nB* = 0. When modeling the pharmacological action of drugs affecting the concentration of AT1-bound angiotensin II (*AT1_ANGII*), we took into account the fact that this hormone, interacting with AT1	Proshin MATLAB

		receptors, promotes an increase in blood pressure without a decrease in heart rate through raising the P_0 threshold [Reid]. We achieved such dynamics using a coefficient ψ_{AT1_Baro} proportional to *AT1_ANGII*. Together with affect on the stress receptor activity function, this decision allowed to simulate the therapeutic decrease in the P_{AL} value without changing the heart rate, which, for example, characterizes losartan [Porthan], [Nedogoda].	
081	$\psi_{AT1\_Baro} = sl_{baro} \cdot \frac{AT1\_ANGII}{AT1\_ANGII_{norm}} + 1 - sl_{baro}$	We assumed the linear function with the slope $0 < sl_{baro} < 1$ as the influence of the normalized AT1-bound angiotensin II level $(AT1\_ANGII / AT1\_ANGII_{norm})$ on the threshold of baroreceptor sensitivity.	–
082	$nS = \min\left(r_{plus}\left(10, 50, st \cdot \psi_{AT1\_Stress}, 0\right), 1\right)$	In the Proshin and Solodyannikov model, activity of the stress receptors is determined by the function $nS = \min\left(r_{plus}\left(10, 50, st, 0\right), 1\right)$, where *st* is a stress factor describing the level of steroid hormones in the blood (adrenaline, norepinephrine) and taking nominal values from 0 (absolute rest) to 1 (absolute stress). We multiplied *st* by a coefficient ψ_{AT1_Stress} proportional to the concentration of AT1-bound angiotensin II (*AT1_ANGII*) based on the fact that this hormone, acting on AT1 receptors, increases the norepinephrine release from the atria [Brasch].	Proshin MATLAB
083	$\psi_{AT1\_Stress} = sl_{stress} \cdot \frac{AT1\_ANGII}{AT1\_ANGII_{norm}} + 1 - sl_{stress}$	We assumed the linear function with the slope $0 < sl_{stress} < 1$ as the effect of the normalized concentration of AT1-bound angiotensin II $(AT1\_ANGII / AT1\_ANGII_{norm})$ on the stress factor.	–
084	$nV = \min\left(r_{minus}\left(30, 30, VO_2, 0\right), 1\right)$	Respiratory receptor activity function depending on the venous oxygen content VO_2.	Proshin MATLAB
085	$nD = \min\left(r_{plus}\left(0.1, 10, DO_2, 0\right), 1\right)$	Weariness receptor activity function depending on the oxygen debt DO_2.	Proshin MATLAB
086	$nSum = Heart_{Baro} \cdot nB + Heart_{Stress} \cdot nS + Heart_{Oxygen} \cdot nD + Heart_{VO2} \cdot nV + Heart_{Base}$	Cardiac center activity function *nSum* is the sum signal of activities of the stress (*nS*), weariness (*nD*), and respiratory (*nV*) receptors, as well as the baroreceptors (*nB*).<br>$Heart_{Baro}$ is baroreceptor sensitivity of the cardiac center characterizing the dependence of nervous control actions on a value of arterial pressure.<br>$Heart_{Stress}$ is stress sensitivity of the cardiac center characterizing the dependence of nervous control actions on a value of the receptor stress signal.	Proshin

		$Heart_{Oxygen}$ is weariness sensitivity of the cardiac center characterizing the dependence of nervous control actions on the value of oxygen debt. A smaller value of the parameter corresponds to the lower dependence of heart rate on the level of oxygen starvation, i.e. the greater tolerance to the physical activity.<br>$Heart_{VO2}$ is respiratory sensitivity of the cardiac center characterizing the dependence of nervous control actions on the concentration of oxygen in the venous blood.<br>$Heart_{Base}$ is the basic activity of the cardiac center.	
087	$nH = \begin{cases} 0, & H < 0 \\ \dfrac{1-\exp(-3H)}{1+100\cdot\exp(-3H)}, & else \end{cases}$	Sigmoid function of the myocardial sympathetic inotropic sensitivity depending on the neurohumoral factor *H*.	Proshin MATLAB
088	$\dfrac{dH}{dt} = A_{12}\cdot\left(3.2\cdot\min\left(r_{plus}\left(2,18,nSum,0\right),1\right)-H\right)$	Equation for calculating the neurohumoral factor *H* taking into account reactivity of the cardiac center A_{12}, which characterizes its adaptive ability and determines the signal rate through the axon of formal neuron.	Proshin MATLAB
**Coordination of LV and RV systolic time intervals**			
089	$Systole = \begin{cases} 0, & Systole_L = 0 \ and \ Systole_R = 0 \\ 1, & else \end{cases}$	*Systole* is an indicator of the total actual systole. It equals 0, if the actual LV and RV systole indicators are simultaneously equal to 0, and 1, otherwise.	Proshin MATLAB
**Nervous system**			
090	$RSNA = N_{rsna}\cdot\alpha_{map}\cdot\alpha_{rap}$	Renal sympathetic nerve activity *RSNA* depends on the normalized value of N_{rsna}.	Karaaslan 2005
091	$\alpha_{map} = 0.5+1.1\cdot\left(1+\exp\left(\dfrac{MAP-100}{15}\right)\right)^{-1}$	Effect of mean arterial pressure *MAP* on *RSNA*.	Karaaslan 2005
092	$\alpha_{rap} = 1-0.008\cdot P_{ra}$	Effect of right atrial pressure P_{ra} on *RSNA*.	Karaaslan 2005
093	$P_{ra} = 0.2787\cdot\exp\left(0.2281\cdot CO\right)$	Normalized right atrial pressure P_{ra} is calculated according to the Frank-Starling law as the function of cardiac output *CO*.	Karaaslan 2005

**Sodium**[5]			
094	$\Phi_{filsod} = GFR \cdot C_{sod}$	Amount of the sodium filtered from the glomerulus to the proximal tubule per unit time Φ_{filsod} is expressed as the product of the glomerular filtration rate (*GFR*) and sodium plasma concentration C_{sod}.	Karaaslan 2005
095	$\eta_{pt\_sodreab} = \min\left(1, n_{\eta\_pt} \cdot \gamma_{filsod} \cdot \gamma_{at} \cdot \gamma_{rsna}\right)$	Fractional proximal sodium reabsorption is defined as the normal value n_{η_pt} multiplied by functions of the various factors described below. Cannot exceed 100% of the filtered sodium.	Karaaslan 2005
096	$\gamma_{at} = 0.95 + \dfrac{0.12}{1 + \exp\left(2.6 - 1.8 \cdot \log AT1\_ANGII\right)}$	An increase in the level of AT1-bound angiotensin II (*AT1_ANGII*) results in the rise of fractional proximal sodium reabsorption.	Karaaslan 2005
097	$\gamma_{filsod} = 0.8 + \dfrac{0.3}{1 + 138^{-1} \cdot \exp\left(\Phi_{filsod} - 14\right)}$	An increase in the amount of the filtered sodium load Φ_{filsod} leads to the decrease in the fractional proximal sodium reabsorption.	Karaaslan 2005[6]
098	$\gamma_{rsna} = 0.5 + \dfrac{0.7}{1 + 2.18^{-1} \cdot \exp\left(1 - RSNA\right)}$	An increase in the renal sympathetic nerve activity *RSNA* results in the rising of the fractional proximal sodium reabsorption.	Karaaslan 2005[6]
099	$\Phi_{pt\_sodreab} = \Phi_{filsod} \cdot \eta_{pt\_sodreab}$	Absolute proximal tubular sodium reabsorption rate.	Karaaslan 2005
100	$\Phi_{md\_sod} = \Phi_{filsod} - \Phi_{pt\_sodreab}$	Macula densa sodium flow rate.	Karaaslan 2005
101	$\eta_{dt\_sodreab} = \min\left(1, n_{\varepsilon\_dt} \cdot \psi_{al}\right)$	Fractional distal tubule sodium reabsorption is defined as the normal value n_{ε_dt} multiplied by the function of aldosterone concentration influence ψ_{al}. Cannot exceed 100%.	Hallow 2014
102	$\Psi_{al} = 0.17 + 0.94 \cdot \left(1 + \exp\left(\dfrac{0.48 - 1.2 \cdot \log C_{al}}{0.88}\right)\right)^{-1}$	Effect of the aldosterone concentration C_{al} on the fractional distal tubule sodium reabsorption.	Karaaslan 2005
103	$\Phi_{dt\_sodreab} = \Phi_{md\_sod} \cdot \eta_{dt\_sodreab}$	Absolute distal tubule sodium reabsorption rate.	Karaaslan 2005
104	$\Phi_{dt\_sod} = \Phi_{md\_sod} - \Phi_{dt\_sodreab}$	Rate of the distal tubule sodium outflow.	Karaaslan 2005
105	$\eta_{cd\_sodreab} = \min\left(1, n_{\eta\_cd} \cdot \lambda_{dt} \cdot \lambda_{anp}\right)$	Fractional collecting duct sodium reabsorption is defined as the normal value n_{η_cd} multiplied by functions of the various factors described below. Cannot exceed 100%.	Karaaslan 2005

[5] The process of sodium transport and reabsorption along the nephron is given by [Palmer].
[6] The formula is presented taking into account correction of typos revealed during correspondence with authors of the model during its validation in BioUML.

106	$\lambda_{dt} = 0.82 + \dfrac{0.39}{1 + \exp\left(0.5 \cdot \left(\Phi_{dt\_sod} - 1.6\right)\right)}$	Effect of the distal tubule sodium outflow rate Φ_{dt_sod} on the fractional collecting duct sodium reabsorption.	Karaaslan 2005
107	$\lambda_{anp} = -\ 0.1 \cdot \dfrac{C_{anp}}{C_{anp\_norm}} + 1.1199$	Effect of the atriopeptin concentration C_{anp} on the fractional collecting duct sodium reabsorption.	Karaaslan 2005
108	$\Phi_{cd\_sodreab} = \Phi_{dt\_sod} \cdot \eta_{cd\_sodreab}$	Absolute collecting duct sodium reabsorption rate.	Karaaslan 2005
109	$\Phi_{u\_sod} = \Phi_{dt\_sod} - \Phi_{cd\_sodreab}$	Urine sodium excretion rate.	Karaaslan 2005
110	$\dfrac{dM_{sod}}{dt} = \Phi_{sodin} - \Phi_{u\_sod}$	Rate of the total exchangeable sodium (M_{sod}) formation is determined by the difference between the rate of sodium intake Φ_{sodin} and the rate of sodium excretion Φ_{u_sod}.	Hallow 2014
111	$C_{sod} = 1.03 \cdot \dfrac{\left(M_{sod} - 410\right)}{TBW} - 0.29 \cdot glucose + 78.44$	Plasma sodium concentration C_{sod} is defined depending on the total body water TBW and the plasma glucose concentration (taking into account the conversion factor from mmol/l to mg/dl [Bhagat]) by the formula [Nguyen] obtained on the basis of the Edelman equation [Edelman]: $$C_{sod} = 1.03 \cdot \frac{M_{sod} + M_K}{TBW} - 23.8 - \frac{1.6}{100} \cdot \left(18 \cdot glucose - 120\right),$$ where M_K is the total exchangeable potassium [Moore, 1967]: $M_K = 97.4 \cdot TBW - 410$.	Nguyen<br><br>Moore 1967
**Renin**			
112	$R_{sec} = \dfrac{\ln 2}{h_{renin}} \cdot PRC_{nom} \cdot v_{MD\_sod} \cdot v_{RSNA} \cdot v_{AT1\_ANGII}$	In the model [Hallow, 2014], the renin secretion rate is determined by the equation $R_{sec} = N_{rs} \cdot v_{MD\_sod} \cdot v_{RSNA} \cdot v_{AT1\_ANGII}$, where N_{rs} is the renin secretion equilibrium rate, and v_{MD_sod}, v_{RSNA}, v_{AT1_ANGII} represent influences of various factors on R_{sec} (see below). We took into account that $N_{rs} = \ln 2 / h_{renin} \cdot PRC_{nom}$ according to [Hallow, 2017a].	Hallow 2014<br><br>Hallow 2017a
113	$\nu_{MD\_sod} = exp\left(-\tau_{MD\_renin} \cdot \left(\Phi_{md\_sod} - \Phi_{md\_sod\_0}\right)\right)$	Influence of the macula densa sodium flow rate Φ_{md_sod} on the rate of renin secretion R_{sec} using the nominal value $\Phi_{md\_sod\_0}$. τ_{MD_renin} is a constant.	Hallow 2017a

114	$\nu_{RSNA} = 1.89 - \dfrac{2.056}{1.358 + \exp\left(RSNA - 0.8667\right)}$	Effect of the renal sympathetic nerve activity *RSNA* on the rate of renin secretion R_{sec}.	Karaaslan 2005
115	$\nu_{AT1\_ANGII} = \left(\dfrac{AT1\_ANGII_{norm}}{AT1\_ANGII}\right)^{slope_{AT1\_PRC}}$	Effect of AT1-bound angiotensin II (*AT1_ANGII*) on the rate of renin secretion R_{sec}. $AT1\_ANGII_{norm}$ is the normal level of *AT1_ANGII*. $slope_{AT1\_PRC}$ is a constant.	Hallow 2017a
116	$\dfrac{dPRC}{dt} = R_{sec} - \dfrac{\ln 2}{h_{renin}} \cdot PRC$	Plasma renin concentration (*PRC*) is described by the renin secretion rate R_{sec} and the renin clearance rate with the half-life of h_{renin}.	Hallow 2014
117	$PRC(0) = PRC_{nom}$	Initial *PRC* value is the nominal value PRC_{nom}.	–
**Angiotensin**			
118	$PRA = PRC \cdot X_{PRC\_PRA}$	Plasma renin activity (*PRA*).<br>X_{PRC_PRA} – equilibrium ratio of *PRA* to *PRC*.	Hallow 2014
119	$\dfrac{dANGI}{dt} = PRA - \left(c_{ACE} + c_{chym} + c_{nep}\right) \cdot ANGI - \dfrac{\ln 2}{h_{ANGI}} \cdot ANGI$	Rate of change in the concentration of angiotensin I (*ANGI*).<br>c_{ACE} and c_{chym} – the constant rates of *ANGI* conversion to angiotensin II by ACE and by chymases, respectively.<br>c_{nep} – the constant rate of *ANGI* conversion to angiotensin-(1-7) by neprilisin.<br>h_{ANGI} – the half-life of *ANGI*.	Hallow 2014
120	$\dfrac{dANGII}{dt} = \left(c_{ACE} + c_{chym}\right) \cdot ANGI - \left(c_{ACE2} + c_{ANGII\_ANGIV} + c_{AT1} + c_{AT2}\right) \cdot ANGII - \dfrac{\ln 2}{h_{ANGII}} \cdot ANGII$	Rate of change in the concentration of angiotensin II (*ANGII*).<br>c_{ACE2} – the constant rate of *ANGII* conversion to angiotensin-(1-7) by ACE2.<br>c_{ANGII_ANGIV} – the constant rate of *ANGII* conversion to angiotensin IV.<br>c_{AT1} and c_{AT2} – the constant rates of *ANGII* binding to AT1 and AT2 receptors, respectively.<br>h_{ANGII} – the half-life of *ANGII*.	Hallow 2014
121	$\dfrac{dANG17}{dt} = c_{nep} \cdot ANGI + c_{ACE2} \cdot ANGII - \dfrac{\ln 2}{h_{ANG17}} \cdot ANG17$	Rate of angiotensin-(1-7) (*ANG*17) production by the converting of *ANGI* or *ANGII*, and clearance with the half-life of h_{ANG17}.	Hallow 2014
122	$\dfrac{dANGIV}{dt} = c_{ANGII\_ANGIV} \cdot ANGII - \dfrac{\ln 2}{h_{ANGIV}} \cdot ANGIV$	Rate of angiotensin IV (*ANGIV*) production by the converting of *ANGII*, and clearance with the half-life of h_{ANGIV}.	Hallow 2014
123	$\dfrac{dAT1\_ANGII}{dt} = c_{AT1} \cdot ANGII - \dfrac{\ln 2}{h_{AT1}} \cdot AT1\_ANGII$	Rate of AT1-bound angiotensin II complex formation (*AT1_ANGII*) and its clearance with the half-life of h_{AT1}.	Hallow 2014

124	$\frac{dAT2\_ANGII}{dt} = c_{AT2} \cdot ANGII - \frac{\ln 2}{h_{AT2}} \cdot AT2\_ANGII$	Rate of AT2-bound angiotensin II complex formation (*AT2_ANGII*) and its clearance with the half-life of h_{AT2}.	Hallow 2014
**Aldosterone**			
125	$N_{als} = \xi_{k\_sod} \cdot \xi_{map} \cdot \xi_{at}$	Normalized aldosterone secretion rate N_{als} is calculated by the product of the factors representing different effects described below.<br>ξ_{map} is the effect of mean arterial pressure *MAP* on N_{als};<br>$\xi_{map} = 1$ (no effect) if *MAP* is above normal.	Karaaslan 2005
126	$\xi_{k\_sod} = 2^{C_K - 4.5}$	In the model [Karaaslan, 2005], the factor ξ_{k_sod} characterizes the effect of potassium (C_K) to sodium (C_{sod}) concentration ratio on N_{als}, and $$\xi_{k\_sod} = \max\left(0, \frac{1}{0.003525} \cdot \frac{C_K}{C_{sod}} - 9\right).$$ In the case $C_K \le 0.031725 \cdot C_{sod}$, we have $\xi_{k\_sod} = 0$. Thus, considering the physiological norms of 3.5 – 5.5 mEq/L for C_K [Rastegar] and 137 – 147 mEq/L for C_{sod} [Payne], we find that the values $3.5 < C_K < 4.3$ completely block aldosterone secretion. However, studies [Chen] and [Kojima] using bovine cells showed that extremely low potassium levels (2 mmol/L) actually reduce aldosterone production stimulated by angiotensin II, but does not stop it completely. In addition, a low-sodium diet does not directly affect the aldosterone secretion, but indirectly through activation of the renin-angiotensin-aldosterone system, upregulation of AT1 receptor levels, and hyperplasia of the zona glomerulosa [Bollag]. Therefore, we did not consider a value C_{sod} in the formula for ξ_{k_sod}. Since a rise of 1 mmol/L in serum potassium concentration doubles the aldosterone secretion [Pralong], [Bollag], we used a power of two as the function of the C_K effect on N_{als}. We normalized this function so that the average normal value $C_K = 4.5$ mEq/L had an effect equal to 1. Note that the values of ξ_{k_sod} are in agreement with the values used in the model of human physiology HumMod (version 1.6.1) [Pruett]: ξ_{k_sod} (3.0) = 0.3, ξ_{k_sod} (4.4) = 1.0, and ξ_{k_sod} (6.0) = 3.0.	–

127	$\xi_{at} = 0.4 + 2.4 \cdot \left(1 + \exp\left(2.82 - 1.5 \cdot \frac{\log AT1\_ANGII}{0.8}\right)\right)^{-1}$	Effect of AT1-bound angiotensin II (*AT1_ANGII*) on the rate of aldosterone secretion.	Karaaslan 2005
128	$\frac{dN_{al}}{dt} = \frac{N_{als} - N_{al}}{T_{al}}$	Calculation of the normalized aldosterone concentration N_{al} using the time constant T_{al}.	Karaaslan 2005
129	$C_{al} = C_{al\_norm} \cdot N_{al}$	Plasma aldosterone concentration C_{al} is equal to the product of its normalized concentration N_{al} and the normal value C_{al_norm}.	Karaaslan 2005
**Hormonal system**			
130	$C_{anp} = C_{anp\_norm} \cdot \left(7.427 - \frac{6.554}{1 + \exp\left(P_{ra} - 3.762\right)}\right)$	Concentration of atriopeptin C_{anp} dependes on the right atrial pressure P_{ra} and the normal level of the hormone C_{anp_norm}.	Karaaslan 2005
131	$osmolality = 1.86 \cdot C_{sod} + glucose + urea + 9$	Serum osmolality (*osmolality*) is the function of the sodium concentration C_{sod}, as well as the levels of glucose (*glucose*) and urea (*urea*) in blood.	Dorwart Bhagat
132	$C_{adh} = \max\left(0, 0.23 \cdot \left(osmolality - 271\right)\right)$	Antidiuretic hormone (vasopressin) concentration C_{adh} depends on the serum osmolality.	Hammer
**Diuresis**			
133	$\Phi_{t\_wreab} = \eta_{pt\_sodreab} \cdot GFR + \left(1 - \eta_{pt\_sodreab}\right) \cdot GFR \cdot \mu_{adh}$	Tubular water reabsorption rate Φ_{t_wreab} depends on the glomerular filtration rate *GFR*. In the absence of significant amounts of poorly reabsorbable solutes, the fraction of the water load passively reabsorbed in the proximal tubule is equal to the fraction of the sodium load reabsorbed $\eta_{pt\_sodreab}$ [Uttamsingh]. Taking into account details of laboratory measurements, this fraction also includes sodium reabsorbed in the loop of Henle [Seidlerová]. The rate of fluid reabsorption from the distal tubules and collecting ducts (the second term in the formula) depends on the influence of plasma vasopressin concentration μ_{adh} [Uttamsingh].	–
134	$\mu_{adh} = \begin{cases} 0.0, & C_{adh} \le 0.765 \\ 0.383 \cdot C_{adh} - 0.293, & 0.765 < C_{adh} \le 3 \\ -0.0383 \cdot C_{adh}^2 + 0.364 \cdot C_{adh} + 0.109, & 3 < C_{adh} \le 5 \\ 0.0012 \cdot C_{adh} + 0.9653, & C_{adh} > 5 \end{cases}$	Effect of plasma vasopressin concentration C_{adh} on the rate of fluid reabsorption from distal tubules and collecting ducts based on the experimental data [Dehaven].	Uttamsingh

135	$\Phi_u = GFR - \Phi_{t\_wreab}$	Urine flow rate Φ_u is the difference between the glomerular filtration rate and the tubular water reabsorption rate.	Karaaslan 2005
**Body fluids**			
136	$\Phi_{win} = \Phi_{win\_norm} \cdot \left(0.25 + 1.5 \cdot \left(1 + exp\left(2 - 2 \cdot \frac{C_{adh}}{C_{adh\_norm}}\right)\right)^{-1}\right)$	Water intake Φ_{win} is the function taking the nominal value of water consumption Φ_{win_norm} and the normalized level of vasopressin concentration C_{adh}.	Hallow 2017a
137	$\frac{dTBW}{dt} = \Phi_{win} - \Phi_u$	Total body water *TBW* is calculated using the difference between water intake Φ_{win} and urine flow Φ_u.	–
138	$V = 111.5 \cdot TBW + 650$	Total blood volume *V* as the linear function of *TBW*.	Moore 1967
139	$V_{ecf} = 0.37 \cdot TBW + 2.7$	Extracellular fluid volume V_{ecf} as the linear function of *TBW*.	Moore 1967
**Glomerular filtration**			
140	$R_{aa} = R_{aa\_0} \cdot \beta_{rsna} \cdot \Sigma_{tgf} \cdot \Sigma_{myo} \cdot \Psi_{AT1\_aa}$	Single afferent arteriole resistance R_{aa} is equal to the nominal value R_{aa_0} multiplied by the factors representing various effects listed below.	Hallow 2014
141	$R_{aa\_0} = 1.25E8 \cdot \frac{128 \cdot Vis \cdot L_{aa}}{\pi \cdot d_{aa}^4}$	Nominal value of the single afferent arteriole resistance R_{aa_0} can be defined according to the Poiseuille's law based on the arteriole diameter d_{aa}, length L_{aa}, and blood viscosity *Vis*. The coefficient 1.25E8 is used to convert units from cP·μm^{-3} to mmHg·min· l^{-1}:<br>1 cP = 10^{-3} Pa·s, 1 Pa = 0.00750062 mmHg, 1 m^3 = 10^3 liters.	Hallow 2017a
142	$\beta_{rsna} = 1.5 \cdot (RSNA - 1) + 1$	Effect of the renal sympathetic nerve activity *RSNA* on R_{aa} and $R_{preglom}$.	Karaaslan 2005

143	$\frac{d\Sigma_{tgf}}{dt}=0.3408+3.449\cdot\left(3.88+\exp\left(\frac{\Phi_{md\_sod}-3.859}{-0.9617}\right)\right)^{-1}-\Sigma_{tgf}$	Tubuloglomerular feedback signal is modeled as the function of macula densa sodium flow Φ_{md_sod}.	Karaaslan 2005[7]
144	$\frac{d\Sigma_{myo}}{dt}=sl_{Pgh}\cdot\frac{P_{gh}}{P_{gh\_norm}}+1-sl_{Pgh}-\Sigma_{myo}$	Myogenic autoregulation signal Σ_{myo} is calculated according to [Hallow, 2014] by the formula: $$\Sigma_{myo}=c_{GP\_autoreg}\cdot\left(\frac{P_{gh}}{P_{gh\_nom}}-1\right)$$ with the constants $c_{GP\_autoreg}=5$ and $P_{gh\_nom}=60$ mmHg. In the case of values $P_{gh}<60$ mmHg within the normal range [Škrtić], this formula gives the negative values for R_{aa} and $R_{preglom}$, however, this contradicts the physiological meaning of these variables. In this regard, we introduced 1 as a term in the right side of the formula, and as the result for $P_{gh}=60$ mmHg, we received the signal $\Sigma_{myo}=1$. Further, by analogy with Σ_{tgf}, we converted the algebraic equation for Σ_{myo} to the differential one in order to remove the cyclical dependency of R_{aa}, P_{gh}, Σ_{myo}, *RBF*, R_a, and $R_{preglom}$.	–
145	$\Psi_{AT1\_aa}=A_{AT1\_aa}+B_{AT1\_aa}\cdot AT1\_ANGII-\frac{C_{AT1\_aa}}{AT1\_ANGII}$	Effect of AT1-bound angiotensin II (*AT1_ANGII*) on R_{aa}. A_{AT1_aa}, B_{AT1_aa}, and C_{AT1_aa} are constant.	Hallow 2014
146	$R_{preglom}=R_{preglom\_0}\cdot\beta_{rsna}\cdot\Sigma_{myo}\cdot\psi_{AT1\_preglom}$	Resistance of interlobar, arcuate, and interlobular arteries $R_{preglom}$ is equal to the nominal value $R_{preglom\_0}$ multiplied by the factors representing various effects listed below.	Hallow 2014
147	$\psi_{AT1\_preglom}=A_{AT1\_preglom}+B_{AT1\_preglom}\cdot AT1\_ANGII-\frac{C_{AT1\_preglom}}{AT1\_ANGII}$	Effect of AT1-bound angiotensin II (*AT1_ANGII*) on $R_{preglom}$. $A_{AT1\_preglom}$, $B_{AT1\_preglom}$, and $C_{AT1\_preglom}$ are constant.	Hallow 2014

[7] In the model [Karaaslan, 2005], the formula is determined as $\Sigma_{tgf}=0.3408+3.449\cdot\left(3.88+\exp\left(-\left(\Phi_{md\_sod}-3.859\right)/0.9617\right)\right)^{-1}$. In this case, the equations for Σ_{tgf}, Φ_{rb}, Φ_{md_sod}, Φ_{filsod}, Φ_{gfilt}, $\Phi_{pt\_sodreab}$, R_{aa}, P_{gh}, γ_{filsod} and $\eta_{pt\_sodreab}$ form a cyclical dependency. Since the algebraic solver in BioUML (the software used for our modeling) is slower and more likely to loop than the differential equation solver, and this can be significant for the model optimization during generation of virtual populations, we converted the algebraic equation to the differential one by the scheme: $\Sigma_{tgf}=f\left(\Phi_{md\_sod}\right)$ => $d\Sigma_{tgf}/dt=1/T\cdot\left(f\left(\Phi_{md\_sod}\right)-\Sigma_{tgf}\right)$. Such convertion means that Σ_{tgf} takes a value $f\left(\Phi_{md\_sod}\right)$ not immediately, but with the delay *T*. This approach is used, for example, in [Kofranek].

148	$R_a = \frac{R_{aa}}{N_{nephrons}} + R_{preglom}$	Resistance of afferent vessels R_a is equal to the sum of $R_{preglom}$ and the resistance of all afferent arterioles (which number is $N_{nephrons}$).	Hallow 2014
149	$R_{a\_dyne} = 79.68 \cdot R_a$	Conversion R_a units from mmHg·min· l^{-1} to dyn·s·cm^{-5}: $\frac{\text{mmHg}\cdot\text{min}}{\text{l}} = \frac{60}{1000}\cdot\frac{\text{mmHg}\cdot\text{s}}{\text{ml}} = \frac{60}{1000}\cdot 1328 \cdot \frac{\text{dyn}\cdot\text{s}}{\text{cm}^5}$ using the coefficient 1328 taken from the work [Škrtić].	–
150	$R_{ea} = R_{ea\_0} \cdot \Psi_{AT1\_ea}$	Single efferent arteriole resistance R_{ea} is equal to the nominal value R_{ea_0} multiplied by the factors representing various effects listed below.	Hallow 2014
151	$R_{ea\_0} = 1.25E8 \cdot \frac{128 \cdot Vis \cdot L_{ea}}{\pi \cdot d_{ea}^4}$	Nominal value of the single efferent arteriole resistance R_{ea_0} can be defined according to the Poiseuille's law based on the arteriole diameter d_{ea}, length L_{ea}, and blood viscosity *Vis*. The coefficient 1.25E8 is used similar to R_{aa_0}.	Hallow 2017a
152	$\Psi_{AT1\_ea} = A_{AT1\_ea} + B_{AT1\_ea} \cdot AT1\_ANGII - \frac{C_{AT1\_ea}}{AT1\_ANGII}$	Effect of AT1-bound angiotensin II (*AT1_ANGII*) on R_{ea}. A_{AT1_ea}, B_{AT1_ea}, and C_{AT1_ea} are constarnts.	Hallow 2014
153	$R_e = \frac{R_{ea}}{N_{nephrons}}$	Resistance of all efferent arterioles R_e (which number is $N_{nephrons}$).	Hallow 2014
154	$R_{e\_dyne} = 79.68 \cdot R_e$	Conversion of R_e units from mmHg·min· l^{-1} to dyn·s·cm^{-5} (as for R_a).	–
155	$RVR = R_a + \frac{RBF - GFR}{RBF} \cdot R_e + R_v$	Renal vascular resistance *RVR* is equal to the sum of R_a, R_e (with a coefficient characterizing decrease in *RBF* through the efferent arterioles due to the glomerular filtration rate *GFR*) and the constant renal venous resistance R_v.	Gómez
156	$RBF = \frac{MAP - P_v + K_{FG} \cdot R_e \cdot \left(MAP - P_B - P_{go}\right)}{R_a + R_e + R_v + K_{FG} \cdot R_e \cdot R_a}$	Calculation of the renal blood flow *RBF*. For the formula derivation, see appendix B.	App. B
157	$P_{gh} = MAP - RBF \cdot R_a$	Calculation of the glomerular hydrostatic pressure P_{gh} according to the Ohm's law.	Gómez
158	$RPF = RBF \cdot \left(1 - 0.01 \cdot Hct\right)$	Effective renal plasma flow *RPF* is determined from hematocrit *Hct* and the *RBF* value.	Škrtić

159	$FF = \frac{GFR}{RPF}$	Filtration fraction *FF* is the ratio of the glomerular filtration rate *GFR* to the renal plasma flow *RPF*.	Škrtić
160	$C_M = 0.1 \cdot \frac{TP}{FF} \cdot \ln\left(\frac{1}{1 - FF}\right)$	Plasma protein mean concentration within the glomerular capillaries C_M dependes on the total protein level *TP* and the value of *FF*.	Škrtić
161	$\frac{dP_{go}}{dt} = 5 \cdot (C_M - 2) - P_{go}$	Glomerular capillary oncotic pressure P_{go} is defined by the value of C_M.	Škrtić
162	$K_{FG} = K_{FG\_0} \cdot \left(sl_{KFG} \cdot \frac{AT1\_ANGII_{norm}}{AT1\_ANGII} + 1 - sl_{KFG} \right)$	Angiotensin II provides a reduction in the glomerular filtration coefficient K_{FG} [Blantz] via decrease in the total filtering surface area because of mesangial cell contraction [Schmitt]. Angiotensin II receptors on mesangial cells belong to the AT1 subtype [Ardaillou]. Thus, for calculation of K_{FG}, we considered the product of the normal value K_{FG_0} and the linear function (with a slope $0 < sl_{KFG} < 1$) expressing inversely proportional relationship between K_{FG} and the normalized concentration of *AT1_ANGII*. The function is chosen so that the value $AT1\_ANGII = AT1\_ANGII_{norm}$ gives the result $K_{FG} = K_{FG\_0}$.	–
163	$GFR = K_{FG} \cdot (P_{gh} - P_B - P_{go})$	Glomerular filtration rate *GFR* depends on P_{gh}, P_{go}, the hydrostatic pressure in the Bowman's space P_B, and the filtration coefficient K_{FG}.	Gómez

Table A3. List of the model constants with values for a healthy person.

№	Parameters	Entity	Pathological conditions and factors affecting the value	Initial values		Reference values		Units
				Values	Sources	Norms	Sources	
001	A_1	Total metabolic intensity	Age, weight [Henry]	0.0008	Proshin MATLAB	0.00032 – 0.00128[8]	–	ml^{-1}
002	A_{10}	–	–	0.8	Proshin MATLAB	–	–	$ml \cdot s^{-1} \cdot mmHg^{-2}$
003	A_{11}	Systemic and pulmonary venous tone	Changing the sensitivity of venous vessel walls to nervous and hormonal influences [S-D]	0.0325	Proshin MATLAB	–	–	$ml \cdot mmHg^{-1}$
004	A_{12}	Reactivity of cardiac center	–	0.19336	Proshin MATLAB	–	–	s^{-1}
005	A_{13}	Sympathetic sensitivity of the pulmonary microvessels	Changing the sensitivity of pulmonary microvessels to nervous and hormonal influences [S-D]	0.65	Proshin MATLAB	–	–	$ml \cdot mmHg^{-1}$
006	A_{14}	Pulmonary microvessel sensitivity to oxygen debt	–	0.08265	Proshin MATLAB	–	–	$s^{-1} \cdot mmHg^{-1}$
007	A_{15}	–	–	22.0	Proshin MATLAB	–	–	$s^{-1} \cdot mmHg^{-1}$
008	A_{16}	–	–	3.3	Proshin MATLAB	–	–	$ml \cdot s^{-1} \cdot mmHg^{-2}$
009	A_{18}	Sympathetic sensitivity of the pulmonary arteries, $A_{18} < A_8$ [S-D]	Pulmonary vasospasm [S-D]	40.0	Proshin MATLAB	–	–	$ml \cdot s$
010	A_{19}	Pulmonary arterial tone, $A_{19} < A_9$ [S-D]	Changing the elastic properties of the pulmonary artery walls [S-D]	0.02 (0.06)	FP[9] (Proshin MATLAB)	–	–	$mmHg \cdot s \cdot ml^{-1}$
011	A_2	Organism functional status	Low tolerance to the exercise stress in metabolic disorders (hyperthyroidism, hypothyroidism, diabetes mellitus), asthenic conditions and others [S-D]	0.3752	Proshin MATLAB	–	–	–
012	A_3	Sympathetic sensitivity of the systemic microvessels	Vegetative-vascular crisis, hypertension [S-D]	0.1	Proshin MATLAB	–	–	$ml \cdot mmHg^{-1}$

[8] We took the initial value $A_1 = 0.0008$ ml^{-1} from the Proshin and Solodyannikov model implementation in MATLAB and considered its deviation within a virtual population by 60%, which in accordance with [Henry] is the allowable fluctuation in the basal metabolic rate (closely related to the oxygen consumption) in people with the same weight.

[9] The "FP" (fitted parameter) notation means that the corresponding value was identified so that the model dynamics in the case of a healthy person lies within physiological norms given in Table A4 below.

013	A_4	Systemic microvessel sensitivity to oxygen debt	Vegetative-vascular crisis [S-D]	0.031537	Proshin MATLAB	–	–	$s^{-1}\cdot mmHg^{-1}$
014	A_5	–	–	22.0	Proshin MATLAB	–	–	$s\cdot mmHg^{-1}$
015	A_6	–	–	3.3	Proshin MATLAB	–	–	$ml\cdot s^{-1}\cdot mmHg^{-2}$
016	A_7	–	–	4.0	Proshin MATLAB	–	–	$mmHg^{-1}$
017	A_8	Sympathetic sensitivity of the systemic arteries, $A_8 > A_{18}$ [S-D]	Vascular spasm, e.g., vascular collapse [S-D]	45.0	Proshin MATLAB	–	–	ml·s
018	A_9	Systemic arterial tone, $A_9 > A_{19}$ [S-D]	Neurocirculatory dystonia [S-D], hypertension [Amberg]	0.07	Proshin MATLAB	–	–	$mmHg\cdot s\cdot ml^{-1}$
019	A_{AT1_aa}	Constant for AT1-bound angiotensin II effect on afferent arteriole resistance	–	1.3754 (0.8)	FP (Hallow 2014)	–	–	–
020	A_{AT1_ALVL}	Constant for AT1-bound angiotensin II effect on blood flow through systemic microvessels	–	1.7323	FP	–	–	–
021	A_{AT1_ea}	Constant for AT1-bound angiotensin II effect on efferent arteriole resistance	–	1.6601 (0.925)	FP (Hallow 2014)	–	–	–
022	$A_{AT1\_preglom}$	Constant for AT1-bound angiotensin II effect on resistance of interlobar, arcuate, and interlobular arteries	–	0.4464 (0.8)	FP (Hallow 2014)	–	–	–
023	AO_2	Arterial oxygen content	–	$\frac{He\cdot C_H\cdot SpO_2}{1000}\approx 0.188$	S-D	0.145 – 0.244	Hattori	–
024	$AT1\_ANGII_{norm}$	The normal level of AT1-bound angiotensin II	–	16.63	Hallow 2017a	–	–	$fmol\cdot ml^{-1}$
025	B_{AT1_aa}	Constant for AT1-bound angiotensin II effect on afferent arteriole resistance	–	0.0549 (0.055)	FP (Hallow 2014)	–	–	–
026	B_{AT1_ALVL}	Constant for AT1-bound angiotensin II effect on blood flow through systemic microvessels	–	0.0032	FP	–	–	–
027	B_{AT1_ea}	Constant for AT1-bound angiotensin II effect on efferent arteriole resistance	–	0.0383 (0.05)	FP (Hallow 2014)	–	–	–

028	$B_{AT1\_preglom}$	Constant for AT1-bound angiotensin II effect on resistance of interlobar, arcuate, and interlobular arteries	–	0.0661 (0.055)	FP (Hallow 2014)	–	–	–
029	c_{ACE}	Rate of conversion of angiotensin I to angiotensin II by ACE	–	0.9016666	Hallow 2014	–	–	min^{-1}
030	c_{ACE2}	Rate of conversion of angiotensin II to angiotensin-(1-7) by ACE2	–	0.04	Hallow 2014	–	–	min^{-1}
031	C_{adh_norm}	Normal plasma level of antidiuretic hormone (vasopressin)	–	4.7	–	1.0 – 13.3	Yarmohammadi	$pg \cdot ml^{-1}$
032	C_{al_norm}	Normal plasma concentration of aldosterone	–	85	Karaaslan 2005	70 – 300	Fischbach	$pg \cdot ml^{-1}$
033	c_{ANGII_ANGIV}	Rate of conversion of angiotensin II to angiotensin IV	–	0.3916666	Hallow 2014	–	–	min^{-1}
034	C_{anp_norm}	Normal plasma level of atrial natriuretic peptide (atriopeptin)	–	36.0	Karaaslan 2005	7.4 – 152.0	Cannone Nozuki	$ng \cdot l^{-1}$
035	c_{AT1}	Rate of angiotensin II binding to AT1 receptors	–	0.1966666	Hallow 2014	–	–	min^{-1}
036	C_{AT1_aa}	Constant for AT1-bound angiotensin II effect on afferent arteriole resistance	–	0.1437 (0.185)	FP (Hallow 2014)	–	–	–
037	C_{AT1_ALVL}	Constant for AT1-bound angiotensin II effect on blood flow through systemic microvessels	–	0.2170	FP	–	–	–
038	C_{AT1_ea}	Constant for AT1-bound angiotensin II effect on efferent arteriole resistance	–	0.2441 (0.17)	FP (Hallow 2014)	–	–	–
039	$C_{AT1\_preglom}$	Constant for AT1-bound angiotensin II effect on resistance of interlobar, arcuate, and interlobular arteries	–	0.2133 (0.185)	FP (Hallow 2014)	–	–	–
040	c_{AT2}	Rate of angiotensin II binding to AT2 receptors	–	0.065	Hallow 2014	–	–	min^{-1}
041	c_{chym}	Rate of conversion of angiotensin I to angiotensin II by chymase	–	0.01833333	Hallow 2014	–	–	min^{-1}
042	C_H	Oxygen capacity of hemoglobin	Carbon monoxide poisoning [S-D]	1.34	S-D	1.32 – 1.39	Dijkhuizen	$ml \cdot g^{-1}$
043	C_K	Serum potassium concentration	Hypokalemia: alkalemia, states of high endogenous or exogenous insulin or catecholamines, marked potassium deficiency (poor dietary	4.5	–	3.5 – 5.5	Rastegar	$mEq \cdot l^{-1}$

			intake, gastrointestinal disorders); <u>Hyperkalemia</u>: thrombocytosis, leukocytosis, significant hemolysis, acidemia, sudden increase in plasma osmolality, massive tissue breakdown, β-blockade during and immediately after exercise, decrease in renal excretion of potassium [Rastegar]					
044	c_{nep}	Rate of conversion of angiotensin I to angiotensin-(1-7) by neprilysin	–	0.01833333	Hallow 2014	–	–	min^{-1}
045	d_{aa}	Afferent arteriole diameter	–	23.1	–	21.5 ± 2.4	Neal	µm
046	d_{ea}	Efferent arteriole diameter	–	16.9	FP	15.9 ± 2.4	Neal	µm
047	$FS_{threshold}$	Frank-Starling law action threshold	–	$m/70 \cdot FS_{threshold0}$	Proshin MATLAB	–	–	ml
048	$FS_{threshold0}$	Mean threshold of the Frank-Starling law action	–	20.0	Proshin MATLAB	–	–	ml
049	G_{AL0}	Basic systemic arterial elastance	Hypertension [Haluska]	0.65	Proshin MATLAB	0.33 – 1.00	Table A4	$mmHg \cdot ml^{-1}$
050	G_{AR0}	Basic pulmonary arterial elastance	Pulmonary hypertension [Thenappan]	0.125	Proshin MATLAB	0.08 – 0.26	Table A4	$mmHg \cdot ml^{-1}$
051	G_{HL}	LV wall elasticity	Hypertension [Safar, 1985], LV hypertrophy [S-D], exercise regularity [Bhella]	0.0824 (0.16)	FP (Zhang, 2008)	0.01 – 0.43	Zhang, 2008	$mmHg \cdot ml^{-1}$
052	G_{HR}	RV wall elasticity	RV hypertrophy [S-D]	0.0471 (0.16)	FP (Zhang, 2008)	0.01 – 0.43	Zhang, 2008	$mmHg \cdot ml^{-1}$
053	G_{VL0}	Basic elasticity of systemic veins	Changing the elastic properties of systemic venous vessels [S-D]	0.0112 (0.0375)	FP (Proshin MATLAB)	–	–	$mmHg \cdot ml^{-1}$
054	G_{VR0}	Basic elasticity of pulmonary veins	Changing the elastic properties of pulmonary venous vessels [S-D]	0.0108 (0.0375)	FP (Proshin MATLAB)	–	–	$mmHg \cdot ml^{-1}$
055	*glucose*	Plasma glucose concentration	<u>Hyperglycemia</u>: diabetes mellitus, physiological increase in glucose (stress, exercise, smoking, etc.), endocrine pathology, pancreatic diseases (acute and chronic pancreatitis, pancreatic tumors, etc.), chronic liver and kidney diseases, cerebral hemorrhage,	5.0	–	3.9 – 6.1	Dedov	$mmol \cdot l^{-1}$

			myocardial infarction, presence of antibodies to insulin receptors; Hypoglycemia: pancreatic diseases, endocrine pathology, severe liver diseases (cirrhosis, hepatitis, carcinoma, etc.), malignant non-pancreatic tumors, fermentopathies, nutritional disorders (prolonged fasting, malabsorption syndrome), functional disorders (vegetative disorders, gastroenterostomy, etc.), intense physical activity, feverish states [Andrushkevich]					
056	h_{ANG17}	Half-life of angiotensin (1-7)	–	30.0	Hallow 2014	19.2–51.6[10]	Rodgers	min
057	h_{ANGI}	Half-life of angiotensin I	–	0.25	–	0.25±0.08[11]	Admiraal	min
058	h_{ANGII}	Half-life of angiotensin II	–	0.9	–	M: 1.0<br>W: 0.8	Magness<br>Donato	min
059	h_{ANGIV}	Half-life of angiotensin IV	–	0.5	Hallow 2014	–	–	min
060	h_{AT1}	Half-life of AT1-bound angiotensin II	–	12.0	Hallow 2014	12.0[12]	Inada	min
061	h_{AT2}	Half-life of AT2-bound angiotensin II	–	12.0	Hallow 2014	–	–	min
062	h_{renin}	Half-life of circulating renin	Malignant hypertension [Skrabal, 1974]	12.0	Hallow 2014	10.0 – 15.0[13]	Skrabal 1974	min
063	*Hct*	Hematocrit	–	40.0	Guyton	M: 40 – 54<br>W: 36 – 48	Billett	%

[10] *In vivo* measurement. The mean plasma half-life of angiotensin (1-7) administered before and after chemotherapy in patients with newly diagnosed breast cancer was 0.49 h (29 min), range 0.32 – 0.86 h (19.2 – 51.6 min) [Rodgers]. A similar range 0.42 – 0.61 h was obtained for higher doses of *ANG*17 administered in patients with advanced solid tumors refractory to standard therapy [Petty].

[11] *In vivo* measurement. The estimate obtained for six subjects with essential hypertension after the infusion of rather high quantities of unlabeled angiotensin I in combination with radiolabeled *ANGI* (^{125}I-*ANG I*). High *ANGI* doses was given to minimize the contribution of endogenous *ANGI* to the plasma levels measured during the infusion [Admiraal].

[12] *In vitro* measurement. The value was obtained using bovine adrenal cortical membrane [Inada], and corresponds to the range 13 – 23 min obtained in [Glossmann] also for bovine adrenal cortex.

[13] *In vivo* measurement. Half-life of plasma renin activity in 3 normal subjects after maximal stimulation and subsequent inhibition of renin release by the intravenous administration of propranolol were 10, 13 and 15 minutes, and in the patient with malignant hypertension immediately after bilateral nephreetomy – 1 hour 22 minutes [Skrabal, 1974]. The values reported in the literature range from about 10 to 165 min, which can be explained, at least partly, by differences in the methods used for measuring renin [Derkx].

064	*He*	Hemoglobin	Anemia [S-D]	143.0	–	M:140–180 W:120–160	Billett	$g \cdot l^{-1}$
065	$Heart_{Base}$	Basic activity of the cardiac center	–	0.1357 (0.5)	FP (Proshin MATLAB)	–	–	–
066	$Heart_{Baro}$	Baroreceptor sensitivity of the cardiac center	Hyper- or hypoactivity of the vegetative nervous system, blockade of baroreceptors [S-D]	0.6	Proshin MATLAB	–	–	–
067	$Heart_{Oxygen}$	Weariness sensitivity of the cardiac center	Hyper- or hypoactivity of the vegetative nervous system, organism tolerance to the exercise stress under some functional disorders, such as neurocirculatory dystonia [S-D]	1.75	Proshin MATLAB	–	–	–
068	$Heart_{Stress}$	Stress sensitivity of the cardiac center	Hyper- or hypoactivity of the vegetative nervous system [S-D]	1.5	Proshin MATLAB	–	–	–
069	$Heart_{VO2}$	Respiratory sensitivity of the cardiac center	Hyper- or hypoactivity of the vegetative nervous system [S-D]	1.0	Proshin MATLAB	–	–	–
070	k_{AL}	Fraction ω_{AL} of V_{AL} at the initial moment of time	–	0.91 (0.87[14])	FP (Proshin MATLAB)	–	–	–
071	k_{AR}	Fraction ω_{AR} of V_{AR} at the initial moment of time	–	0.82 (0.8[14])	FP (Proshin MATLAB)	–	–	–
072	K_{FG_0}	Normal filtration coefficient	–	0.005202	Škrtić	0.0039–0.0162	Hoang	$l \cdot min^{-1} \cdot mmHg^{-1}$
073	k_{HL}	Fraction ω_{HL} of V_{HL} at the initial moment of time	–	0.0	Rosalina Paeme	0.0 – 0.3[15]	–	–
074	K_{HLAL}	Aortic valve regurgitation coefficient	Aortic valve failure [S-D]	0.0	S-D	0.0	S-D	–
075	k_{HR}	Fraction ω_{HR} of V_{HR} at the initial moment of time	–	0.0	Paeme	0.0 – 0.3[15]	–	–
076	K_{HRAR}	Pulmonary valve regurgitation coefficient	Pulmonary valve failure [S-D]	0.0	S-D	0.0	S-D	–

[14] The model implementation by Proshin and Solodyannikov in MATLAB provides the initial volumes $V_{AL0} = 300$ ml, $V_{AR0} = 300$ ml, $V_{VR0} = 1135$ ml, $V_{HL0} = 140$ ml, and $V_{HR0} = 140$ ml, the body weight $m = 70.86$, and the formula for the allometric scaling $mass_{volume}$, according to which the body weight of 70 kg corresponds to the normal circulating blood volume of 4725 ml. Based on this, we found the value $V = 70.86 \cdot 4725/70 \approx 4783$ ml and received $V_{VV0} = V - V_{AL0} - V_{AR0} - V_{VR0} - V_{HL0} - V_{HR0} \approx 2768$ ml. Taking into account the normal values of the unstressed volumes $\omega_{AL0} = 260$ ml, $\omega_{AR0} = 240$ ml, $\omega_{HL0} = 40$ ml, $\omega_{HR0} = 40$ ml, $\omega_{VL0} = 2650$ ml, and $\omega_{VR0} = 1075$ ml, we calculated the corresponding fractions $k_{AL} \approx 0.87$, $k_{AR} = 0.8$, $k_{HL} \approx 0.3$, $k_{HR} \approx 0.3$, $k_{VL} \approx 0.96$ and $k_{VR} = 0.95$.

[15] The rationale for this range is given in Table A4 below in description of the parameters ω_{HL} and ω_{HR}.

077	k_{VL}	Fraction ω_{VL} of V_{VL} at the initial moment of time	–	1.0 (0.96[14])	FP (Proshin MATLAB)	–	–	–
078	K_{VLHR}	Tricuspid valve regurgitation coefficient	Tricuspid valve failure [S-D]	0.0	S-D	0.0	S-D	–
079	k_{VR}	Fraction ω_{VR} of V_{VR} at the initial moment of time	–	0.97 (0.95[14])	FP (Proshin MATLAB)	–	–	–
080	K_{VRHL}	Mitral valve regurgitation coefficient	Mitral valve failure [S-D]	0.0	S-D	0.0	S-D	–
081	K_{L0}	Inotropic status of the LV	Decreased pumping function of the heart (myocardial infarction, heart failure of different pathogenesis, etc.) [S-D].	0.55	S-D	0.55 – 0.60	S-D	–
082	K_{R0}	Inotropic status of the RV	Similar to K_{L0} [S-D].	0.55	S-D	0.55 – 0.60	S-D	–
083	L_{aa}	Afferent arteriole length	–	112.0	Neal	–	–	µm
084	L_{ea}	Efferent arteriole length	–	138.0	Neal	–	–	µm
085	m	Body weight	–	70	–	–	–	kg
086	$N_{nephrons}$	Number of nephrons in the kidneys	Renal pathology, hypertension, birth weight, age [Bertram]	2.75E6 (2.0E6)	FP (Hallow 2014)	2 x 0.2E6 – 2 x 2.7E6[16]	Bertram	–
087	N_{rsna}	Normalized renal sympathetic nerve activity	–	1.0	Karaaslan 2005	–	–	–
088	n_{ε_dt}	Normal fractional distal sodium reabsorption	–	0.50	Karaaslan 2005	–	–	–
089	n_{η_cd}	Normal fractional collecting duct sodium reabsorption	–	0.90 (0.93)	FP (Karaaslan 2005)	–	–	–
090	n_{η_pt}	Normal fractional sodium reabsorption in the proximal tubule and the loop of Henle	–	0.80	Karaaslan 2005	M: 0.795 ± 0.083<br>W: 0.793 ± 0.084	Seidlerová	–
091	$n_{\varepsilon\_dt} + n_{\eta\_cd} - -n_{\eta\_cd} \cdot n_{\varepsilon\_dt}$	Normal fractional sodium reabsorption in the distal tubule and subsequent nephron parts	–	0.95	Karaaslan 2005	M: 0.946 ± 0.032<br>W: 0.950 ± 0.031	Seidlerová	–
092	P_0	Baroreceptor sensitivity threshold	Hyperfunction of the circulatory system, baroreceptor blockade [S-D]	2.0	Proshin MATLAB	–	–	mmHg

[16] The article [Bertam] gives a range of 0.2E6 – 2.7E6 for the nephron number in one human kidney with an average value of 895711. $N_{nephrons}$ is the number of nephrons in two kidneys.

093	P_B	Hydrostatic pressure in the Bowman's space	–	10.0	Gómez	10.0 – 15.0	Digne-Malcolm	mmHg
094	P_{gh_norm}	Normal glomerular hydrostatic pressure	–	60.0	Hallow 2014	48.0 – 63.0	Guberina	mmHg
095	P_v	Renal venous pressure	Hypertension (with hydropenia or during sustained isotonlc saline volume expansion) [Willassen]	6.0	Digne-Malcolm	–	–	mmHg
096	PRC_{nom}	Nominal value of plasma renin concentration	–	20.0 (15.0)	FP (Hallow 2014)	3.42 – 69.4	Perschel	$pg \cdot ml^{-1}$
097	$R_{preglom\_0}$	Nominal resistance of interlobar, arcuate and interlobular arteries	–	10.0 (14.0)	FP (Hallow 2017a)	10.0 – 20.0	Hallow 2017a	$mmHg \cdot min \cdot l^{-1}$
098	R_v	Renal venous resistance	–	15.0	App. B	11.3 – 20.1	App. B	$mmHg \cdot min \cdot l^{-1}$
099	RO_2	Oxygen demand	Exercise stress [S-D]	$m/70 \cdot RO_{20}$	Proshin MATLAB	–	–	$ml \cdot s^{-1}$
100	RO_{20}	Mean body oxygen demand	Exercise stress [S-D]	4.2	–	≈ 4.2	Treacher	$ml \cdot s^{-1}$
101	sl_{baro}	Slope of the linear function of AT1-bound angiotensin II effect on baroreceptor activity	–	0.3457	FP	–	–	–
102	sl_{KFG}	Slope of the linear function of AT1-bound angiotensin II effect on filtration coefficient	–	0.0924	FP	–	–	–
103	sl_{Pgh}	Slope of the linear function of glomerular hydrostatic pressure effect on myogenic autoregulation signal	–	0.8294	FP	–	–	–
104	sl_{stress}	Slope of the linear function of AT1-bound angiotensin II effect on stress receptor activity	–	0.1349	FP	–	–	–
105	$slope_{AT1\_PRC}$	Constant for AT1-bound angiotensin II effect on renin secretion rate	–	1.2	Hallow 2017a	–	–	–
106	SpO_2	Arterial oxygen saturation	–	0.98	–	0.95 – 0.99	Goldberg	–
107	st	Stress factor, the level of steroid hormones in the blood	Stress mode [S-D]	0.25	Proshin MATLAB	0.0 – 1.0	S-D	–
108	SV_{max}	Theoretical maximum stroke volume	–	$m/70 \cdot SV_{max0}$	Proshin MATLAB	–	–	ml
109	SV_{max0}	Average theoretical maximum stroke volume	–	200.0	Proshin MATLAB	–	–	ml
110	T_{al}	Time constant for aldosterone secretion	–	30.0	Karaaslan 2005	–	–	min

111	*TP*	Total protein	Albumin: increase – acute dehydration; decrease – end-stage liver disease, intestinal malabsorption syndromes, protein-calorie malnutrition, nephrotic syndrome, severe burns.<br>Globulin: increase – stress, acute inflammation, tissue necrosis, chronic infections, nephrotic syndrome, severe iron deficiency, connective tissue diseases, liver disease, multiple myeloma, lymphoma, etc.; decrease – congenital α_1 antitrypsin deficiency, hemolytic reaction, congenital immune deficiency syndromes, chronic lymphocytic leukemia, nephrotic syndrome [Busher].	70.0	–	60.0 – 80.0<br>66.0 – 86.0	Busher<br>Gardner	$g \cdot l^{-1}$
112	*urea*	Plasma urea concentration	Renal (glomerulonephritis, renal amyloidosis, pyelonephritis, etc.) and extrarenal (heart failure, severe bleeding, shock, etc.) retention azotemia, production azotemia (cachexia, leukemia, increased physical activity, etc.), liver dysfunction, pregnancy, low protein diet, phosphorus poisoning, acromegaly, malabsorption syndrome [Andrushkevich]	5.0	–	1.8 – 7.1	Hosten	$mmol \cdot l^{-1}$
113	*Vis*	Blood viscosity	Heat stress [Keatinge], hypertension [Letcher]	$1.23 \cdot \left(1 - \frac{Hct}{99}\right)^{-n}$<br>$n = a + b \cdot e^{-c \cdot Hct}$<br>$a = 1.7,\ b = 9.86,$<br>$c = 0.0607$	Hund	4.66 ± 0.72[17]	Furukawa	sP

[17] Values for a shear rate of 100 s^{-1}. Experimental data [Brooks] used to derive the viscosity formula in [Hund] correspond to shear rates above 100 s^{-1}, when the viscosity is practically unchanged. Typical ranges of shear rates: 20–200 s^{-1} for veins, 300–800 s^{-1} for large arteries, 500–1600 s^{-1} for arterioles [Kroll].

114	Vis_{norm}	Mean normal blood viscosity	–	4.65[18]	Hund	4.66 ± 0.72	Furukawa	sP
115	X_{PRC_PRA}	Equilibrium ratio of plasma renin activity to plasma renin concentration	–	1.016666	Hallow 2014	–	–	fmol·min^{-1}·pg^{-1}
116	Y_{ALVL0}	Basic conductivity of systemic microvessels (arterioles, capillaries and venules)	Peripheral vascular spasm [S-D]	1.363 (1.2)	FP (Proshin MATLAB)	–	–	ml·s^{-1}·mmHg^{-1}
117	Y_{ARVR0}	Basic conductivity of pulmonary microvessels (arterioles, capillaries and venules)	Pulmonary vasospasm [S-D]	15.0 (4.8)	FP (Proshin MATLAB)	–	–	ml·s^{-1}·mmHg^{-1}
118	Y_{HLAL}	Basic conductivity of the aortic valve and systemic arteries	Aortic stenosis [S-D]	7.0 (13.515)	FP (Proshin MATLAB)	–	–	ml·s^{-1}·mmHg^{-1}
119	Y_{HRAR}	Basic conductivity of the pulmonary valve and arteries	Pulmonary valve stenosis [S-D]	57.0 (15.225)	FP (Proshin MATLAB)	–	–	ml·s^{-1}·mmHg^{-1}
120	Y_{VLHR0}	Basic conductivity of the tricuspid valve and systemic veins	Tricuspid valve stenosis [S-D]	90.0 (120.0)	FP (Proshin MATLAB)	–	–	ml·s^{-1}·mmHg^{-1}
121	Y_{VRHL0}	Basic conductivity of the mitral valve and pulmonary veins	Mitral stenosis [S-D]	57.0 (40.0)	FP (Proshin MATLAB)	–	–	ml·s^{-1}·mmHg^{-1}
122	ξ_{map}	Effect of mean arterial pressure on the aldosterone secretion	–	1.0	Karaaslan 2005	–	–	–
123	τ_{MD_renin}	Constant for macula densa sodium flow effect on renin secretion rate	–	0.0959 (2.0)	FP (Hallow 2017a)	–	–	–
124	$\Phi_{md\_sod\_0}$	Nominal value of macula densa sodium flow rate	–	3.320 (0.7980)	FP (Hallow 2017a)	–	–	mEq·min^{-1}
125	Φ_{sodin}	Sodium intake	–	0.0565 (0.1260)	FP (Karaaslan 2005)	0.035 – 0.174	Luft	mEq·min^{-1}
126	Φ_{win_norm}	Normal value of water intake	–	0.0019	–	0.0019 ± 0.0007[19]	Malisova	l·min^{-1}
127	ω_{AL_nom}	Nominal unstressed volume of systemic arteries	Vasospasm (vascular collapse) [S-D]	$k_{AL} \cdot V_{AL}(0) \approx 573.82$	–	–	–	ml
128	ω_{AR_nom}	Nominal unstressed volume of pulmonary arteries	Pulmonary vasospasm [S-D]	$k_{AR} \cdot V_{AR}(0) \approx 139.21$	–	–	–	ml

[18] The value given by the viscosity formula proposed by Hund et al. [Hund] for hematocrit of 40%.
[19] 2.75 ± 1.01 l/day

129	ω_{HL}	Unstressed LV volume[20]	LV hypertrophy [S-D], heart disease [Senzaki], hypertension [Kelly], [Kass]	$k_{HL}\cdot V_{HL}(0) = 0.0$	–	0.0 – 42.0[20]	–	ml
130	ω_{HR}	Unstressed RV volume[20]	RV hypertrophy [S-D], pulmonary hypertension [Trip]	$k_{HR}\cdot V_{HR}(0) = 0.0$	–	0.0 – 42.0[20]	–	ml
131	ω_{VL}	Unstressed volume of systemic veins	Congestion in the veins: varicose veins, congestive heart failure, etc. [S-D]	$k_{VL}\cdot V_{VL}(0)$ ≈ 3443.90	–	–	–	ml
132	ω_{VR}	Unstressed volume of pulmonary veins	Congestion in the pulmonary veins: pulmonary edema, RV failure, etc. [S-D]	$k_{VR}\cdot V_{VR}(0)$ ≈ 305.83	–	–	–	ml

[20] The variable ω_{HL} (or ω_{HR}) corresponds to the LV (RV) volume V_{HL} (V_{HR}) at zero diastolic pressure P_{HL_D} (P_{HR_D}), and defines a volume-axis intercept of the end-diastolic pressure-volume relation (EDPVR) curve [Paeme]. Theoretically, for a healthy heart, the corresponding end-systolic pressure-volume relation (ESPVR) curve has the same volume intercept (V_0) [Bastos], [Paeme]. Therefore, to assign the reference ranges for ω_{HL} and ω_{HR}, we used the estimates found in the literature for the linear (unless otherwise stated below) ESPVR curves. Figure 6 of the article [Kelly] shows that the LV value V_0 in a young normotensive is approximately –5.0 to 0.0 ml, which is consistent with the value $\omega_{HL} = 0.0$ in the [Rosalina] and [Paeme] models. In [Hayward], for 11 subjects with normal LV function, the values V_0 are negative: −32.5 ± 3.2 ml. The work [Kass] gives estimate –32.5 ± 18.6 ml of the LV value V_0 for 6 patients with normal coronary arteriography and ventriculography, which taking into account the normalization to LV end-diastolic volumes presented for all patients results in the range –0.82 to 0.0 for k_{HL}. The author of the article [Shoucri, 2013] considered the non-linear ESPVR curve and found the LV values 16.62 – 51.37 ml of V_0 (0.15 – 0.28 for k_{HL}) in 10 normal patients. A similar estimate for 9 normal subjects is in another work by the same author: 33.8 – 61.1 ml (0.19 – 0.28 for k_{HL}) [Shoucri, 2015]. The article [Dell'Italia] provides an estimate 24 – 89 ml of the RV values V_0 (0.20 – 0.43 for k_{HR}) in 9 patients with normal coronary anatomy and ventricular function. In work [Brown, 1988], for 8 patients with normal RV function, the values V_0 were –8 to 28 ml/m^2 (–0.12 to 0.40 for k_{HR}). Note that in patients with heart disease, the ranges of V_0 for the LV and the RV can vary significantly. So in the work [Senzaki], an estimate of V_0 for the LV in 87 patients with normal or myopathic hearts is –100 to 200 ml, and in the article [Trip], for patients with pulmonary hypertension, the range –8 to 171 ml is given for the RV values V_0. Based on the above, in the current work, we considered the normal range 0.0 – 0.3 for k_{HL} and k_{HR}, which taking into account the normal initial volumes $V_{HL} = V_{HR} = 140$ ml gives 0.0 – 42.0 ml for ω_{HL} and ω_{HR}.

Table A4. List of the model variables.

№	Variables	Entity	Pathological conditions and factors affecting the value	Values			Units
				Equilibrium[21]	Initial (to generate virtual patients)[22]	Norms[23]	
001	*ANG*17	Plasma angiotensin-(1-7) concentration	Hypertension, pre-eclampsia, hypertrophic myocardial disease, congestive heart failure, myocardial infarct, chronic renal diseases, hepatic cirrhosis, diabetic nephropathy, gestational diabetes [Ribeiro-Oliveira]	14.1233	23.0	22.9 ± 8.8 [Ferrario]	$fmol \cdot ml^{-1}$
002	*ANGI*	Plasma angiotensin I concentration	Hymenoptera venom anaphylaxis [Hermann]	7.5052	7.5 [Hallow, 2017a]	8.2 ± 5.4 [Nussberger]	$fmol \cdot ml^{-1}$
003	*ANGII*	Plasma angiotensin II concentration	Type 1 diabetes [Bojestig], aldosterone level [Wang], hymenoptera venom anaphylaxis [Hermann]	4.7180	4.75 [Hallow, 2017a]	5.2 ± 1.9 [Nussberger]	$fmol \cdot ml^{-1}$
004	*ANGIV*	Plasma angiotensin IV concentration	–	1.3330	0.0	–	$fmol \cdot ml^{-1}$
005	*AT*1*_ANGII*	Concentration of AT1-bound angiotensin II	–	16.0637	16.63 [Hallow, 2017a]	–	$fmol \cdot ml^{-1}$
006	*AT*2*_ANGII*	Concentration of AT2-bound angiotensin II	–	5.3092	4.16 [Hallow, 2017a]	–	$fmol \cdot ml^{-1}$
007	C_{adh}	Plasma concentration of antidiuretic hormone (vasopressin)	Hypertension [Cowley], change in blood osmolality [Hammer]	3.2746	–	1.0 – 13.3 [Yarmohammadi]	$pg \cdot ml^{-1}$
008	C_{al}	Plasma aldosterone concentration	Salt intake [Ishimitsu], [Wambach], hypertension, concentric LV hypertrophy, atrial fibrillation, obesity, chronic kidney disease, high triglycerides, metabolic syndrome [Buglioni], ascites in hepatic cirrhosis [Kuiper], primary and secondary aldosteronism [Glinicki], type 1 diabetes [Škrtić], race [Tu]	108.2091	–	70 – 300 [Fischbach]	$pg \cdot ml^{-1}$

[21] Equilibrium values of the model within the normal ranges for a healthy person (an accuracy of 10^{-4}, the exact values can be found in the BioUML implementation of the model in the website: https://gitlab.sirius-web.org/virtual-patient/blood-pressure-regulation).
[22] The initial values of variables calculated using differential equations or discrete events. They can be used for generation of virtual patients.
[23] Data range or mean ± SD.

009	C_{anp}	Plasma concentration of atrial natriuretic peptide (atriopeptin)	Type 1 diabetes [Bojestig], salt intake, hypertension [Ishimitsu], [Wambach], congestive heart failure, chronic renal failure [Nozuki], plasma aldosterone level in hypertension [Cannone], ventricular pacing [Zullo]	43.7338	–	7.4 – 152.0 [Cannone] [Nozuki]	$ng \cdot l^{-1}$
010	C_M	Plasma protein mean concentration within the glomerular capillaries	–	7.6064	–	–	$g \cdot l^{-1}$
011	C_{sod}	Serum sodium concentration	Hypovolaemia (burns, diarrhoea, vomiting, acute and chronic renal disease, etc.), hypervolaemia (congestive cardiac failure, cirrhosis with ascites, chronic renal failure, etc.), euvolaemia (hypothyroidism, water intoxication, diabetes insipidus, etc.) [Reynolds]	143.1384	–	137 – 147 [Payne]	$mEq \cdot l^{-1}$
012	CO	Cardiac output	Hypertension, exercise [Cléroux], positive-pressure ventilation [Kyhl]	4.9503	–	2.51 – 9.00 (age 18+) [Cattermole]	$l \cdot min^{-1}$
013	$Cycle_{Length}$	Cardiac cycle length (RR-interval)	–	0.7578	0.85 [Proshin MATLAB]	M: 0.624 – 1.284 F: 0.600 – 1.213 [Mason]	s
014	$Cycle_{Time}$	Time of the current cardiac cycle	–	0.5673	0.0	–	s
015	DO_2	Oxygen debt	–	10.8205	0.0 [Proshin MATLAB]	–	ml
016	DTS_L	LV systolic mismatch	–	0.0	–	–	–
017	DTS_R	RV systolic mismatch	–	0.0	–	–	–
018	EF	Ejection fraction	Cardiomyopathy, coronary artery disease [Pagani], chronic heart failure [Dushina]	62.2167	–	≥ 50 [Pfisterer]	%
019	F_{ALVL}	Blood flow through systemic microvessels	–	76.3198	–	–	$ml \cdot s^{-1}$
020	F_{ARVR}	Blood flow through pulmonary microvessels	–	58.4373	–	–	$ml \cdot s^{-1}$

021	F_{HLAL}	Blood flow through the aortic valve	Exercise in healthy individuals and patients with aortic valve substitutes [Blais] as well as aortic stenosis [Burwash], [Otto], severe aortic stenosis [Johnson]	0.0	–	Mean flow rate[24]: 269 ± 53 [Blais]	ml·s^{-1}
022	F_{HLAL_p}	Peak flow rate F_{HLAL}	Exercise in patients with aortic stenosis [Otto]	405.5433	–	512 ± 165 [Kyhl]	ml·s^{-1}
023	F_{HRAR}	Blood flow through the pulmonary valve	Exercise [Wright, 2018]	0.0	–	Mean flow rate: 320 ± 117 [Wright, 2018]	ml·s^{-1}
024	F_{HRAR_p}	Peak flow rate F_{HRAR}	–	514.0842	–	611 ± 182 [Kyhl]	ml·s^{-1}
025	F_{VLHR}	Blood flow through the tricuspid valve [25]	Age, gender [Maceira, 2006b], positive-pressure ventilation [Kyhl]	50.1525	–	–	ml·s^{-1}
026	F_{VLHR_ap}	Active peak F_{VLHR} [25]	Similar to F_{VLHR}	358.6257	–	29 – 770[26] [Maceira, 2006b]	ml·s^{-1}
027	F_{VLHR_ep}	Early peak F_{VLHR} [25]	Similar to F_{VLHR}	494.3659	–	105 – 753[26] [Maceira, 2006b]	ml·s^{-1}
028	F_{VRHL}	Blood flow through the mitral valve [27]	Age, gender [Maceira, 2006a], hypobaric hypoxia [Holloway], positive-pressure ventilation [Kyhl]	34.9546	–	–	ml·s^{-1}
029	F_{VRHL_ap}	Active peak F_{VRHL} [27]	Similar to F_{VRHL}	286.8198	–	77 – 497[26] [Maceira, 2006a]	ml·s^{-1}
030	F_{VRHL_ep}	Early peak F_{VRHL} [27]	Similar to F_{VRHL}	673.2094	–	178 – 1000[26] [Maceira, 2006a]	ml·s^{-1}
031	FF	Filtration fraction	Cardiac dysfunction, secondary to pulmonary hypertension [Damman], early postpartum period [Lafayette], age with hypertension [Bauer], hydropenia with hypertension [Willassen]	0.1551	–	0.19 ± 0.04 [Škrtić]	–

[24] Mean transvalvular flow rate is the quotient of stroke volume and systolic ejection time [Blais].

[25] The rate of the RV filling through the tricuspid valve is characterized by the early peak after the valve opening at early diastole and the active peak at the moment of the right atrial contraction. These peaks can be estimated from the time derivative of the RV volume by cardiovascular magnetic resonance [Maceira, 2006b].

[26] Men and women aged 20 to 59 years.

[27] The rate of the LV filling through the mitral valve in diastole is characterized by two peaks [Caudron], [Boogers], [Zhang, 2019], which can be estimated from the time derivative of the LV volume by cardiovascular magnetic resonance [Maceira, 2006a]. In normal subjects, the LV inflow is greatest immediately after opening of the mitral valve (early peak), the left atrial contraction is responsible for smaller inflow (active peak) [Caudron].

032	G_{AL}	Systemic arterial elastance	Aortic valve stenosis [Laskey, 2009], congestive heart failure secondary to idiopathic dilated cardiomyopathy [Laskey, 1990], diabetes mellitus, hypertension [Haluska], idiopathic dilated cardiomyopathy, coronary artery disease, hypertrophic cardiomyopathy, RV disease [Chemla, 1998], age ≥ 65 years [Fujimoto]	0.9186	–	0.33 – 1.00[28] [Laskey, 1990]	mmHg·ml^{-1}
033	G_{AR}	Pulmonary arterial elastance	Pulmonary hypertension [Thenappan]	0.1873	–	0.08 – 0.26[29] [Thenappan]	mmHg·ml^{-1}
034	G_{VL}	Systemic venous elastance	–	0.0666	–	–	mmHg·ml^{-1}
035	G_{VR}	Pulmonary venous elastance	–	0.0662	–	–	mmHg·ml^{-1}
036	GFR	Glomerular filtration rate	Values of right atrial pressure and renal blood flow [Damman], acute and chronic kidney disease [Levey], [Juretzko], early postpartum period [Lafayette], cirrhosis [Wong]	0.1102	–	0.060 – 0.135 [Levin], [Cachat]	l·min^{-1}
037	gO_2	Oxygen consumption	–	3.8816	–	≈ 4.2 [Treacher]	ml·s^{-1}
038	H	Neurohumoral factor	–	1.3114	1.17 [Proshin MATLAB]	–	s^{-1}
039	$Heart_{Rate}$	Heart rate	Age, gender, race [Ostchega], type 1 diabetes [Škrtić], exercise [Wright, 2018], [Lenz], positive-pressure ventilation [Kyhl]	79.1721	–	60 – 100 [Ostchega]	beats· min^{-1}

[28] Total arterial compliance (TAC) is the inverse of arterial stiffness [Papaioannou], which we used to estimate the values of G_{AL}. We found the following ranges of TAC (in ml/mmHg) obtained by various methods of assessment in control subjects without cardiovascular disease:

- [Haluska], 82 individuals: 1.59 ± 0.50 (pulse-pressure method), 2.26 ± 0.74 (area method), 2.47 ± 0.74 (stroke volume/pulse-pressure method).
- [Fujimoto], stroke volume/pulse-pressure method: 1.8 ± 0.6 (14 individuals aged 21–34 years), 1.8 ± 0.5 (19 individuals aged 35–49 years), 2.0 ± 0.6 (23 individuals aged 50–64 years), 1.2 ± 0.3 (14 individuals with age ≥65 years).
- [Chemla, 1998], 7 individuals: 1.84 ± 0.76 (area method), 1.91 ± 0.76 (stroke volume/pulse-pressure method).
- [Laskey, 1990], 11 individuals: 0.752–1.700·10^{-3} cm^5/dyn (monoexponential aortic diastolic pressure decay), 0.782–2.287·10^{-3} cm^5/dyn (Windkessel model) – taking into account the coefficient 1328 [Gómez] for converting units to ml/mmHg these are ranges 1.00–2.26 and 1.04–3.00 respectively.

Based on these data, we considered the range of 1.00–3.00 ml/mmHg for the total arterial compliance and the range of 0.33 – 1.00 mmHg/мл for G_{AL}.

[29] The range of values of the pulmonary arterial compliance (the inverse of the pulmonary arterial stiffness, which we used to estimate the values of G_{AR}) is 3.8 – 12.0 ml/mmHg [Thenappan].

040	K_{FG}	Filtration coefficient	–	0.0052	–	0.0039–0.0162 [Hoang]	$l \cdot min^{-1} \cdot mmHg^{-1}$
041	K_L	Inotropic factor of the LV	–	0.6250	–	–	–
042	K_R	Inotropic factor of the RV	–	0.6250	–	–	–
043	LA_{PULSE}	Left atrial pulse-wave	–	0.0	–	– –	mmHg
044	M_{sod}	Total exchangeable sodium	Sodium Intake [Brown, 1970], edema due to heart disease [Farber], [Knud], hepatic disease with edema and/or ascites, renal disease with edema [Farber], open intracardiac operations [Pacifico], hypothyroidism [Surveyor]	2831.3985	2850	2040 – 3950 2837±500[30] [Farber]	mEq
045	MAP	Mean arterial pressure	–	90.0275	–	70 – 105 [Doenyas-Barak]	mmHg
046	N_{al}	Normalized aldosterone concentration	–	1.2730	1.0 [Karaaslan, 2005]	–	–
047	N_{als}	Normalized aldosterone secretion rate	–	1.2730	–	–	–
048	nB	Baroreceptor activity	–	0.6607	–	–	–
049	nD	Weariness receptor activity	–	0.1506	–	–	–
050	nH	Myocardial sympathetic inotropic sensitivity	–	0.3316	–	–	–
051	nS	Stress receptor activity	Stress mode [S-D]	0.1780	–	0.0 – 1.0 [Proshin]	–
052	$nSum$	Cardiac center activity	–	1.4041	–	–	–
053	nV	Respiratory receptor activity	–	0.3413	–	–	–
054	*osmolality*	Blood osmolality	–	285.2374	–	275 – 295 [Fogarty]	$mOsm \cdot kg^{-1}$
055	P_{AL}	Systemic arterial pressure	–	89.8166	–	–	mmHg
056	P_{AR}	Pulmonary arterial pressure	–	13.2730	–	–	mmHg
057	P_{AR_D}	Diastolic pulmonary arterial pressure	Cardiomyopathy, coronary artery disease [Pagani], exercise [Wright, 2018]	12.0825	–	4 – 12 [Marini], [Pagani]	mmHg
058	P_{AR_S}	Systolic pulmonary arterial pressure	Cardiomyopathy, coronary artery disease [Pagani], exercise [Wright, 2018]	18.5647	–	15 – 30 [Marini], [Pagani]	mmHg
059	P_D	Diastolic blood pressure	Exhaustive exercise-induced tissue hypoxia [Lenz]	77.9518	80	< 90 [Oparil]	mmHg

[30] The value M_{sod} is determined by the dilution of radioisotopic sodium ^{22}N or ^{24}N. In [Farber], the mean value ± SD is 2896 ± 479 mEq. However, if these values are calculated directly from the data provided by the authors for 27 control individuals, the estimate is 2837 ± 500 mEq.

060	P_{gh}	Glomerular hydrostatic pressure	Type 1 diabetes [Škrtić]	59.1569	–	48.0 – 63.0 [Guberina]	mmHg
061	P_{go}	Glomerular capillary oncotic pressure	Type 1 diabetes [Škrtić], early postpartum period [Lafayette]	28.0322	27.3	27.3 ± 2.6 [Škrtić]	mmHg
062	P_{HL}	LV pressure	–	9.0599	–	–	mmHg
063	P_{HL_D}	LV diastolic pressure	Cardiomyopathy, coronary artery disease [Pagani], diastolic heart dysfunction due to essential hypertension [Stefanadis]	9.0599	–	3 – 12 [Pagani]	mmHg
064	P_{HL_S}	LV systolic pressure	Cardiomyopathy, coronary artery disease [Pagani], diastolic heart dysfunction due to essential hypertension [Stefanadis]	134.9507	130 [Proshin MATLAB]	100 – 140 [Pagani]	mmHg
065	P_{HR}	RV pressure	–	4.8451	–	–	mmHg
066	P_{HR_D}	RV diastolic pressure	Cardiomyopathy, coronary artery disease [Pagani], exercise [Wright, 2018]	4.8451	–	2 – 8 [Pagani]	mmHg
067	P_{HR_S}	RV systolic pressure	Cardiomyopathy, coronary artery disease [Pagani], exercise [Wright, 2018]	20.9558	20	15 – 30 [Pagani]	mmHg
068	P_{ra}	Normalized right atrial pressure	–	0.8620	–	–	mmHg
069	P_S	Systolic blood pressure	Extracellular fluid volume [Faucon], type 1 diabetes and renal hyperfiltration [Škrtić], exhaustive exercise-induced tissue hypoxia [Lenz]	114.1788	120	< 140 [Oparil]	mmHg
070	P_{VL}	Systemic venous pressure	–	5.3039	–	2 – 8[31] [Klingensmith]	mmHg
071	P_{VR}	Pulmonary venous pressure	Cardiomyopathy, coronary artery disease [Pagani]	9.4499	–	3 – 20[32]	mmHg
072	PRA	Plasma renin activity	Type 1 diabetes [Bojestig], [Škrtić], [Valabhji], race [Tu], saline infusion [Ishimitsu], exhaustive exercise-induced tissue hypoxia [Lenz], primary and secondary aldosteronism, hypertension [Glinicki], aldosterone level [Wang]	27.8513	–	15.0 – 31.7 [Valabhji]	fmol·ml^{-1}·min^{-1}

[31] This is the reference range for the central venous pressure (right atrial pressure).

[32] The value of P_{VR} cannot be measured directly. The pulmonary capillary wedge pressure (PCWP) can be used for the indirect estimation. The normal range of PCWP is 2–15 mmHg [Klingensmith]. The mean value of P_{VR} is intermediate between the mean PCWP and the mean value of P_{AR}, and is approximately 30% higher than the PCWP [Chaliki] (shown for dogs). Therefore, we assumed 3 – 20 mmHg as the range for P_{VR}.

073	PRC	Plasma renin concentration	Race [Tu], sodium intake [Wambach], primary and secondary aldosteronism, hypertension [Glinicki], chronic kidney disease [Juretzko]	27.3948	PRC_{nom}	3.42 – 69.4 [Perschel]	pg·ml^{-1}
074	R_a	Resistance of afferent vessels (arteries and arterioles)	–	26.0578	–	–	mmHg·min·ml^{-1}
075	R_{a_dyne}	R_a in dyn·s·sm^{-5}	Type 1 diabetes [Škrtić]	2076.2867	–	1555 ± 635 [App. B]	dyn·s·sm^{-5}
076	R_{aa}	Single-afferent arteriole resistance	–	1.7746E7	–	–	mmHg·min·l^{-1}
077	R_{aa_0}	Nominal single-afferent arteriole resistance	–	0.9317E7	–	–	mmHg·min·l^{-1}
078	R_e	Resistance of all efferent arterioles	–	32.9348	–	–	mmHg·min·ml^{-1}
079	R_{e_dyne}	R_e in dyn·s·sm^{-5}	–	2624.2428	–	2270 ± 364 [App. B]	dyn·s·sm^{-5}
080	R_{ea}	Single-efferent arteriole resistance	–	9.0571E7	–	–	mmHg·min·l^{-1}
081	R_{ea_0}	Nominal single-efferent arteriole resistance	–	4.0073E7	–	–	mmHg·min·l^{-1}
082	$R_{preglom}$	Resistance of interlobar, arcuate, and interlobular arteries	–	19.6049	–	10.0 – 20.0 [Hallow, 2017a]	mmHg·min·l^{-1}
083	R_{sec}	Renin secretion rate	–	1.5824	–	–	–
084	RA_{PULSE}	Right atrial pulse-wave	–	0.0	–	–	mmHg
085	RBF	Renal blood flow	Type 1 diabetes and renal hyperfiltration [Cherney], [Škrtić], hydropenia with hypertension [Willassen], cardiac dysfunction, secondary to pulmonary hypertension [Damman]	1.1847	–	0.623 – 1.730 [Bax]	l·min^{-1}
086	RPF	Renal plasma flow	Cardiac dysfunction, secondary to pulmonary hypertension [Damman], cirrhosis [Wong], type 1 diabetes and renal hyperfiltration [Cherney], [Škrtić]	0.7108	–	0.628 ± 0.162 [Škrtić]	l·min^{-1}
087	$RSNA$	Renal sympathetic nerve activity	–	1.2179	–	–	–
088	RVR	Renal vascular resistance	Type 1 diabetes and renal hyperfiltration [Cherney], [Škrtić], hypertension, age with hypertension [Bauer], cirrhosis [Wong]	70.9276	–	55.1 – 83.6 [App. B]	mmHg·min·l^{-1}

089	*SV*	LV stroke volume	Age [Cain], hypertension, exercise in hypertensive [Cléroux] and healthy individuals [Wright, 2018], hypobaric hypoxia [Holloway], positive-pressure ventilation [Kyhl]	62.5264	83.0	39.1 – 115.3 (age 18+) [Cattermole]	ml
090	*Systole*	Total actual systole indicator	–	0.0	–	–	–
091	$Systole_L$	Actual LV systole indicator	–	0.0	1.0 [Proshin MATLAB]	–	–
092	$Systole_{L\_Exp}$	Nominal LV systole indicator	–	0.0	–	–	–
093	$Systole_{Length\_L}$	Actual LV systole duration	–	0.2642	–	–	s
094	$Systole_{Length\_L\_Exp}$	Nominal LV systole duration	–	0.2642	–	–	s
095	$Systole_{Length\_R}$	Actual RV systole duration	–	0.2642	–	–	s
096	$Systole_{Length\_R\_Exp}$	Nominal RV systole duration	–	0.2642	–	–	s
097	$Systole_R$	Actual RV systole indicator	–	0.0	1.0 [Proshin MATLAB]	–	–
098	$Systole_{R\_Exp}$	Nominal RV systole indicator	–	0.0	–	–	–
099	*TBW*	Total body water[33]	Weight, height, age, gender [Skrabal, 1973], [Deurenberg], hypothyroid [Surveyor]	37.7037	40.0	24.45 – 56.63[34] [Hoffer]	l
100	*TPR*	Total peripheral resistance	Hypertension, exercise [Cléroux]	18.1861	–	12.5 – 22.5 [Daly]	$mmHg \cdot min \cdot l^{-1}$
101	*V*	Total blood volume[35]	Weight, height [Feldschuh]	4853.9658	–	3061 – 6092 [Wennesland]	ml
102	V_{AL}	Systemic arterial blood volume	–	612.5806	$0.13 \cdot V$ [Hall]	–	ml

[33] Tritium oxide (^3H_2O, T_2O), deuterium oxide (^2H_2O, D_2O) and antipyrine are markers for measuring the total body water [Hall]. D_2O gives a fairly accurate estimate [Moore, 1946] (tested in rabbits). Measurement with antipyrine is associated with a larger error [Ljunggren]. ^3H_2O gives the same water volume as D_2O [Prentice]. In addition to methods associated with the injection of these markers into the organism, non-invasive methods can also be used: Dual-energy X-ray absorptiometry, air displacement plethysmography, nuclear magnetic resonance spectroscopy, and bioelectrical impedance analysis [Roumelioti]. The result of the latter correlates well with the ^3H_2O space [Hoffer], but on average gives a slightly less value than the D_2O space [Smith], [Raimann].

[34] The following *TBW* estimates obtained in healthy individuals were found in the literature: 37.09 – 56.63 (20 individuals, tritium) [Hoffer], 35.82 – 51.53 (18 men, deuterium), 24.45 – 35.47 (14 women, deuterium) [Pichler]. The mean *TBW* values for different men and women age groups are given in [Chumlea].

[35] The indicator for measuring blood volume is Cr-labeled red blood cells, or this volume can be calculated as Plasma volume/(1 − Hematocrit) [Hall]. The latter formula is used, for example, in [Gibson], where plasma volume is determined using the dye T-1824 (Evans blue) administered intravenously. The corresponding estimate for the blood volume is 2990 – 6980 ml.

103	V_{AR}	Pulmonary arterial blood volume	–	157.6286	$0.035 \cdot V$[36] [Gazioglu]	–	ml
104	V_{ecf}	Extracellular fluid volume[37]	Weight, height, age, gender [Deurenberg], open intracardiac operations [Pacifico]	16.6504	–	–	l
105	V_{HL}	LV blood volume	–	88.7840	$0.03 \cdot V$	–	ml
106	V_{HL_KD}	LV end-diastolic volume	Age, gender [Maceira, 2006a], [Cain], [Hudsmith], positive-pressure ventilation [Kyhl]	100.4978	140.0 [Proshin MATLAB]	M: 67 – 155<br>W: 56 – 104<br>[Lang]	ml
107	V_{HL_KS}	LV end-systolic volume	Age, gender [Maceira, 2006a], [Cain], [Hudsmith], positive-pressure ventilation [Kyhl]	37.9714	–	M: 22 – 58<br>W: 19 – 49<br>[Lang]	ml
108	V_{HR}	RV blood volume	–	83.0649	$0.03 \cdot V$	–	ml
109	V_{HR_KD}	RV end-diastolic volume	Age, gender [Hudsmith], [Maceira, 2006b], positive-pressure ventilation [Kyhl]	100.4978	140.0 [Proshin MATLAB]	M: 124 – 256<br>W: 78 – 218<br>[Hudsmith]	ml
110	V_{HR_KS}	RV end-systolic volume	Age, gender [Hudsmith], [Maceira, 2006b], positive-pressure ventilation [Kyhl]	37.9714	–	M: 38 – 118<br>W: 20 – 92<br>[Hudsmith]	ml
111	V_{VL}	Systemic venous blood volume	–	3493.4664	$0.71 \cdot V$[38] [Hall], [Magder]	~ 2000 – 3500 [Hall]	ml
112	V_{VR}	Pulmonary venous blood volume	–	418.4412	$0.065 \cdot V$[39] [Gazioglu]	–	ml
113	$V_{AR} + V_{VR}$	Pulmonary blood volume	–	576.0699	–	~ $0.09 \cdot V – 0.10 \cdot V$ [Hall], [Gazioglu]	ml
114	$V_{AR} + V_{VR} + V_{HR} + V_{HL}$	Cardiopulmonary blood volume	–	747.9188	–	~ $0.153 \cdot V$ [Levinson]	ml
115	VO_2	Venous oxygen content	–	0.1369	0.15 [Proshin MATLAB]	0.095 – 0.168 [Hattori]	–

[36] 35% of the value $V_{AR} + V_{VR}$.

[37] There are substantial differences in estimates of the extracellular fluid volume V_{ecf} depending on the exogenous markers (usually radioactive compounds) used for the measurement [Roumelioti]. The use of radiosulfate in [Walser] gives the range of 7.6 – 15.9 liters. The use of radiobromine in [Tarazi] leads to the range of 13.9 – 21.4 liters. The estimation of V_{ecf} can be also performed by the modern methods [Roumelioti]: evaluation of body composition (e.g. bioimpedance analysis [Dou], [Raimann]); MRI; simultaneous measurement of *TBW* and potassium; estimation of *GFR* using exogenous markers with extracellular distribution, etc.

[38] 64% are from veins, 7% are from arterioles and capillaries.

[39] 45% of the value $V_{AR} + V_{VR}$ are from veins, 20% are from capillaries.

116	Y_{ALVL}	Conductivity of systemic microvessels (venules, capillaries and arterioles)	–	1.5989	–	–	$ml \cdot s^{-1} \cdot mmHg^{-1}$
117	Y_{ARVR}	Conductivity of pulmonary microvessels (venules, capillaries and arterioles)	–	15.2889	–	–	$ml \cdot s^{-1} \cdot mmHg^{-1}$
118	Y_{VLHR}	Conductivity of the tricuspid valve and systemic veins	–	109.3040	–	–	$ml \cdot s^{-1} \cdot mmHg^{-1}$
119	Y_{VRHL}	Conductivity of the mitral valve and pulmonary veins	–	89.6326	–	–	$ml \cdot s^{-1} \cdot mmHg^{-1}$
120	$\frac{1}{Y_{HLAL}}+\frac{1}{Y_{ALVL}}+\frac{1}{Y_{VLHR}}$	Systemic vascular resistance (can be estimated by total peripheral resistance *TPR*)	Aortic valve stenosis [Laskey, 2009], congestive heart failure secondary to idiopathic dilated cardiomyopathy [Laskey, 1990], cardiomyopathy, coronary artery disease [Pagani], diastolic heart dysfunction due to essential hypertension [Stefanadis]	0.7774	–	0.5271 – 1.2048[40] [Klingensmith]	$s \cdot mmHg \cdot ml^{-1}$
121	$\frac{1}{Y_{HRAR}}+\frac{1}{Y_{ARVR}}+\frac{1}{Y_{VRHL}}$	Pulmonary vascular resistance	Mitral or aortic valve disease [Gazioglu], cardiomyopathy, coronary artery disease [Pagani], diastolic heart dysfunction due to essential hypertension [Stefanadis], exercise [Wright, 2018], pulmonary hypertension [Gan]	0.0941	–	0.0151 – 0.0979[41] [Klingensmith]	$s \cdot mmHg \cdot ml^{-1}$
122	α_{map}	Effect of mean arterial pressure on renal sympathetic nerve activity	–	1.2264	–	–	–
123	α_{rap}	Effect of right atrial pressure on renal sympathetic nerve activity	–	0.9931	–	–	–
124	β_{rsna}	Effect of renal sympathetic nerve activity on afferent arteriole resistance and resistance of interlobar, arcuate, and interlobular arteries	–	1.3269	–	–	–

[40] 700–1600 $dyn \cdot s \cdot cm^{-5}$ [Klingensmith] using the factor 1/1328 for the units conversion to $s \cdot mmHg \cdot ml^{-1}$ [Gómez].
[41] 20–130 $dyn \cdot s \cdot cm^{-5}$ [Klingensmith] using the factor 1/1328 for the units conversion to $s \cdot mmHg \cdot ml^{-1}$ [Gómez].

125	γ_{at}	Effect of AT1-bound angiotensin II on $\eta_{pt\_sodreab}$	–	0.9973	–	–	–
126	γ_{filsod}	Effect of filtered sodium load on $\eta_{pt\_sodreab}$	–	1.0876	–	–	–
127	γ_{rsna}	Effect of renal sympathetic nerve activity on $\eta_{pt\_sodreab}$	–	1.0114	–	–	–
128	$\eta_{cd\_sodreab}$	Fractional collecting duct sodium reabsorption	–	0.9404	–	–	–
129	$\eta_{dt\_sodreab}$	Fractional distal tubule sodium reabsorption	–	0.5093	–	–	–
130	$\eta_{pt\_sodreab}$	Fractional sodium reabsorption in the proximal tubule and the loop of Henle	Cirrhosis [Simón], race [Bochud], type 1 diabetes [Hannedouche], acute ischemic renal failure [Suzuki]	0.8776	–	M: 0.795 ± 0.083<br>W: 0.793 ± 0.084<br>[Seidlerová]	–
131	$\eta_{dt\_sodreab} + \eta_{cd\_sodreab} - \eta_{dt\_sodreab} \cdot \eta_{cd\_sodreab}$	Fractional sodium reabsorption in the distal tubule and subsequent parts of the nephron	Race [Bochud], type 1 diabetes [Hannedouche], insulin infusion [Skøtt], acute ischemic renal failure [Suzuki]	0.9707	–	M: 0.946 ± 0.032<br>W: 0.950 ± 0.031<br>[Seidlerová]	–
132	λ_{anp}	Effect of atriopeptin on $n_{cd\_sodreab}$	–	0.9984	–	–	–
133	λ_{dt}	Effect of distal tubule sodium outflow on $n_{cd\_sodreab}$	–	1.0465	–	–	–
134	μ_{adh}	Effect of vasopressin concentration on tubular water reabsorption rate	–	0.8903	–	–	–
135	ν_{AT1_ANGII}	Effect of AT1-bound angiotensin II on renin secretion rate	–	1.0424	–	–	–
136	ν_{MD_sod}	Effect of macula densa sodium flow on renin secretion rate	–	1.1425	–	–	–
137	ν_{RSNA}	Effect of renal sympathetic nerve activity on renin secretion rate	–	1.1501	–	–	–
138	ξ_{at}	Effect of angiotensin hormone on aldosterone secretion rate	–	1.2730	–	–	–
139	ξ_{k_sod}	Effect of potassium concentration on aldosterone secretion rate	–	1.0000	–	–	–

140	Σ_{myo}	Myogenic autoregulation signal	–	0.9883	1.0	–	–
141	Σ_{tgf}	Tubuloglomerular feedback signal	–	0.6459	1.0 [Karaaslan 2005]	–	–
142	$\Phi_{cd\_sodreab}$	Absolute collecting duct sodium reabsorption rate	–	0.8911	–	–	$mEq \cdot min^{-1}$
143	Φ_{dt_sod}	Distal tubule sodium outflow	–	0.9476	–	–	$mEq \cdot min^{-1}$
144	$\Phi_{dt\_sodreab}$	Absolute distal tubule sodium reabsorption rate	–	0.9836	–	–	$mEq \cdot min^{-1}$
145	Φ_{filsod}	Amount of sodium filtered from the glomerulus to the proximal tubule per unit time (filtered sodium load)	Cirrhosis [Wong], acute ischemic renal failure [Suzuki]	15.7808	–	13.0 – 19.0[42] [Natarajan], [Hannedouche], [Boer]	$mEq \cdot min^{-1}$
146	Φ_{md_sod}	Macula densa sodium flow	–	1.9312	–	–	$mEq \cdot min^{-1}$
147	$\Phi_{pt\_sodreab}$	Absolute proximal tubular sodium reabsorption rate	–	13.8496	–	–	$mEq \cdot min^{-1}$
148	Φ_{t_wreab}	Tubular water reabsorption rate	–	0.1088	–	–	$l \cdot min^{-1}$
149	Φ_{u}	Urine flow rate	Type 1 diabetes and renal hyperfiltration [Cherney]	0.0015	–	0.0011 ± 0.0005 (1.65 ± 0.70 l/day) [Malisova]	$l \cdot min^{-1}$
150	Φ_{u_sod}	Urine sodium flow	Essential hypertension [Cowley], [Ferrario], salt intake [Ishimitsu], extracellular fluid volume [Faucon], type 1 diabetes and renal hyperfiltration [Cherney]	0.0565	–	0.097 ± 0.049 (140 ± 70 mEq/day) [Letcher]	$mEq \cdot min^{-1}$
151	Φ_{win}	Water intake	–	0.0015	–	0.0019 ± 0.0007 (2.75 ± 1.01 l/day) [Malisova]	$l \cdot min^{-1}$
152	Ψ_{al}	Effect of aldosterone concentration on fractional distal tubule sodium reabsorption	–	1.0186	–	–	–

[42] The ranges for healthy individuals (mmol/min): 15.4 ± 2.0 (low-salt diet), 17.1 ± 2.4 (high-salt diet) [Natarajan]; 18.11 ± 1.88 [Hannedouche], 15.7±2.1 [Boer].

153	Ψ_{AT1_aa}	Effect of AT1-bound angiotensin II on afferent arteriole resistance	–	2.2484	–	–	–
154	Ψ_{AT1_ALVL}	Effect of AT1-bound angiotensin II on blood flow through the systemic microvessels	–	1.7702	–	–	–
155	Ψ_{AT1_Baro}	Effect of AT1-bound angiotensin II on baroreceptor activity	–	0.9882	–	–	–
156	Ψ_{AT1_ea}	Effect of AT1-bound angiotensin II on efferent arteriole resistance	–	2.2601	–	–	–
157	$\Psi_{AT1\_preglom}$	Effect of AT1-bound angiotensin II on resistance of interlobar, arcuate, and interlobular arteries	–	1.4949	–	–	–
158	Ψ_{AT1_Stress}	Effect of AT1-bound angiotensin II on stress receptor activity	–	0.9954	–	–	–
159	ω_{AL}	Unstressed volume of systemic arteries	–	514.8097	–	–	ml
160	ω_{AR}	Unstressed volume of pulmonary arteries	–	86.7566	–	–	ml

APPENDIX B. DERIVATION OF THE FORMULA FOR THE RENAL BLOOD FLOW CALCULATION

The following vessels define the total renal vascular resistance *RVR* [Hall]:

A. interlobar, arcuate, and interlobular arteries;
B. afferent arterioles;
C. glomerular capillaries;
D. efferent arterioles;
E. peritubular capillaries;
F. interlobar, interlobular, and arcuate veins.

Table B1. Determination of the renal vascular resistance as the sum of resistances of separate vessels. The column "Clinical measurements" indicates whether the corresponding study gives a clinical estimation of these resistances in healthy individuals or not.

<table>
<tr><th rowspan="2">Reference</th><th rowspan="2">Clinical measurements</th><th colspan="6">Vessels</th></tr>
<tr><th>A</th><th>B</th><th>C</th><th>D</th><th>E</th><th>F</th></tr>
<tr><td>Karaaslan, 2005</td><td>–</td><td>–</td><td>+</td><td>–</td><td>+</td><td>–</td><td>–</td></tr>
<tr><td>Karaaslan, 2014</td><td>–</td><td>–</td><td>+</td><td>–</td><td>+</td><td>–</td><td>–</td></tr>
<tr><td>Hallow, 2014</td><td>–</td><td>+</td><td>+</td><td>–</td><td>+</td><td>–</td><td>–</td></tr>
<tr><td>Hallow, 2017a</td><td>–</td><td>+</td><td>+</td><td>–</td><td>+</td><td>+</td><td>–</td></tr>
<tr><td>Gómez, 1951</td><td>+</td><td colspan="2">+</td><td colspan="2">+</td><td colspan="2">+</td></tr>
<tr><td>Škrtić, 2015</td><td>+</td><td colspan="2">+</td><td colspan="2">+</td><td colspan="2">+</td></tr>
</table>

According to [Gómez], the *RVR* value, defined as the ratio of the difference between mean arterial pressure (*MAP*) and renal venous pressure P_v to renal blood flow (*RBF*), can be represented as the sum of three components:

$$RVR = \frac{MAP - P_v}{RBF} = R_a + \frac{RBF - GFR}{RBF} \cdot R_e + R_v, \quad \textbf{(1)}$$

where R_a is the resistance of afferent vessels (arteries and arterioles), R_e denotes the resistance of efferent arterioles, R_v corresponds to the resistance of renal veins, *GFR* is the glomerular filtration rate, and the factor $(RBF - GFR)/RBF$ characterizes the contribution to *RVR* of the glomerular filtrate which is reabsorbed by the peritubular capillaries. To determine R_a, R_e and R_v, the mean pressure values in glomerular (P_{gh}) and peritubular (P_t) capillaries are used:

$$R_a = \frac{MAP - P_{gh}}{RBF}, \; R_e = \frac{P_{gh} - P_t}{RBF - GFR}, \; R_v = \frac{P_t - P_v}{RBF}. \quad \textbf{(2)}$$

Thus, the resistances of these capillaries are respectively subsumed in the calculated values of R_a and R_e, as well as R_e and R_v.

The *GFR* value is defined depending on the hydrostatic pressure in the Bowman's space P_B and the oncotic pressure P_{go} with the constant $K_{FG} = 0.0867$ ml·s^{-1}·mmHg^{-1} [Škrtić]:

$$GFR = K_{FG} \cdot \left(P_{gh} - P_B - P_{go}\right). \quad \textbf{(3)}$$

The study [Gómez] makes the assumption that $P_t \approx P_B + P_{go}$, which in the combination with (2) and (3) gives the following equation for R_e:

$$R_e = \frac{GFR}{K_{FG} \cdot \left(RBF - GFR\right)}. \quad \textbf{(4)}$$

Since later studies showed that this assumption is incorrect [Digne-Malcolm], we did not use the data obtained by the formula (4) in articles [Gómez] and [Škrtić] as reference values. Instead, we

estimated R_a, R_e, and R_v using formulas (2) and (3) with constants $P_{go} = 25$ mmHg, $P_B = 10$ mmHg [Gómez], $P_t = 25$ mmHg, and $P_v = 6$ mmHg [Digne-Malcolm] for renal hemodynamic parameters in 8 healthy volunteers [van der Bel]. We used the value 1328 [Gómez] as the conversion factor of *RVR* units from mmHg·s·ml^{-1} to dyn·s·cm^{-5}. The estimation results are shown in Table B2.

Table B2. Estimation of R_a, R_e and R_v using parameters of renal hemodynamics in 8 healthy volunteers [van der Bel].

№	van der Bel *et al*., 2016			Design parameters				
	MAP **mmHg**	***GFR*** **ml/min**	***RBF*** **ml/min**	P_{gh} **mmHg**	R_a **dyn·s/cm**^{5}	R_e **dyn·s/cm**^{5}	R_v **dyn·s/cm**^{5}	***RVR*** **mmHg·min/l**
1	88	124	1257.1	58.84	1848	2379	1204	65.2
2	85	104	944.4	54.99	2532	2843	1603	83.6
3	80	138	1333.3	61.53	1104	2435	1135	55.5
4	84	151	1680.0	64.03	947	2034	901	46.4
5	74	101	1233.3	54.42	1265	2070	1228	55.1
6	86	110	1075.0	56.15	2213	2572	1408	74.4
7	83	89	1383.3	52.11	1779	1669	1094	55.7
8	78	147	1560.0	63.26	753	2157	970	46.2
mean	82	121	1308.3	58.16	1555	2270	1193	60.3
SD	5	23	239.9	4.42	635	364	228	13.3

From the formulas (1) and (2), we find the equations for *RBF* and P_{gh}:

$$RBF = \frac{MAP - P_v + GFR \cdot R_e}{R_a + R_e + R_v}, \qquad P_{gh} = MAP - RBF \cdot R_a .$$

Taking into account the formula (3), we obtain:

$$RBF = \frac{MAP - P_v + K_{FG} \cdot R_e \cdot \left(MAP - P_B - P_{go} \right)}{R_a + R_e + R_v + K_{FG} \cdot R_e \cdot R_a} .$$

When modeling a healthy person, we assume that R_v is the constant equal to the mean normal value of 1193 dyn·s·cm^{-5} ≈ 15 mmHg·min·l^{-1} (Table B2).

APPENDIX C. MODELING THE PATIENTS WITH CARDIOVASCULAR DISEASES

Table C1. List of the fitted parameters with normal and abnormal search intervals used to generate equilibrium states corresponding to patients with cardiovascular diseases.

№	Parameter, notation in the model	Ranges/values		Units	Justification of ranges
		Normal	Abnormal		
01	Total metabolic intensity, A_1	0.00032 – 0.00128	–	ml^{-1}	The normal initial value A_1 = 0.0008 ml^{-1} with deviation of 60%, which is the allowable fluctuation in basal metabolic rate (closely related to oxygen consumption) in people with the same weight [Henry].
02	Sympathetic sensitivity of systemic microvessels, A_3	0.1	Hypertension 0.1 – 0.6	ml·mmHg^{-1}	Parameter A_3 is used to calculate conductivity of systemic microvessels Y_{ALVL}, which contributes most to systemic vascular resistance $SVR = 1/Y_{HLAL} + 1/Y_{ALVL} + 1/Y_{VLHR}$. An increase of A_3 to 0.6 ml·mmHg^{-1} without changing the other parameters leads in the model to growing *SVR* to 1.5 s·mmHg·ml^{-1}, the typical value in patients with hypertension [Stefanadis], [Prys-Roberts].
03	Sympathetic sensitivity of pulmonary microvessels, A_{13}	0.65	PH 0.65 – 3.90	ml·mmHg^{-1}	Pulmonary hypertension (PH) is related to the chronic elevation of pulmonary vascular resistance (*PVR*) [Chemla, 2002]. One way to enlarge *PVR* in the model (as in the case of *SVR*) is the increase of the A_{13} value. By analogy with A_3, we considered a possible change in A_{13} up to 6 times of the normal value.
04	Systemic arterial tone, A_9	0.07	Hypertension 0.07 – 0.09	mmHg· s·ml^{-1}	Sustained increases in arterial tone are an essential component in the development of hypertension [Amberg]. Thus, we assumed an increase in the normal value of A_9 up to 25%.
05	Pulmonary arterial tone, A_{19}	0.02	PH 0.02 – 0.04	mmHg· s·ml^{-1}	By analogy with the parameter A_9, we assumed a possible increase in the normal value of A_{19} in patients with PH.
06	Normal vasopressin level, C_{adh_norm}	1.0 – 13.3	–	pg·ml^{-1}	The normal range [Yarmohammadi].
07	Normal aldosterone level, C_{al_norm}	70 – 300	–	pg·ml^{-1}	The normal range [Fischbach].
08	Normal atriopeptin level, C_{anp_norm}	7.4 – 152.0	–	ng·l^{-1}	The normal ranges: 27–152 ng·l^{-1} [Nozuki], 7.4–15.7 ng·l^{-1} [Cannone].
09	Oxygen capacity of hemoglobin, C_H	1.32 – 1.39	–	ml·g^{-1}	The normal range [Dijkhuizen].
10	Serum potassium, C_K	3.5 – 5.5	–	mEq·l^{-1}	The normal range [Rastegar].
11	Afferent arteriole diameter, d_{aa}	8.7 – 23.9	–	μm	Neal et al. estimated the value of d_{aa} for normal human glomeruli at physiological hydrostatic and oncotic pressures at 21.5 ± 2.4 μm [Neal]. Hill et al. give values of 15.7 ± 4.9 μm for hypertensive patients with proteinuria and/or azotemia, with no evidence of other renal disease, and 13.4 ± 4.7 μm for normotensive nondiabetic patients, aged 37 – 85 years [Hill].

12	Efferent arteriole diameter, d_{ea}	12.2 – 20.1	–	μm	The value of d_{ea} was estimated at 15.9 ± 2.4 μm [Neal]. We assumed a possible deviation from this range by 10%.
13	Action threshold of the Frank-Starling law, $FS_{threshold0}$	0.0 – 40.0	–	ml	Parameter $FS_{threshold0}$ is an auxiliary characteristic describing the pump function of the ventricle. The range was taken from the application "Physiological Avatar" (version 2020.9.14.0) on the website https://physiological-avatar.azurewebsites.net/index.aspx.
14	Basic systemic arterial elastance, G_{AL0}	0.33 – 1.00	Hypertension 0.33 – 1.67 CHF, LVH 0.33 – 2.38 PH 0.71 – 1.43	mmHg·ml^{-1}	The normal range of systemic arterial compliance (SAC), the inverse of systemic arterial stiffness [Papaioannou], used for G_{AL0} evaluation, is 1.00 – 3.00 ml·mmHg^{-1} (Table A4). In hypertensive patients, SAC is reduced on average by 40% [Haluska]. SAC in patients with CHF: 1.2 – 1.7 ml·mmHg^{-1} in low *PVR* (<200 dyn·s·cm^{-5}), and 0.7 – 1.4 ml·mmHg^{-1} in high *PVR* [Melenovsky]. In healthy subjects and patients with miscellaneous cardiac diseases SAC is 0.42 – 2.63 ml·mmHg^{-1} [Chemla, 2017].
15	Basic pulmonary arterial elastance, G_{AR0}	0.08 – 0.26	CHF 0.08 – 0.50 PH 0.26 – 2.50	mmHg·ml^{-1}	The normal range of pulmonary arterial compliance (PAC), the inverse of pulmonary arterial stiffness, used for the G_{AR0} evaluation, is 3.8 – 12.0 ml·mmHg^{-1} in normal subjects, and 0.4 – 3.8 ml·mmHg^{-1} in patients with PH [Thenappan]. PAC in patients with heart failure: 2.0 – 3.3 ml·mmHg^{-1} in low *PVR* (<200 dyn·s·cm^{-5}), 0.8 – 1.5 ml·mmHg^{-1} in high *PVR* [Melenovsky].
16	LV wall elasticity, G_{HL}	0.01 – 0.43	Hypertension 0.02 – 0.72	mmHg·ml^{-1}	The normal range of G_{HL} in the relaxation phase in subjects without cardiovascular disease is 0.01–0.43 (0.16 ± 0.11) mmHg·ml^{-1} [Zhang, 2008]. In hypertensive patients, the LV wall compliance (the inverse of stiffness [Zhang, 2008]) is decreased by about 40% [Safar, 1985]. The range of G_{HR} values is taken similarly to G_{HL}.
17	RV wall elasticity, G_{HR}				
18	Basic elasticity of systemic veins, G_{VL0}	0.01 – 0.05	–	mmHg·ml^{-1}	The normal value of G_{VL0} by the [Proshin] model is 0.0375 mmHg·ml^{-1}. According to our estimates for a healthy person, this value is 0.0108 mmHg·ml^{-1}. As the normal range of variations, we assumed the interval 0.01 – 0.05 mmHg·ml^{-1}. The normal range of G_{VR0} is done similarly to G_{VL0}.
19	Basic elasticity of pulmonary veins, G_{VR0}				
20	Plasma glucose, *glucose*	3.9 – 6.1	–	mmol·l^{-1}	The normal range [Dedov].
21	Hematocrit, *Hct*	M: 40 – 54 W: 36 – 48	–	%	The normal range [Billett] for mens (M) and womens (W).
22	Hemoglobin, *He*	M: 140 – 180 W: 120 – 160	–	g·l^{-1}	The normal range [Billett] for mens (M) and womens (W).
23	Baroreceptor sensitivity, $Heart_{Baro}$	0.60	Atherosclerosis 0.45 – 0.60	–	The baroreflex decreases with carotid and coronary atherosclerosis [Ziegler]. Therefore, we assumed a possible decrease in the $Heart_{Baro}$ value by 25% for this group of patients.
24	Cardiac center basic activity, $Heart_{Base}$	0.01 – 1.00	–	–	The fitted normal value of $Heart_{Base}$ is given in Table A3. As a range of values we assumed the interval from 0.01 to 1.00.

25	The ω_{AL}/V_{AL} ratio, k_{AL}	0.7 – 1.0	–	–	A stressed component of blood volume, which determines flow, is about 30% [Magder]. Thus, as the range of k_{AL}, k_{AR}, k_{VL}, and k_{VR} we considered the interval from 0.7 to 1.0.
26	The ω_{AR}/V_{AR} ratio, k_{AR}				
27	The ω_{VL}/V_{VL} ratio, k_{VL}				
28	The ω_{VR}/V_{VR} ratio, k_{VR}				
29	The ω_{HL}/V_{HL} ratio, k_{HL}	0.0 – 0.3	CHF<br>0.0 – 0.7	–	The normal range is explained in Table A3. As the range in patients with heart diseases we considered the interval 0.0 – 0.7.
30	The ω_{HR}/V_{HR} ratio, k_{HR}				
31	Normal filtration coefficient, K_{FG_0}	0.0039 – 0.0162	–	$l \cdot min^{-1} \cdot mmHg^{-1}$	The normal range estimated in healthy volunteers [Hoang].
32	Aortic regurgitation (AR) coefficient, K_{HLAL}	0.0	AR: 0.0 – 0.3	–	The normal value of coefficients is 0.0. In patients with some valve regurgitation, the corresponding parameter takes values 0.0 – 0.3 [S-D]. We divided this range depending on the regurgitation stage: 0.0 – 0.1 (mild), 0.1 – 0.2 (moderate), 0.2 – 0.3 (severe).
33	Pulmonary regurgitation (PR) coefficient, K_{HRAR}		PR: 0.0 – 0.3		
34	Tricuspid regurgitation (TR) coefficient, K_{VLHR}		TR: 0.0 – 0.3		
35	Mitral regurgitation (MR) coefficient, K_{VRHL}		MR: 0.0 – 0.3		
36	LV inotropic status, K_{L0}	0.5 – 0.8	CHF, LVH<br>0.4 – 0.8<br>PH<br>0.2 – 0.8	–	The normal range and the range in patients with chronic heart failure (CHF) are given in [Solodyannikov]. We used the same range as for CHF in patients with LV hypertrophy (LVH). Parameter fitting of the model to physiology values of patients with PH and the reduced value (about 25%) of ejection fraction [Wright, 2017] gives values of 0.2 – 0.3 for K_{L0} and K_{R0}.
37	RV inotropic status, K_{R0}				
38	Afferent arteriole length, L_{aa}	101 – 123	–	µm	The normal value 112 µm of L_{aa} is taken from [Neal]. As a possible range of fluctuations, we assumed the deviation from this value by 10%.
39	Efferent arteriole length, L_{ea}	124 – 152	–	µm	The normal value 138 µm of L_{ea} is taken from [Neal]. As a possible range of fluctuations, we assumed the deviation from this value by 10%.
40	Initial value of total exchangeable sodium, $M_{sod}(0)$	2040 – 3950	–	mEq	The initial value range of M_{sod} in healthy humans is taken from the work [Farber].
41	Number of nephrons in the kidneys, $N_{nephrons}$	1.50E6 – 3.00E6	Hypertension<br>0.80E6 – 2.75E6	–	The average number of glomeruli in one human kidney is 895,711, with an overall 12.8-fold range from 210,332 to 2,702,079 [Bertram]. Our estimate of the normal value of two kidneys in a healthy person is $N_{nephrons}$ = 2.75E6 (Table A3). Patients with hypertension could have 30% fewer glomeruli than individuals without this disease, with an average of approximately 600,000 glomeruli per kidney [Hoy]. As the lower bound of $N_{nephrons}$, we took this value multiplied by two and reduced by the standard deviation given in the study [Hoy].

42	Normal fractional distal tubule sodium reabsorption, n_{ε_dt}	0.3 – 0.7	–	–	As the normal range of n_{ε_dt} we considered the interval from 0.3 to 0.7. This parameter is fitted so that the value $n_{\varepsilon\_dt} + n_{\eta\_cd} - n_{\eta\_cd} \cdot n_{\varepsilon\_dt}$ remains within the normal range 0.78 – 0.98 (Table C2).
43	Normal fractional collecting duct sodium reabsorption, n_{η_cd}	0.6 – 1.0	–	–	As the normal range of n_{η_cd}, we considered the interval from 0.6 to 1.0. This parameter is fitted so that the value $n_{\varepsilon\_dt} + n_{\eta\_cd} - n_{\eta\_cd} \cdot n_{\varepsilon\_dt}$ remains within the normal range 0.78 – 0.98 (Table C2).
44	Normal fractional proximal sodium reabsorption, n_{η_pt}	0.67 – 0.97	–	–	The normal estimates: 0.795 ± 0.083 (mans), 0.793 ± 0.084 (womans) [Seidlerová], 0.77 ± 0.09 (young), 0.87 ± 0.06 (elderly) [Fliser]. Randomly recruited 95 nuclear families of black South African ancestry: 0.908 – 0.958, and 103 nuclear families of white Belgian ancestry: 0.759 – 0.863 [Bochud]. Randomly recruited 317 untreated subjects from a white population: 0.892 ± 0.054 in low and 0.903 ± 0.051 in high sodium excretion [Jin].
45	Hydrostatic pressure in the Bowman's space, P_B	10 – 15	–	mmHg	The normal range [Digne-Malcolm].
46	Glomerular hydrostatic pressure, P_{gh_norm}	48.0 – 63.0	–	mmHg	The normal estimates: 59.6 ± 2.7 mmHg [Škrtić], 54.3 ± 4.5 [Tsuda], 48.0 – 63.0 [Guberina].
47	Initial value of glomerular capillary oncotic pressure, $P_{go}(0)$	23.6 – 34.0	–	mmHg	The value of P_{go} in healthy individuals: 27.3 ± 2.6 mmHg [Škrtić], 26.0 ± 2.4 mmHg [Chagnac], 30.0 ± 4.0 mmHg [Guasch].
48	Renal venous pressure, P_v	6.0	Hypertension 2.0 – 6.0	mmHg	The normal value P_v = 6 mmHg is taken from [Digne-Malcolm]. In patients with essential hypertension, P_v is decreased by more than 50% [Willassen]. Therefore, as the lower bound of this parameter we used 2 mmHg.
49	Normal plasma renin concentration, PRC_{nom}	3.42 – 69.4	–	$pg \cdot ml^{-1}$	The normal range [Perschel].
50	Nominal resistance of interlobar, arcuate and interlobular arteries, $R_{preglom\_0}$	7.0 – 20.0	Hypertension 7.0 – 28.0	$mmHg \cdot min \cdot l^{-1}$	Hallow et al. give the normal range for $R_{preglom\_0}$ of 10.0 – 20.0 $mmHg \cdot min \cdot l^{-1}$ [Hallow, 2017a], and consider 40% increase in the nominal value (from 14.0 to 19.6 $mmHg \cdot min \cdot l^{-1}$) in salt-resistant hypertensive patients [Hallow, 2017b]. The parameter estimation of our model resulted in the normal nominal value of 10.0 $mmHg \cdot min \cdot l^{-1}$, and, therefore, we assumed the normal range of 7.0 – 20.0 $mmHg \cdot min \cdot l^{-1}$. As the range in hypertensive patients, we considered 7.0 – 28.0 $mmHg \cdot min \cdot l^{-1}$ with 40% increase in the upper bound.
51	Renal venous resistance, R_v	11.3 – 20.1	–	$mmHg \cdot min \cdot l^{-1}$	The estimate of R_v in healthy persons is given in Appendix B.
52	Body oxygen demand, RO_{20}	2.52 – 5.88	–	$ml \cdot s^{-1}$	The average value of RO_{20} is approximately 4.2 $ml \cdot s^{-1}$ [Treacher]. We considered the deviation of this value by 40%.

53	Arterial oxygen saturation, SpO_2	0.95 – 0.99	Hypertension, CHF, PH 0.92 – 0.99	–	SpO_2 is normally 95 – 99% [Goldberg]. As the lower bound in patients with cardiovascular deseases, we took the practical lower threshold to rule out hypoxaemia [Beasley].
54	Total protein, *TP*	60.0 – 86.0	–	$g \cdot l^{-1}$	The normal ranges: 60.0 – 80.0 $g \cdot l^{-1}$ [Busher], 66.0 – 86.0 $g \cdot l^{-1}$ [Gardner].
55	Plasma urea concentration, *urea*	1.8 – 7.1	–	$mmol \cdot l^{-1}$	The normal range [Hosten].
56	Initial value of total body water, $TBW(0)$	$f(0.9 \cdot N_{BV})$ – $f(1.1 \cdot N_{BV})$	CHF, class III/IV: $f(0.9 \cdot N_{BV})$ – $f(1.4 \cdot N_{BV})$	l	As the range of total blood volume (V) in normal humans, we considered $N_{BV} \pm 10\%$ with the value N_{BV} defined by the Nadler equation [Nadler]. In patients with NYHA classes III-IV, we used an increased upper bound of V (up to 40%) [Miller]. We calculated the initial *TBW* by the formula $f(x) = (x - 650)/111.5$ [Moore, 1967].
57	Initial value of venous oxygen content, $VO_2(0)$	0.0855 – 0.1848	–	–	VO_2 is normally 9.5 – 16.8 mL/dL [Hattori]. We considered additional deviation from this range by 10%.
58	Equilibrium ratio of *PRA* to *PRC*, X_{PRC_PRA}	0.61 – 1.42	–	$fmol \cdot min^{-1} \cdot pg^{-1}$	The normal value of X_{PRC_PRA} = 1.016666 $fmol \cdot min^{-1} \cdot pg^{-1}$ is given in [Hallow, 2014]. We assumed a deviation from this value of ± 40% as the fluctuation range.
59	Basic conductivity of systemic microvessels, Y_{ALVL0}	1.363	Hypertension CHF PH 0.5 – 2.0	$ml \cdot s^{-1} \cdot mmHg^{-1}$	Hypertension is associated with the *SVR* elevation [Stefanadis], [Prys-Roberts], which can be caused by the decline in Y_{ALVL0}. If we take Y_{ALVL0} = 0.5 $ml \cdot s^{-1} \cdot mmHg^{-1}$ instead of the normal value 1.363 $ml \cdot s^{-1} \cdot mmHg^{-1}$ in the model, the *SVR* level increases to the value 1.1 $s \cdot mmHg \cdot ml^{-1}$ closed to the upper bound of the norm [Klingensmith]. In patients with PH and NYHA class III the value of *SVR* can be also increased [Opitz]. Thus, as the reasonable range of values in patients with cardiovascular diseases we considered the interval 0.5 – 2.0 $ml \cdot s^{-1} \cdot mmHg^{-1}$.
60	Basic conductivity of pulmonary microvessels, Y_{ARVR0}	9.0 – 21.0	LVH, PH 1.0 – 21.0	$ml \cdot s^{-1} \cdot mmHg^{-1}$	According to our estimates, the normal value of Y_{ARVR0} is 15.0 $ml \cdot s^{-1} \cdot mmHg^{-1}$. We accept a deviation by ± 40%. Without changing the other model parameters, such deviation gives the normal interval 0.074 – 0.138 $s \cdot mmHg \cdot ml^{-1}$ for *PVR* (see acceptable clinical evaluations of *PVR* in Table C2). LVH and PH associated with the increased *PVR* [Stefanadis], [Gan]. Thus, for these diseases, we considered the Y_{ARVR0} range of 1.0 – 21.0 $ml \cdot s^{-1} \cdot mmHg^{-1}$ resulting in the *PVR* interval of 0.074 – 1.058 $s \cdot mmHg \cdot ml^{-1}$.
61	Basic conductivity of the pulmonary valve and arteries, Y_{HRAR}	57.0	PH 5.0 – 70.0	$ml \cdot s^{-1} \cdot mmHg^{-1}$	We estimate Y_{HRAR} in patients with PH and increased value of $PVR = 1/Y_{HRAR} + 1/Y_{ARVR} + 1/Y_{VRHL}$. As the range of fluctuations, we used the interval of 5.0 – 70.0 $ml \cdot s^{-1} \cdot mmHg^{-1}$.
62	Basic conductivity of the mitral valve and pulmonary veins, Y_{VRHL0}	57.0	LVDD PH 5.0 – 70.0	$ml \cdot s^{-1} \cdot mmHg^{-1}$	The value of Y_{VRHL0} directly affects transmitral flow F_{VRHL}. Therefore, we varied this parameter in the case of patients with the LV diastolic dysfunction (LVDD), when F_{VRHL} is altered [Caudron], [Kawaji]. We also estimate Y_{VRHL0} in patients with PH and increased value of $PVR = 1/Y_{HRAR} + 1/Y_{ARVR} + 1/Y_{VRHL}$. As the range of fluctuations, we used the interval of 5.0 – 70.0 $ml \cdot s^{-1} \cdot mmHg^{-1}$.

63	Nominal value of macula densa sodium flow rate, $\Phi_{md\_sod\_0}$	1.0 – 4.0	–	$mEq \cdot min^{-1}$	According to our estimates for a healthy person, the value of $\Phi_{md\_sod\_0}$ is 3.32 $mEq \cdot min^{-1}$. As the fluctuation range, we considered the interval 1.0 – 4.0 $mEq \cdot min^{-1}$.
64	Sodium intake, Φ_{sodin}	0.0280 – 0.2088	–	$mEq \cdot min^{-1}$	The normal range 0.035 – 0.174 $mEq \cdot min^{-1}$ is taken from the study [Luft]. We assumed a possible deviation from this range by 20%.
65	Normal value of water intake, Φ_{win_norm}	0.00096 – 0.00312	–	$l \cdot min^{-1}$	The average statistical range estimate is 0.0019 ± 0.0007 $l \cdot min^{-1}$ [Malisova]. We assumed a possible deviation from this range by 20%.

Table C2. List of constraints imposed on the clinically measurable variables in patients with cardiovascular diseases and used in the model.

№	Variables, notation in the model	Constraints		Units	Justification of constraints
		Normal	Abnormal		
01	Arterial oxygen content, AO_2	0.145 – 0.244	–	–	The normal range [Hattori].
02	Plasma angiotensin (1-7), *ANG17*	14.1 – 31.7	Hypertension 12.7 – 34.9	$fmol \cdot ml^{-1}$	The normal estimate is 22.9 ± 8.8 $fmol \cdot ml^{-1}$ [Ferrario].<br>We extended the bounds by 10% in the case of hypertensive patients.
03	Plasma angiotensin I, *ANGI*	2.8 – 13.6	Hypertension 2.5 – 15.0	$fmol \cdot ml^{-1}$	The normal estimate is 8.2 ± 5.4 $fmol \cdot ml^{-1}$ [Nussberger].<br>We extended the bounds by 10% in the case of hypertensive patients.
04	Plasma angiotensin II, *ANGII*	3.3 – 7.1	CHF 3.3 – 14.0	$fmol \cdot ml^{-1}$	The normal estimate is 5.2 ± 1.9 $fmol \cdot ml^{-1}$ [Nussberger].<br>Patients with CHF: 4.2 – 14.0 $pmol \cdot l^{-1}$ [Smilde].
05	Plasma vasopressin, C_{adh}	1.0 – 13.3	CHF, class III/IV 1.0 – 23.3	$pg \cdot ml^{-1}$	The normal range is given in [Yarmohammadi]. The estimations in patients with severe congestive heart failure and primary cardiomyopathy (classes III to IV, NYHA): 3.9 ± 1.0 and 14.5 ± 8.8 $pg \cdot ml^{-1}$ [Riegger].
06	Plasma aldosterone, C_{al}	70 – 300	–	$pg \cdot ml^{-1}$	The normal range [Fischbach].
07	Plasma atriopeptin, C_{anp}	7.4 – 152.0	CHF, class I 37.1 – 152.0<br>CHF, class II 56.0 – 152.0<br>CHF, class III 161.0 – 253.0<br>CHF, class IV 257.0 – 838.0<br>CRF 133.0 – 305.0	$ng \cdot l^{-1}$	Normal ranges: 27 – 152 $ng \cdot l^{-1}$ [Nozuki], 7.4 – 15.7 $ng \cdot l^{-1}$ [Cannone], 20 ± 7 $ng \cdot l^{-1}$ [Wei], 35.4 ± 4.6 $ng \cdot l^{-1}$ [Kato]. There is no significant difference in plasma atriopeptin levels between man and woman [Nozuki]. Patients with congestive heart failure (CHF), NYHA I: 42.9 ± 5.8 $ng \cdot l^{-1}$ [Kato], NYHA I-II: 68 ± 12 $ng \cdot l^{-1}$ [Wei], NYHA III: 217 ± 36 $ng \cdot l^{-1}$, NYHA III-IV: 209 ± 48 $ng \cdot l^{-1}$ [Wei], NYHA IV: 571 ± 267 $ng \cdot l^{-1}$ [Nozuki]. Patients with chronic renal failure (CRF): 219 ± 86 $ng \cdot l^{-1}$ [Nozuki]. The following diseases do not result in a significant difference of atriopeptin levels from the norm [Nozuki]: atrial fibrillation, pacemaker implantation, lung disease, chronic glomerulonephritis, nephrotic syndrome, essential hypertension [Cannone], liver disease and cerebrovascular disease.
08	Plasma sodium, C_{sod}	137 – 147	–	$mEq \cdot l^{-1}$	The normal range [Payne].

09	Cardiac output, *CO*	2.51 – 9.00	–	$l \cdot min^{-1}$	The normal range (age 18+) was taken from [Cattermole]. Patients with essential hypertension: 3.90 ± 1.08 $l \cdot min^{-1}$ [Cléroux], 7.17 ± 1.58 $l \cdot min^{-1}$ [Safar, 1976].
10	Ejection fraction, *EF*	50 – 80	CHF<br>30 – 80<br>PH<br>20 – 80	%	The normal value is $EF \geq 50$ [Pfisterer]. In normal active subjects, *EF* is reached 70 – 80% [Saghiv]. In patients with CHF, *EF* can be reduced to 30% [Dushina]. In patients with PH, EF can achieve 20% [Wright, 2017].
11	Systemic microvessels blood flow, F_{ALVL}	> 0.0	–	$ml \cdot s^{-1}$	The constraint characterizes the movement of blood from the aorta to the right atrium strictly in one direction.
12	Pulmonary microvessels blood flow, F_{ARVR}	> 0.0	–	$ml \cdot s^{-1}$	The constraint characterizes the movement of blood from the pulmonary artery to the left atrium strictly in one direction.
13	Transaortic flow peak rate, F_{HLAL_p}	347.0 – 677.0	–	$ml \cdot s^{-1}$	The normal estimate is 512 ± 165 $ml \cdot s^{-1}$ [Kyhl].
14	Transpulmonary flow peak rate, F_{HRAR_p}	264.5 – 793.0	–	$ml \cdot s^{-1}$	The range of values was chosen in accordance with estimates in healthy individuals: 611 ± 182 $ml \cdot s^{-1}$ [Kyhl], 463 ± 71 $ml \cdot s^{-1}$ [Widya], 332.9 ± 68.4 $ml \cdot s^{-1}$ [Macedo].
15	Active peak RV filling rate, F_{VLHR_ap}	M: 23 – 947<br>W: 54 – 680	–	$ml \cdot s^{-1}$	As bounds, we considered the minimum and maximum values received for mans and womans for all ages (20–79 years) in the population of 120 individuals [Maceira, 2006b].
16	Early peak RV filling rate, F_{VLHR_ep}	M: 8 – 814<br>W: -17 – 701	–	$ml \cdot s^{-1}$	
17	F_{VLHR_ep}/F_{VLHR_ap}	M: -0.5 – 2.5<br>W: -0.4 – 2.5	–	–	
18	Active peak LV filling rate, F_{VRHL_ap}	M: 99 – 647<br>W: 58 – 508	–	$ml \cdot s^{-1}$	As bounds, we considered the minimum and maximum values received for mans and womans for all ages (20–79 years) in the population of 120 individuals [Maceira, 2006a].
19	Early peak LV filling rate, F_{VRHL_ep}	M: 21 – 1034<br>W: -13 – 967	–	$ml \cdot s^{-1}$	
20	F_{VRHL_ep}/F_{VRHL_ap}	M: 0.3 – 5.9<br>W: 0.3 – 6.6	–	–	
21	Systemic arterial elastance, G_{AL}	0.33 – 1.00	Hypertension<br>0.33 – 1.67<br>CHF, LVH<br>0.33 – 2.38<br>PH: 0.71 – 1.43	$mmHg \cdot ml^{-1}$	See parameter G_{AL0} in Table C1 for details.
22	Pulmonary arterial elastance, G_{AR}	0.08 – 0.26	CHF<br>0.08 – 0.50<br>PH: 0.26 – 2.50	$mmHg \cdot ml^{-1}$	See parameter G_{AR0} in Table C1 for details.

23	Glomerular filtration rate, *GFR*	0.060 – 0.135	CHF<br>0.046 – 0.135<br>CRF<br>0.030 – 0.135	$l \cdot min^{-1}$	*GFR* is normally (in the absence of evidence of kidney damage) 60 – 135 ml/min/1.73 m^2 [Levin], [Cachat]. Patients with CHF: 74 ± 28 ml/min/1.73 m^2 [Smilde]. As a lower bound in patients with chronic renal failure (CRF), we used 30 ml/min/1.73 m^2 [Levin].
24	Heart rate, $Heart_{Rate}$	60 – 100	–	$beats \cdot min^{-1}$	The normal range [Ostchega].
25	Total exchangeable sodium, M_{sod}	2040 – 3950	–	mEq	The normal range [Farber].
26	Normal fractional sodium reabsorption in the distal tubule and subsequent parts of the nephron, $n_{\varepsilon\_dt} + n_{\eta\_cd} - n_{\eta\_cd} \cdot n_{\varepsilon\_dt}$	0.78 – 0.98	–	–	The normal estimates: 0.946 ± 0.032 (mans), 0.950 ± 0.031 (womans) [Seidlerová], 0.96 ± 0.02 (young), 0.92 ± 0.06 (elderly) [Fliser]. Randomly recruited 95 nuclear families of black South African ancestry: 0.850 – 0.948, and 103 nuclear families of white Belgian ancestry: 0.930 – 0.966 [Bochud]. Randomly recruited 317 untreated subjects from a white population: 0.931 ± 0.044 in low and 0.859 ± 0.074 in high sodium excretion [Jin].
27	Plasma osmolality, *osmolality*	275 – 295	–	$mOsmol \cdot kg^{-1}$	The normal range [Fogarty].
28	Diastolic pulmonary arterial pressure, P_{AR_D}	4.0 – 12.0	CHF, class I<br>4.0 – 20.2<br>CHF, class II-IV<br>7.0 – 20.2<br>PH<br>12.3 – 50.0	mmHg	Normal: 4.0 – 12.0 mmHg [Marini], [Pagani]. Evaluation in 882 healthy volunteers: 8.8 ± 3.0 mmHg [Kovacs]. Patients with isolated LVDD and NYHA class II/III: 11.0 ± 4.0 mmHg [Kasner, 2012]. Patients with NYHA class II: 14.4 ± 5.8 mmHg; patients with PH and NYHA class II/III: 22.4 ± 10.1 mmHg [Murch]. Patients with PH: 37.0 ± 13.0 mmHg [Gan].
29	Systolic pulmonary arterial pressure, P_{AR_S}	15.0 – 30.0	CHF<br>14.0 – 44.2<br>PH<br>37.4 – 107.0	mmHg	Normal: 15.0 – 30.0 mmHg [Marini], [Pagani]. Evaluation in 882 healthy humans: 20.8 ± 4.4 mmHg [Kovacs]. Patients with isolated LV diastolic dysfunction and NYHA class II/III: 22.0 ± 8.0 mmHg [Kasner, 2012]. Patients with NYHA class II: 35.4 ± 8.8 mmHg; patients with PH and NYHA class II/III: 60.2 ± 22.8 mmHg [Murch]. Patients with PH: 84.0 ± 23.0 mmHg [Gan].
30	Diastolic blood pressure, P_D	< 90	Hypertension<br>> 90	mmHg	Definitions of hypertension based on the 2013 European Society of Hypertension and European Society of Cardiology guidelines [Oparil].
31	Glomerular hydrostatic pressure, P_{gh}	48.0 – 63.0	–	mmHg	The normal estimates: 59.6 ± 2.7 mmHg [Škrtić], 54.3 ± 4.5 [Tsuda], 48.0 – 63.0 [Guberina].
32	Glomerular capillary oncotic pressure, P_{go}	23.6 – 34.0	–	mmHg	The range is given in Table C1 for the initial value of the parameter.

33	LV diastolic pressure, P_{HL_D}	3.0 – 12.0	Hypertension<br>1.0 – 18.0<br>CHF, class I/II<br>1.0 – 18.9<br>CHF, class III/IV<br>4.3 – 18.9<br>LVH<br>1.0 – 20.0	mmHg	Normal: 3 – 12 mmHg [Pagani]. The minimal LV pressure in patients without heart failure symptoms and with normal ejection fraction: 0.9 – 3.2 mmHg; in patients with heart failure (NYHA class II/III) and normal ejection fraction: 4.3 – 9.4 mmHg [Kasner, 2007]. LV end-diastolic pressure in patients with heart failure (NYHA class II/III) and normal ejection fraction: 12.0 – 18.9 mmHg [Kasner, 2007]. The same pressure in 313 patients without coronary artery disease, but with the possible hypertension (37.1% of patients) or diabetes mellitus (8.3%): 9.58 ± 5.78 mmHg [Du]. Evaluation in untreated hypertensive patients with LVH – 15 ± 5 mmHg, and without it – 15 ± 3 mmHg [Antony].
34	LV systolic pressure, P_{HL_S}	100 – 140	CHF<br>100 – 154<br>Hypertension<br>100 – 186<br>LVH<br>100 – 200	mmHg	Normal: 100 – 140 mmHg [Pagani]. Peak value in patients with diastolic heart dysfunction (concentric LVH) due to essential hypertension: 162.46 ± 8.21 mmHg [Stefanadis]. Evaluation in untreated hypertensive patients with LVH – 184 ± 16 mmHg, and without it – 172 ± 14 mmHg [Antony]. LV end-systolic pressure in patients with heart failure (NYHA class II/III) and normal ejection fraction: 116 – 154 mmHg [Kasner, 2007].
35	RV diastolic pressure, P_{HR_D}	2.0 – 8.0	Hypertension<br>0.0 – 10.0<br>CHF<br>0.0 – 12.0<br>PH<br>8.0 – 22.0	mmHg	Normal: 2 – 8 mmHg [Pagani]. Evaluation of RV end-diastolic pressure in patients with uncomplicated, asymptomatic essential hypertension: 1.0 – 10.0 mmHg [Ferlinz]. Patients with isolated LVDD and NYHA class II/III: 6.0 ± 6.0 mmHg [Kasner, 2012]. Patients with PH: 15.0 ± 7.0 mmHg [Gan].
36	RV systolic pressure, P_{HR_S}	15.0 – 30.0	CHF, class I/II<br>15.0 – 40.0<br>CHF, class III/IV<br>20.0 – 40.0<br>PH<br>62.0 – 110.0	mmHg	Normal: 15.0 – 30.0 mmHg [Pagani]. Patients with isolated LVDD and NYHA class II/III: 30.0 ± 10.0 mmHg [Kasner, 2012]. Patients with PH: 86.0 ± 24.0 mmHg [Gan].
37	Systolic blood pressure, P_S	< 140	Hypertension<br>> 140	mmHg	Definitions of hypertension based on the 2013 European Society of Hypertension and European Society of Cardiology guidelines [Oparil].
38	Systemic venous pressure (central venous pressure, approximation of right atrial pressure)., P_{VL}	2.0 – 8.0	Hypertension<br>1.0 – 10.0<br>CHF<br>1.0 – 12.0<br>PH<br>5.0 – 20.0	mmHg	Normal: 2 – 8 mmHg [Klingensmith]. Mean value in patients with uncomplicated, asymptomatic essential hypertension: 1.0 – 10.0 mmHg [Ferlinz]. Patients with PH: 10.0 ± 5.0 mmHg [Gan]. Patients with acute decompensated heart failure: 6.0 – 18.0 mmHg [Sperry]. Patients with heart failure: 6.4 ± 5.7 mmHg in low *PVR* (<200 dyn·s·cm^{-5}), 8.7 ± 3.7 mmHg in high *PVR* [Melenovsky]. Patients with primary PH and NYHA class III: 13.0 ± 7.0 mmHg [Opitz]. Patients with normal LV ejection fraction and exertional dyspnea: 3.0 – 16.0 [Nagueh].

39	Pulmonary venous pressure, P_{VR}	3.0 – 20.0	–	mmHg	The normal range is given in Table A4.
40	Plasma renin activity, *PRA*	15.0 – 31.7	Hypertension 12.0 – 38.0	fmol·ml^{-1}·min^{-1}	The normal range is 0.9 – 1.9 pmol/ml/h [Valabhji]. In the case of hypertensive patients, we extended the bounds by 20%.
41	Plasma renin concentration, *PRC*	3.42 – 69.4	–	pg·ml^{-1}	The normal range [Perschel].
42	Resistance of afferent vessels (arteries and arterioles), R_{a_dyne}	753 – 6863	Hypertension 3000 – 25000	dyn·s·cm^{-5}	The normal estimates: 753 – 2532 dyn·s·cm^{-5} (Appendix B), 4629.3 ± 2233.2 dyn·s·cm^{-5} [Tsuda]. In patients with essential hypertension, R_{a_dyne} is, on average, five times higher than in normotensive subjects [Gómez].
43	Resistance of efferent arterioles, R_{e_dyne}	1350 – 3400	–	dyn·s·cm^{-5}	Evaluation in healthy individuals (1669 – 2843 dyn·s·cm^{-5}) is given in Appendix B. In addition, we considered a deviation from this range by 20%.
44	Resistance of interlobar, arcuate and interlobular arteries, $R_{preglom}$	7.0 – 20.0	Hypertension 7.0 – 28.0	mmHg·min·l^{-1}	The range is given in Table C1 for the parameter $R_{preglom\_0}$.
45	Renal blood flow, *RBF*	0.623 – 1.730	CHF 0.304 – 1.730	l·min^{-1}	The normal range is provided in the work [Bax]. Patients with CHF: 465 ± 161 ml/min/1.73 m^2 [Smilde].
46	Renal vascular resistance, *RVR*	55.0 – 84.0	Hypertension 55.0 – 190.0 CHF 55.0 – 230.0	mmHg·min·l^{-1}	Evaluation in healthy individuals is derived in Appendix B. In patients with essential hypertension, the *RVR* value can rise to 190 mmHg·min·l^{-1} [Bauer]. Patients with CHF: 150 – 230 mmHg· min·l^{-1} [Smilde].
47	Total blood volume, *V*	$0.9 \cdot N_{BV}$ – $1.1 \cdot N_{BV}$	CHF, class III/IV: $0.9 \cdot N_{BV}$ – $1.4 \cdot N_{BV}$	ml	As the normal range of *V*, we considered N_{BV} ± 10%, where N_{BV} is the estimation calculated by the Nadler equation [Nadler]. In patients with NYHA classes III-IV, the value of *V* can exceed the expected normal volume by an average 40% [Miller].
48	LV end-diastolic volume, V_{HL_KD}	M: 67 – 155 W: 56 – 104	CHF, class I/II 56 – 353 CHF, class III/IV 93 – 621	ml	The normal range is given in [Lang]. Patients with PH and NYHA class I-III: 86 ± 30 ml [Finsberg]. Patients with congestive heart failure due to nonischemic dilated cardiomyopathy (NYHA II/III): 312 ± 41 ml [Tkacova]. Patients with advanced heart failure (NYHA class III/IV) caused by idiopathic dilated cardiomyopathy (IDC) or ischemic cardiomyopathy (IsC) and a prolonged QRS complex: 93 – 423 ml [Stellbrink]. Patients with congestive heart failure (NYHA I-IV, mainly III) and IDC: 521 ± 100 ml, or IsC: 309 ± 34 ml [Mehta].
49	LV end-systolic volume, V_{HL_KS}	M: 22 – 58 W: 19 – 49	CHF, class I-II 16 – 281 CHF, class III/IV 70 – 526	ml	The normal range [Lang]. Patients with PH and NYHA class I-III: 37 ± 21 ml [Finsberg]. Patients with congestive heart failure due to nonischemic dilated cardiomyopathy (NYHA II/III): 246 ± 35 ml [Tkacova]. Patients with advanced heart failure (NYHA class III/IV) caused by IDC or IsC and a prolonged QRS complex: 70 – 396 ml [Stellbrink]. Patients with congestive heart failure (NYHA I-IV, mainly III) and IDC: 430 ± 96 ml, or IsC: 236 ± 29 ml [Mehta].

50	RV end-diastolic volume, V_{HR_KD}	M: 124 – 256<br>W: 78 – 218	CHF, class I-II<br>62 – 256<br>CHF, class III/IV<br>93 – 604	ml	The normal range is given in [Hudsmith]. Patients with PH and NYHA class I-III: 134 ± 72 ml [Finsberg]. Patients with congestive heart failure (NYHA I-IV, mainly III) and IDC: 527 ± 77 ml, or IsC: 490 ± 54 ml [Mehta].
51	RV end-systolic volume, V_{HR_KS}	M: 38 – 118<br>W: 20 – 92	CHF, class I-II<br>23 – 159<br>CHF, class III/IV<br>70 – 478	ml	The normal range is given in [Hudsmith]. Patients with PH and NYHA class I-III: 91 ± 68 ml [Finsberg]. Patients with congestive heart failure (NYHA I-IV, mainly III) and IDC: 400 ± 78 ml, or IsC: 370 ± 44 ml [Mehta].
52	Venous oxygen content, VO_2	0.0855 – 0.1848	–	–	The normal range can be found in [Hattori]. We considered additional deviation from this range by 10%.
53	Systemic vascular resistance, $1/Y_{HLAL}+1/Y_{ALVL}+1/Y_{VLHR}$	0.5271 – 1.2048	Hypertension<br>0.5271 – 1.9608<br>CHF<br>0.5271 – 2.8366<br>LVH<br>0.5271 – 1.5493<br>PH<br>0.6416 – 2.3991	s·mmHg·ml^{-1}	Normal: 0.5271 – 1.2048 s·mmHg·ml^{-1} [Klingensmith] (using the conversion factor 1/1328 from dyn·s·cm^{-5} to s·mmHg·ml^{-1} [Gómez]). Hypertensive elderly patients: 1.6762 ± 0.2846 s·mmHg·ml^{-1} [Prys-Roberts]. Patients with diastolic heart dysfunction (concentric LVH) due to essential hypertension: 1.3946 ± 0.1547 s·mmHg·ml^{-1} [Stefanadis]. Patients with congestive heart failure: 0.7108 – 1.6634 s·mmHg·ml^{-1} [Laskey, 1990], 1.710 – 2.352 s·mmHg·ml^{-1} [Sobol]. Patients with primary PH and NYHA class III: 1.7515 ± 0.6476 s·mmHg·ml^{-1} [Opitz]. Hypertensive patients with congestive heart failure: 1.434 – 3.774 s·mmHg·ml^{-1} [Sobol]. Patients with heart failure: 1.2116 ± 0.3780 s·mmHg·ml^{-1} in low *PVR* (<200 dyn·s·cm^{-5}), 1.3178 ± 0.4164 s·mmHg·ml^{-1} in high *PVR* [Melenovsky]. Patients with PH with below threshold *PVR*: 0.6416 – 0.9337 s·mmHg·ml^{-1} [Ratwatte]. Healthy subjects and patients with miscellaneous cardiac diseases: 0.7410 – 2.8366 s·mmHg·ml^{-1} [Chemla, 2017].
54	Pulmonary vascular resistance, $1/Y_{HRAR}+1/Y_{ARVR}+1/Y_{VRHL}$	0.0151 – 0.1353	LVH<br>0.0151 – 0.3080<br>PH<br>0.1320 – 1.2071	s·mmHg·ml^{-1}	Normal: 0.0151 – 0.0979 s·mmHg·ml^{-1} [Klingensmith] (using the conversion factor 1/1328 from dyn·s·cm^{-5} to s·mmHg·ml^{-1} [Gómez]). Evaluation in 10 healthy subjects [Stefanadis]: 0.0989 ± 0.0364 s·mmHg·ml^{-1}. Patients with diastolic heart dysfunction (concentric LVH) due to essential hypertension: 0.2497 ± 0.0583 s·mmHg·ml^{-1} [Stefanadis]. Patients with heart failure: 0.0828 – 0.1288 s·mmHg·ml^{-1} in low *PVR* (<200 dyn·s·cm^{-5}), 0.2229 – 0.4571 s·mmHg·ml^{-1} in high *PVR* [Melenovsky]. Patients with PH: 0.7523 ± 0.4548 s·mmHg·ml^{-1} [Gan]. Patients with PH and left heart disease: 0.1320 – 0.6300 s·mmHg·ml^{-1} [Wright, 2017] (using the conversion 1WU = min·mmHg·l^{-1} [Kwan]).
55	Fractional proximal sodium reabsorption, $\eta_{pt\_sodreab}$	0.67 – 0.97	–	–	The normal range is given in Table C1 for the parameter n_{η_pt}. Estimations in elderly hypertensive: 0.86 ± 0.07, and elderly with heart failure: 0.87 ± 0.0 [Fliser].

56	Fractional sodium reabsorption in the distal tubule and subsequent parts of the nephron, $\eta_{dt\_sodreab} + \eta_{cd\_sodreab} - \eta_{dt\_sodreab} \cdot \eta_{cd\_sodreab}$	0.78 – 0.98	–	–	The normal range is given for the expression $n_{\varepsilon\_dt} + n_{\eta\_cd} - n_{\eta\_cd} \cdot n_{\varepsilon\_dt}$ above. Estimations in elderly hypertensive: 0.93 ± 0.05, and elderly with heart failure: 0.89 ± 0.07 [Fliser].
57	Water intake, Φ_{win}	0.00096 – 0.00312	–	$l \cdot min^{-1}$	The average statistical range estimate is 0.0019 ± 0.0007 $l \cdot min^{-1}$ [Malisova]. We assumed a possible deviation from this range by 20%.

REFERENCES

Admiraal PJ, Danser AH, Jong MS, Pieterman H, Derkx FH, Schalekamp MA (1993) Regional angiotensin II production in essential hypertension and renal artery stenosis. Hypertension 21:173–184.

Andrushkevich VV (2006) Blood biochemical parameters, their reference values, reasons for level change in serum. Novosibirsk. 28 p. (In Russ.)

Antony I, Nitenberg A, Foult JM, Aptecar E (1993) Coronary vasodilator reserve in untreated and treated hypertensive patients with and without left ventricular hypertrophy. Journal of the American College of Cardiology 22(2):514–520.

Amberg GC, Bonev AD, Rossow CF, Nelson MT, Santana LF (2003) Modulation of the molecular composition of large conductance, Ca(2+) activated K(+) channels in vascular smooth muscle during hypertension. The Journal of Clinical Investigation 112(5):717–724.

Ardaillou R, Chansel D, Chatziantoniou C, Dussaule JC (1999) Mesangial AT1 receptors: expression, signaling, and regulation. Journal of the American Society of Nephrology Suppl 11:S40–S46.

Bastos MB, Burkhoff D, Maly J, Daemen J, den Uil CA, Ameloot K, Lenzen M, Mahfoud F, Zijlstra F, Schreuder JJ, Van Mieghem NM (2020) Invasive left ventricle pressure-volume analysis: overview and practical clinical implications. European Heart Journal 41:1286–1297.

Bauer JH, Brooks CS, Burch RN (1982) Renal function and hemodynamic studies in low- and normal-renin essential hypertension. Archives of Internal Medicine 142:1317–1323.

Bax L, Bakker CJG, Klein WM, Blanken N, Beutler JJ, Mali WPTRM (2005) Renal blood flow measurements with use of phase-contrast magnetic resonance imaging: normal values and reproducibility. Journal of Vascular and Interventional Radiology 16:807–814.

Beasley R, Chien J, Douglas J, Eastlake L, Farah C, King G, Moore R, Pilcher J, Richards M, Smith S, Walters H (2015) Thoracic Society of Australia and New Zealand oxygen guidelines for acute oxygen use in adults: 'Swimming between the flags'. Respirology 20:1182–1191.

Bertram JF, Douglas-Denton RN, Diouf B, Hughson MD, Hoy WE (2011) Human nephron number: implications for health and disease. Pediatric Nephrology 26:1529–1533.

Bhagat CI, Garcia-Webb P, Fletcher E, Beilby JP (1984) Calculated vs measured plasma osmolalities revisited. Clinical Chemistry 30(10):1703–1705.

Bhella PS, Hastings JL, Fujimoto N, Shibata S, Carrick-Ranson G, Palmer MD, Boyd KN, Adams-Huet B, Levine BD (2014) Impact of lifelong exercise "dose" on left ventricular compliance and distensibility. Journal of the American College of Cardiology 64(12):1257–1266.

Billett HH (1990) Hemoglobin and hematocrit. In: Clinical methods: The history, physical, and laboratory examinations. 3rd edition. Editors: Walker HK, Hall WD, Hurst JW. Boston: Butterworths.

Blais C, Pibarot P, Dumesnil JG, Garcia D, Chen D, Durand LG (2001) Comparison of valve resistance with effective orifice area regarding flow dependence. American Journal of Cardiology 88:45–52.

Blantz RC, Konnen KS, Tucker BJ (1976) Angiotensin II effects upon the glomerular microcirculation and ultrafiltration coefficient of the rat. The Journal of Clinical Investigation 57:419–434.

Bochud M, Staessen JA, Maillard M, Mazeko MJ, Kuznetsova T, Woodiwiss A, Richart T, Norton G, Thijs L, Elston R, Burnier M (2009) Ethnic differences in proximal and distal tubular sodium reabsorption are heritable in black and white populations. Journal of Hypertension 27:606–612.

Boer WH, Koomans HA, Dorhout Mees EJ (1987) Lithium clearance during the paradoxical natriuresis of hypotonic expansion in man. Kidney International 32:376–381.

Bojestig M, Nystrom FH, Arnqvist HJ, Ludvigsson J, Karlberg BE (2000) The renin-angiotensin-aldosterone system is suppressed in adults with Type 1 diabetes. Journal of the Renin-Angiotensin-Aldosterone System 1:353–356.

Bollag WB (2014) Regulation of aldosterone synthesis and secretion. Comprehensive Physiology 4:1017–1055.

Boogers MJ, van Werkhoven JM, Schuijf JD, Delgado V, El-Naggar HM, Boersma E, Nucifora G, van der Geest RJ, Paelinck BP, Kroft LJ, Reiber JH, de Roos A, Bax JJ, Lamb HJ (2011) Feasibility of diastolic function assessment with cardiac CT: feasibility study in comparison with tissue Doppler imaging. JACC: Cardiovascular Imaging 4(3):246–256.

Brasch H, Sieroslawski L, Dominiak P (1993) Angiotensin II increases norepinephrine release from atria by acting on angiotensin subtype 1 receptors. Hypertension 22:699–704.

Brooks DE, Goodwin JW, Seaman GV (1970) Interactions among erythrocytes under shear. Journal of Applied Physiology 28(2):172–177.

Brown WJJr, Brown FK, Krishan I (1970) Exchangeable sodium and blood volume in normotensive and hypertensive humans on high and low sodium intake. Circulation 43:508–519.

Brown KA, Ditchey RV (1988) Human right ventricular end-systolic pressure–volume relation defined by maximal elastance. Circulation 78:81–91.

Buglioni A, Cannone V, Cataliotti A, Sangaralingham SJ, Heublein DM, Scott CG, Bailey KR, Rodeheffer RJ, Dessì-Fulgheri P, Sarzani R, Burnett JCJr (2015) Circulating aldosterone and natriuretic peptides in the general community: relationship to cardiorenal and metabolic disease. Hypertension 65(1):45–53.

Burkhoff D, Mirsky I, Suga H (2005) Assessment of systolic and diastolic ventricular properties via pressure-volume analysis: a guide for clinical, translational, and basic researchers. American Journal of Physiology-Heart and Circulatory Physiology 289:H501–H512.

Burwash IG, Pearlman AS, Kraft CD, Miyake-Hull C, Healy NL, Otto CM (1994) Flow dependence of measures of aortic stenosis severity during exercise. Journal of the American College of Cardiology 24(5):1342–1350.

Busher JT (1990) Serum albumin and globulin. In: Clinical methods: The history, physical, and laboratory examinations. 3rd edition. Editors: Walker HK, Hall WD, Hurst JW. Boston: Butterworths.

Cachat F, Combescure C, Cauderay M, Girardin E, Chehade H (2015) A systematic review of glomerular hyperfiltration assessment and definition in the medical literature. Clinical Journal of the American Society of Nephrology 10:382–389.

Cain PA, Ahl R, Hedstrom E, Ugander M, Allansdotter-Johnsson A, Friberg P, Arheden H (2009) Age and gender specific normal values of left ventricular mass, volume and function for gradient echo magnetic resonance imaging: a cross sectional study. BMC Medical Imaging 9:2.

Cannone V, Buglioni A, Sangaralingham SJ, Scott C, Bailey KR, Rodeheffer R, Redfield MM, Sarzani R, Burnett Jr JC (2018) Aldosterone, hypertension, and antihypertensive therapy: insights from a general population. Mayo Clinic Proceedings 93(8):980–990.

Cattermole GN, Leung PY, Ho GY, Lau PW, Chan CP, Chan SS, Smith BE, Graham CA, Rainer TH (2017) The normal ranges of cardiovascular parameters measured using the ultrasonic cardiac output monito. Physiological Reports 5(6):e13195.

Caudron J, Fares J, Bauer F, Dacher JN (2011) Evaluation of left ventricular diastolic function with cardiac MR imaging. RadioGraphics 31:239–261.

Chagnac A, Weinstein T, Korzets A, Ramadan E, Hirsch J, Gafter U (2000) Glomerular hemodynamics in severe obesity. American Journal of Physiology-Renal Physiology 278:F817–F822.

Chaliki HP, Hurrell DG, Nishimura RA, Reinke RA, Appleton CP (2002) Pulmonary venous pressure: relationship to pulmonary artery, pulmonary wedge, and left atrial pressure in normal, lightly sedated dogs. Catheterization and Cardiovascular Interventions 56:432–438.

Chemla D, Hébert JL, Coirault C, Zamani K, Suard I, Colin P, Lecarpentier Y (1998) Total arterial compliance estimated by stroke volume-to-aortic pulse pressure ratio in humans. American Journal of Physiology 274:H500–H505.

Chemla D, Castelain V, Hervé P, Lecarpentier Y, Brimioulle S (2002) Haemodynamic evaluation of pulmonary hypertension. Eur Respir J 20: 1314–1331.

Chemla D, Lau EMT, Hervé P, Millasseau S, Brahimi M, Zhu K, Sattler C, Garcia G, Attal P, Nitenberg A (2017) Influence of critical closing pressure on systemic vascular resistance and total arterial compliance: A clinical invasive study. Arch Cardiovasc Dis 110(12):659–666.

Chen XL, Bayliss DA, Fern RJ, Barrett PQ (1999) A role for T-type Ca^{2+} channels in the synergistic control of aldosterone production by ANG II and K^{+}. American Journal of Physiology 276:F674–F683.

Cherney DZ, Perkins BA, Soleymanlou N, Maione M, Lai V, Lee A, Fagan NM, Woerle HJ, Johansen OE, Broedl UC, von Eynatten M (2014) Renal hemodynamic effect of sodium-glucose cotransporter 2 inhibition in patients with type 1 diabetes mellitus. Circulation 129:587–597.

Chumlea WC, Guo SS, Zeller CM, Reo NV, Siervogel RM (1999) Total body water data for white adults 18 to 64 years of age: the Fels Longitudinal Study. Kidney International 56:244–252.

Cléroux J, Kouamé N, Nadeau A, Coulombe D, Lacourcière Y (1992) Aftereffects of exercise on regional and systemic hemodynamics in hypertension. Hypertension 19(2):183–191.

Consigny PM (1991) Vascular smooth muscle contraction and relaxation: pathways and chemical modulation. Journal of Vascular and Interventional Radiology 2(3):309–317.

Cowley AWJr, Cushman WC, Quillen EWJr, Skelton MM, Langford HG (1981) Vasopressin elevation in essential hypertension and increased responsiveness to sodium intake. Hypertension 3:I93–I100.

Daly WJ, Bondurant S (1962) The effects of oxygen breathing on heart rate, blood pressure, and cardiac index of normal men-resting, with reactive hyperaemia, and after atropine. Journal of Clinical Investigation 41(1):126–132.

Damman K, Navis G, Smilde TD, Voors AA, van der Bij W, van Veldhuisen DJ, Hillege HL (2007) Decreased cardiac output, venous congestion and the association with renal impairment in patients with cardiac dysfunction. European Journal of Heart Failure 9:872–878.

Dedov II, Shestakova MV, Mayorov AYu, editors (2017) Algorithms of specialized medical care for diabetes mellitus patients. 8-th Edition. Moscow: UP Print, 112 p. (In Russ.)

Dehaven JC, Shapiro NZ (1970) Simulation of the renal effects of antidiuretic hormone (ADH) in man. Journal of Theoretical Biology 28:261–286.

Dell'Italia LJ, Walsh RA (1988) Application of a time varying elastance model to right ventricular performance in man. Cardiovascular Research 22:864–874.

Derkx FH, Wenting GJ, Man in't Veld AJ, Verhoeven RP, Schalekamp MA (1978) Control of enzymatically inactive renin in man under various pathological conditions: implications for the

interpretation of renin measurements in peripheral and renal venous plasma. Clinical Science and Molecular Medicine 54:529–538.

Deurenberg P, Tagliabue A, Schouten FJ (1995) Multi-frequency impedance for the prediction of extracellular water and total body water. British Journal of Nutrition 73:349–358.

Digne-Malcolm H, Frise MC, Dorrington KL (2016) How do antihypertensive drugs work? Insights from studies of the renal regulation of arterial blood pressure. Frontiers in Physiology 7:320.

Dijkhuizen P, Buursma A, Fongers TM, Gerding AM, Oeseburg B, Zijlstra WG (1977) The oxygen binding capacity of human haemoglobin. Hüfner's factor redetermined. Pflügers Archiv 369:223–231.

Doenyas-Barak K, de Abreu MHFG, Borges LE, Tavares Filho HA, Yunlin F, Yurong Z, Levin NW, Kaufman AM, Efrati S, Pereg D, Litovchik I, Fuchs S, Minha S (2019) Non-invasive hemodynamic profiling of patients undergoing hemodialysis – a multicenter observational cohort study. BMC Nephrology 20:347.

Donato L, Coli A, Pasqualini R, Duce T (1972) Metabolic clearance rate of radioiodinated angiotensin II in normal men. American Journal of Physiology 223(5):1250–1256.

Dorwart WV, Chalmers L (1975) Comparison of methods for calculating serum osmolality form chemical concentrations, and the prognostic value of such calculations. Clinical Chemistry 21(2):190–194.

Dou Y, Zhu F, Kotanko P (2012) Assessment of extracellular fluid volume and fluid status in hemodialysis patients: current status and technical advances. Seminars in Dialysis 25(4):377–387.

Du LJ, Dong PS, Jia JJ, Fan XM, Yang XM, Wang SX, Yang XS, Li ZJ, Wang HL (2015) Association between left ventricular end-diastolic pressure and coronary artery disease as well as its extent and severity. International Journal of Clinical and Experimental Medicine 8(10):18673–18680.

Dushina AG, Lopina EA, Libis RA (2019) Features of chronic heart failure depending on the left ventricular ejection fraction. Russian Journal of Cardiology 24(Additional issue):5–9.

Edelman IS, Leibman J, O'meara MP, Birkenfeld LW (1958) Interrelations between serum sodium concentration, serum osmolarity and total exchangeable sodium, total exchangeable potassium and total body water. Journal of Clinical Investigation 37(9):1236–1256.

Farber SJ, Soberman RJ (1956) Total body water and total exchangeable sodium in edematous states due to cardiac, renal or hepatic disease. Journal of Clinical Investigation 35(7):779–791.

Faucon AL, Flamant M, Metzger M, Boffa JJ, Haymann JP, Houillier P, Thervet E, Vrtovsnik F, Stengel B, Geri G, Vidal-Petiot E, et al. (2019) Extracellular fluid volume is associated with incident end-stage kidney disease and mortality in patients with chronic kidney disease. Kidney International 96:1020–1029.

Feldschuh J, Enson Y (1977) Prediction of the normal blood volume. Relation of blood volume to body habitus. Circulation 56(4):605–612.

Ferlinz J (1980) Right ventricular performance in essential hypertension. Circulation 61(1):156–162.

Ferrario CM, Martell N, Yunis C, Flack JM, Chappell MC, Brosnihan KB, Dean RH, Fernandez A, Novikov SV, Pinillas C, Luque M (1998) Characterization of angiotensin-(1-7) in the urine of normal and essential hypertensive subjects. American Journal of Hypertension 11:137–146.

Finsberg H, Xi C, Zhao X, Tan JL, Genet M, Sundnes J, Lee LC, Zhong L, Wall ST (2019) Computational quantification of patient-specific changes in ventricular dynamics associated with pulmonary hypertension. Am J Physiol Heart Circ Physiol 317:H1363–H1375.

Fischbach FT (2003) Manual of laboratory and diagnostic test, 7th ed. Philadelphia: Lippincott Williams and Wilkins.

Fliser D, Franek E, Joest M, Block S, Mutschler E, Ritz E (1997) Renal function in the elderly: impact of hypertension and cardiac function. Kidney Int 51:1196–1204.

Fogarty J, Loughrey C (2016) Hyponatraemia in hospitalised adults: a guide for the junior doctor. The Ulster Medical Journal 86(2):84–89.

Fujimoto N, Hastings JL, Bhella PS, Shibata S, Gandhi NK, Carrick-Ranson G, Palmer D, Levine BD (2012) Effect of ageing on left ventricular compliance and distensibility in healthy sedentary humans. The Journal of Physiology 590.8:1871–1880.

Furukawa K, Abumiya T, Sakai K, Hirano M, Osanai T, Shichinohe H, Nakayama N, Kazumata K, Aida T, Houkin K (2016) Measurement of human blood viscosity by an electromagnetic spinning sphere viscometer. Journal of Medical Engineering and Technology 40(6):285–292.

Gan C, Lankhaar JW, Marcus JT, Westerhof N, Marques KM, Bronzwaer JG, Boonstra A, Postmus PE, Vonk-Noordegraaf A (2006) Impaired left ventricular filling due to right-to-left ventricular interaction in patients with pulmonary arterial hypertension. American Journal of Physiology-Heart and Circulatory Physiology 290:H1528–H1533.

Gardner MD, Scott R (1980) Age- and sex-related reference ranges for eight plasma constituents derived from randomly selected adults in a Scottish new town. Journal of Clinical Pathology 33:380–385.

Gazioglu K, Yu PN (1967) Pulmonary blood volume and pulmonary capillary blood volume in valvular heart disease. Circulation 35:701–709.

Gibson JG, Evans WA (1937) Clinical studies of the blood volume. II. The relation of plasma and total blood volume to venous pressure, blood velocity rate, physical measurements, age and sex in NINETY normal humans. The journal of clinical investigation 16(3):317–328.

Glinicki P, Jeske W, Bednarek-Papierska L, Kruszyńska A, Gietka-Czernel M, Rosłonowska E, Słowińska-Srzednicka J, Kasperlik-Załuska A, Zgliczyński W (2015) The ratios of aldosterone/plasma renin activity (ARR) versus aldosterone/direct renin concentration (ADRR). Journal of the Renin-Angiotensin-Aldosterone System 16(4):1298–1305.

Glossmann H, Baukal AJ, Catt KJ (1974) Properties of angiotensin II receptors in the bovine and rat adrenal cortex. The Journal of Biological Chemistry 249(3):825–834.

Goldberg S, Ollila HM, Lin L, Sharifi H, Rico T, Andlauer O, Aran A, Bloomrosen E, Faraco J, Fang H, Mignot E (2017) Analysis of hypoxic and hypercapnic ventilatory response in healthy volunteers. PLoS One 12(1):e0168930.

Gómez DM (1951) Evaluation of renal resistances, with special reference to changes in essential hypertension. The Journal of Clinical Investigation 30(10):1143–1155.

Guasch A, Cua M, You W, Mitch WE (1997) Sickle cell anemia causes a distinct pattern of glomerular dysfunction. Kidney International 51:826–833.

Guberina H, Baumann M, Bruck H, Feldkamp T, Nürnberger J, Kribben A, Philipp T, Witzke O, Sotiropoulos G, Mitchell A (2013) Associations of smoking with alterations in renal hemodynamics may depend on sex--investigations in potential kidney donors. Kidney Blood Press Res 37:611–621.

Guyton AC, Coleman TG, Granger HJ (1972) Circulation: overall regulation. Annual Review of Physiology 34:13–46.

Hall JE (2011) Guyton and Hall textbook of medical physiology. Twelfth edition. Philadelphia: Saunders Elsevier, 1091 p.

Hallow KM, Lo A, Beh J, Rodrigo M, Ermakov S, Friedman S, de Leon H, Sarkar A, Xiong Y, Sarangapani R, Schmidt H, Webb R, Kondic AG (2014) A model-based approach to investigating the pathophysiological mechanisms of hypertension and response to antihypertensive therapies: extending the Guyton model. American Journal of Physiology. Regulatory, Integrative and Comparative Physiology 306(9):R647–R662.

Hallow KM, Gebremichael Y (2017a) A quantitative systems physiology model of renal function and blood pressure regulation: model description. CPT: Pharmacometrics and Systems Pharmacology 6:383–392.

Hallow KM, Gebremichael Y (2017b) A quantitative systems physiology model of renal function and blood pressure regulation: application in salt-sensitive hypertension. CPT: Pharmacometrics and Systems Pharmacology 6:393–400.

Haluska BA, Jeffriess L, Brown J, Carlier S, Marwick TH (2010) A comparison of methods for assessing total arterial compliance. Journal of Human Hypertension 24:254–256.

Hammer M, Ladefoged J, Olgaard K (1980) Relationship between plasma osmolality and plasma vasopressin in human subjects. American journal of physiology 238:E313–E317.

Hannedouche TP, Delgado AG, Gnionsahe DA, Boitard C, Lacour B, Grünfeld JP (1990) Renal hemodynamics and segmental tubular reabsorption in early type 1 diabetes. Kidney International 37:1126–1133.

Hattori N, Bergsneider M, Wu HM, Glenn TC, Vespa PM, Hovda DA, Phelps ME, Huang SC (2004) Accuracy of a method using short inhalation of (15)O-O(2) for measuring cerebral oxygen extraction fraction with PET in healthy humans. Journal of Nuclear Medicine 45:765–770.

Hayward CS, Kalnins WV, Rogers P, Feneley MP, MacDonald PS, Kelly RP (1997) Effect of inhaled nitric oxide on normal human left ventricular function. Journal of the American College of Cardiology 30(1):49–56.

Henry CJ (2005) Basal metabolic rate studies in humans: measurement and development of new equations. Public Health Nutrition 8(7A):1133–1152.

Hermann K, von Eschenbach CE, von Tschirschnitz M, Ring J (1993) Plasma concentrations of arginine vasopressin, oxytocin and angiotensin in patients with hymenoptera venom anaphylaxis. Regulatory Peptides 49:1–7.

Hill GS, Heudes D, Jacquot C, Gauthier E, Bariéty J (2006) Morphometric evidence for impairment of renal autoregulation in advanced essential hypertension. Kidney International 69:823–831.

Hoang K, Tan JC, Derby G, Blouch KL, Masek M, Ma I, Lemley KV, Myers BD (2003) Determinants of glomerular hypofiltration in aging humans. Kidney International 64:1417–1424.

Hoffer EC, Meador CK, Simpson DC (1969) Correlation of whole-body impedance with total body water volume. Journal of Applied Physiology 27(4):531–534.

Holloway CJ, Montgomery HE, Murray AJ, Cochlin LE, Codreanu I, Hopwood N, Johnson AW, Rider OJ, Levett DZ, Tyler DJ, Francis JM, Neubauer S, Grocott MP, Clarke K (2010) Cardiac response to hypobaric hypoxia: persistent changes in cardiac mass, function, and energy metabolism after a trek to Mt. Everest Base Camp. The FASEB Journal 25:792–796.

Hosten AO (1990) BUN and Creatinine. In: Clinical methods: The history, physical, and laboratory examinations. 3rd edition. Editors: Walker HK, Hall WD, Hurst JW. Boston: Butterworths.

Hoy WE, Hughson MD, Singh GR, Douglas-Denton R, Bertram JF (2006) Reduced nephron number and glomerulomegaly in Australian Aborigines: a group at high risk for renal disease and hypertension. Kidney International 70:104–110.

Hudsmith LE, Petersen SE, Francis JM, Robson MD, Neubauer S (2005) Normal human left and right ventricular and left atrial dimensions using steady state free precession magnetic resonance imaging. Journal of Cardiovascular Magnetic Resonance 7:775–782.

Hughes AD (1998) Molecular and cellular mechanisms of action of angiotensin II (AT1) receptors in vascular smooth muscle. Journal of Human Hypertension 12:275–281.

Hund SJ, Kameneva MV, Antaki JF (2017) A quasi-mechanistic mathematical representation for blood viscosity. Fluids 2(1):10.

Inada Y, Ojima M, Kanagawa R, Misumi Y, Nishikawa K, Naka T (1999) Pharmacologic properties of candesartan cilexetil – possible mechanisms of long-acting antihypertensive action. Journal of Human Hypertension 13(Suppl. 1):S75–S80.

Ishimitsu T, Nishikimi T, Matsuoka H, Kangawa K, Kitamura K, Minami J, Matsuo H, Eto T (1996) Behaviour of adrenomedullin during acute and chronic salt loading in normotensive and hypertensive subjects. Clinical Science 91:293–298.

Jin Y, Kuznetsova T, Maillard M, Richart T, Thijs L, Bochud M, Herregods M-C, Burnier M, Fagard R, Staessen JA (2009) Independent relations of left ventricular structure with the 24-hour urinary excretion of sodium and aldosterone. Hypertension 54:489–495.

Johnson NP, Zelis JM, Tonino PAL, Houthuizen P, Bouwman RA, Brueren GRG, Johnson DT, Koolen JJ, Korsten HHM, Wijnbergen IF, Zimmermann FM, Kirkeeide RL, Pijls NHJ, Gould KL (2018) Pressure gradient vs. flow relationships to characterize the physiology of a severely stenotic aortic valve before and after transcatheter valve implantation. European Heart Journal 39(28):2646–2655.

Juretzko A, Steinbach A, Hannemann A, Endlich K, Endlich N, Friedrich N, Lendeckel U, Stracke S, Rettig R (2017) Urinary angiotensinogen and renin excretion are associated with chronic kidney disease. Kidney and Blood Pressure Research 42:145–155.

Karaaslan F, Denizhan Y, Kayserilioglu A, Ozcan Gulcur H (2005) Long-term mathematical model involving renal sympathetic nerve activity, arterial pressure, and sodium excretion. Annals of Biomedical Engineering 33(11):1607–1630.

Karaaslan F, Denizhan Y, Hester R (2014) A mathematical model of long-term renal sympathetic nerve activity inhibition during an increase in sodium intake. American Journal of Physiology-Regulatory Integrative and Comparative Physiology 306:R234–R247.

Kasner M, Westermann D, Steendijk P, Dröse S, Poller W, Schultheiss HP, Tschöpe C (2012) Left ventricular dysfunction induced by nonsevere idiopathic pulmonary arterial hypertension: a pressure-volume relationship study. American Journal of Respiratory and Critical Care Medicine 186(2):181–189.

Kasner M, Westermann D, Steendijk P, Gaub R, Wilkenshoff U, Weitmann K, Hoffmann W, Poller W, Schultheiss H-P, Pauschinger M, Tschöpe C (2007) Utility of Doppler echocardiography and tissue Doppler imaging in the estimation of diastolic function in heart failure with normal ejection fraction: a comparative Doppler-conductance catheterization study. Circulation 116(6):637–647.

Kass DA, Midei M, Graves W, Brinker JA, Maughan WL (1988) Use of a conductance (volume) catheter and transient inferior vena caval occlusion for rapid determination of pressure-volume relationships in man. Catheterization and Cardiovascular Diagnosis 15:192–202.

Kato M, Kinugawa T, Omodani H, Osaki S, Ogino K, Hisatome I, Miyakoda H, Fujimoto Y (1996) Augmented response in plasma atrial natriuretic peptide to dynamic exercise in patients with congestive heart failure. Japanese Circulation Journal 60:909–916.

Kawaji K, Codella NCF, Prince MR, Chu CW, Shakoor A, LaBounty TM, Min JK, Swaminathan RV, Devereux RB, Wang Y, Weinsaft JW (2009) Automated segmentation of routine clinical

cardiac magnetic resonance imaging for assessment of left ventricular diastolic dysfunction. Circulation: Cardiovascular Imaging 2:476–484.

Keatinge WR, Coleshaw SR, Easton JC, Cotter F, Mattock MB, Chelliah R (1986) Increased platelet and red cell counts, blood viscosity, and plasma cholesterol levels during heat stress, and mortality from coronary and cerebral thrombosis. The American Journal of Medicine 81:795–800.

Kelly RP, Ting CT, Yang TM, Liu CP, Maughan WL, Chang MS, Kass DA (1992) Effective arterial elastance as index of arterial vascular load in humans. Circulation 86:513–521.

Klingensmith ME, Vemuri C, Fayanju OM, Robertson JO, Samson PP, Sanford DE, editors (2016) The Washington manual of surgery. Seventh edition. St. Louis, Missouri: Wolters Kluwer, 984 p.

Knud H, Olesen MD (1967) Interrelations between total exchangeable sodium, potassium, body water, and serum sodium and potassium concentrations in hyponatremic and normonatremic heart disease. Circulation 35:895–903.

Kofranek J, Rusz J (2010) Restoration of Guyton's diagram for regulation of the circulation as a basis for quantitative physiological model development. Physiological Research 59(6):897–908.

Kojima I, Kojima K, Rasmussen H (1985) Role of calcium fluxes in the sustained phase of angiotensin II-mediated aldosterone secretion from adrenal glomerulosa cells. Journal of Biological Chemistry 260(16):9177–9184.

Kovacs G, Berghold A, Scheidl S, Olschewski H (2009) Pulmonary arterial pressure during rest and exercise in healthy subjects: a systematic review. European Respiratory Journal 34:888–894.

Kroll MH, Hellums JD, McIntire LV, Schafer AI, Moake JL (1996) Platelets and shear stress. Blood 88(5):1525–1541.

Kuiper JJ, Boomsma F, van Buren H, de Man R, Danser AH, van den Meiracker AH (2008) Components of the renin-angiotensin-aldosterone system in plasma and ascites in hepatic cirrhosis. European Journal of Clinical Investigation 38(12):939–944.

Kwan WC, Shavelle DM, Laughrun DR (2019) Pulmonary vascular resistance index: Getting the units right and why it matters. Clin Cardiol 42(3):334–338.

Kyhl K, Ahtarovski KA, Iversen K, Thomsen C, Vejlstrup N, Engstrøm T, Madsen PL (2013) The decrease of cardiac chamber volumes and output during positive-pressure ventilation. American Journal of Physiology-Heart and Circulatory and Physiology 305:H1004–H1009.

Lafayette RA, Malik T, Druzin M, Derby G, Myers BD (1999) The dynamics of glomerular filtration after Caesarean section. Journal of the American Society of Nephrology 10:1561–1565.

Lang RM, Bierig M, Devereux RB, Flachskampf FA, Foster E, Pellikka PA, Picard MH, Roman MJ, Seward J, Shanewise J, Solomon S, Spencer KT, Sutton MStJ, Stewart W (2006) Recommendations for chamber quantification. European Journal of Echocardiography 7:79–108.

Laskey WK, Parker HG, Ferrari VA, Kussmaul WG, Noordergraaf A (1990) Estimation of total systemic arterial compliance in humans. Journal of Applied Physiology 69(1):112–119.

Laskey WK, Kussmaul WG 3rd, Noordergraaf A (2009) Systemic arterial response to exercise in patients with aortic valve stenosis. Circulation 119:996–1004.

Lenz T, Nadansky M, Gossmann J, Oremek G, Geiger H (1998) Exhaustive exercise-induced tissue hypoxia does not change endothelin and big endothelin plasma levels in normal volunteers. American Journal of Hypertension 11:1028–1031.

Letcher RL, Chien S, Pickering TG, Sealey JE, Laragh JH (1981) Direct relationship between blood pressure and blood viscosity in normal and hypertensive subjects. Role of fibrinogen and concentration. The American Journal of Medicine 70:1195–1202.

Levey AS, Becker C, Inker LA (2015) Glomerular filtration rate and albuminuria for detection and staging of acute and chronic kidney disease in adults: a systematic review. JAMA 313(8):837–846.

Levin A, Stevens PE (2013) Summary of KDIGO 2012 CKD Guideline: behind the scenes, need for guidance, and a framework for moving forward. Kidney International 85:49–61.

Levinson GE, Pacifico AD, Frank FM (1996) Studies of cardiopulmonary blood volume. Measurement of total cardiopulmonary blood volume in normal human subjects at rest and during exercise. Circulation 33:347–356.

Ljunggren H (1955) Measurement of total body water with deuterium oxide and antipyrine. Acta Physiologica 33(1):69–82.

Luft FC, Fineberg NS, Sloan RS (1982) Estimating dietary sodium intake in individuals receiving a randomly fluctuating intake. Hypertension 4:805–808.

Macedo R, Prakasa K, Tichnell C, Marcus F, Calkins H, Lima JA, Bluemke DA (2007) Marked lipomatous infiltration of the right ventricle: MRI findings in relation to arrhythmogenic right ventricular dysplasia. American Journal of Roentgenology 188(5):W423–W427.

Maceira AM, Prasad SK, Khan M, Pennell DJ (2006a) Normalized left ventricular systolic and diastolic function by steady state free precession cardiovascular magnetic resonance. Journal of Cardiovascular Magnetic Resonance 8:417–426.

Maceira AM, Prasad SK, Khan M, Pennell DJ (2006b) Reference right ventricular systolic and diastolic function normalized to age, gender and body surface area from steady-state free precession cardiovascular magnetic resonance. European Heart Journal 27:2879–2888.

Magder S (2016) Volume and its relationship to cardiac output and venous return. Critical Care 20:271.

Magness RR, Cox K, Rosenfeld CR, Gant NF (1994) Angiotensin II metabolic clearance rate and pressor responses in nonpregnant and pregnant women. American Journal of Obstetrics and Gynecology 171:668–679.

Malisova O, Athanasatou A, Pepa A, Husemann M, Domnik K, Braun H, Mora-Rodriguez R, Ortega JF, Fernandez-Elias VE, Kapsokefalou M (2016) Water intake and hydration indices in healthy European adults: the European hydration research study (EHRS). Nutrients 8:204.

Marini JJ, Leatherman JW (2005) Pulmonary artery occlusion pressure: measurement, significance, and clinical uses. In: Functional hemodynamic monitoring. Update in intensive care and emergency medicine 42. Editors: Pinsky MR, Payen D. Berlin, Heidelberg: Springer.

Mason JW, Ramseth DJ, Chanter DO, Moon TE, Goodman DB, Mendzelevski B (2007) Electrocardiographic reference ranges derived from 79,743 ambulatory subjects. Journal of electrocardiology 40:228–234.

Mehta S, Liu PP, Fitzgerald FS, Allidina YK, Douglas Bradley T (2000) Effects of continuous positive airway pressure on cardiac volumes in patients with ischemic and dilated cardiomyopathy. Am J Respir Crit Care Med 161:128–134.

Melenovsky V, Al-Hiti H, Kazdova L, Jabor A, Syrovatka P, Malek I, Kettner J, Kautzner J (2009) Transpulmonary B-type natriuretic peptide uptake and cyclic guanosine monophosphate release in heart failure and pulmonary hypertension: the effects of sildenafil. J Am Coll Cardiol 54(7):595–600.

Miller WL, Mullan BP (2014) Understanding the heterogeneity in volume overload and fluid distribution in decompensated heart failure is key to optimal volume management: role for blood volume quantitation. JACC Heart Fail 2(3):298–305.

Moore FD (1946) Determination of total body water and solids with isotopes. Science 104(2694):157–160.

Moore FD (1967) Body composition and its measurement in vivo. British Journal of Surgery 54(13):431–435.

Moran D, Epstein Y, Keren G, Laor A, Sherez J, Shapiro Y (1995) Calculation of mean arterial pressure during exercise as a function of heart rate. Applied human sciences 14(6):293–295.

Murch SD, La Gerche A, Roberts TJ, Prior DL, MacIsaac AI, Burns AT (2015) Abnormal right ventricular relaxation in pulmonary hypertension. Pulmonary Circulation 5(2):370–375.

Nadler SB, Hidalgo JU, Bloch T (1962) Prediction of blood volume in normal human adults. Surgery 51:224–232.

Nagueh SF, Smiseth OA, Dokainish H, Andersen OS, Abudiab MM, Schutt RC, Kumar A, Gude E, Sato K, Harb SC, Klein AL (2018) Mean right atrial pressure for estimation of left ventricular filling pressure in patients with normal left ventricular ejection fraction: invasive and noninvasive validation. Journal of The American Society of Echocardiography 31:799–806.

Natarajan AR, Eisner GM, Armando I, Browning S, Pezzullo JC, Rhee L, Dajani M, Carey RM, Jose PA (2016) The renin-angiotensin and renal dopaminergic systems interact in normotensive humans. Journal of the American Society of Nephrology 27:265–279.

Neal CR, Arkill KP, Bell JS, Betteridge KB, Bates DO, Winlove CP, Salmon AHJ, Harper SJ (2018) Novel hemodynamic structures in the human glomerulus. American Journal of Physiology-Renal Physiology 315(5):F1370–F1384.

Nedogoda SV, Ledyaeva AA, Chumachok EV, Tsoma VV, Mazina G, Salasyuk AS, Barykina IN (2013) Randomized trial of perindopril, enalapril, losartan and telmisartan in overweight or obese patients with hypertension. Clinical Drug Investigation 33(8):553–561.

Nguyen MK, Kurtz I (2003) Are the total exchangeable sodium, total exchangeable potassium and total body water the only determinants of the plasma water sodium concentration? Nephrology Dialysis Transplantation 18:1266–1271.

Nozuki M, Mouri T, Itoi K, Takahashi K, Totsune K, Saito T, Yoshinaga K (1986) Plasma concentrations of atrial natriuretic peptide in various diseases. The Tohoku Journal of Experimental Medicine 148:439–447.

Nussberger J, Brunner D, Keller I, Brunner HR (1992) Measurement of converting enzyme activity by antibody-trapping of generatedangiotensin II. Comparison with two other methods. American journal of hypertension 5:393–398.

Oparil S, Acelajado MC, Bakris GL, Berlowitz DR, Cífková R, Dominiczak AF, Grassi G, Jordan J, Poulter NR, Rodgers A, Whelton PK (2018) Hypertension. Nature Reviews Disease Primers 4:18014.

Opitz CF, Wensel R, Bettmann M, Schaffarczyk R, Linscheid M, Hetzer R, Ewert R (2003) Assessment of the vasodilator response in primary pulmonary hypertension. Comparing prostacyclin and iloprost administered by either infusion or inhalation. European Heart Journal 24:356–365.

Ostchega Y, Porter KS, Hughes J, Dillon CF, Nwankwo T (2011) Resting pulse rate reference data for children, adolescents, and adults: United States, 1999-2008. National Health Statistics Reports 41:1–16.

Ottesen JT, Olufsen MS, Larsen JK (2004) Applied mathematical models in human physiology. Philadelphia: SIAM, 298 p.

Otto CM, Pearlman AS, Kraft CD, Miyake-Hull CY, Burwash IG, Gardner CJ (1992) Physiologic changes with maximal exercise in asymptomatic valvular aortic stenosis assessed by Doppler echocardiography. Journal of the American College of Cardiology 20(5):1160–1167.

Pacifico AD, Digerness S, Kirklin JW (1970) Acute alterations of body composition after open intracardiac operations. Circulation 41:331–341.

Paeme S, Moorhead KT, Chase JG, Lambermont B, Kolh P, D'orio V, Pierard L, Moonen M, Lancellotti P, Dauby PC, Desaive T (2011) Mathematical multi-scale model of the cardiovascular system including mitral valve dynamics. Application to ischemic mitral insufficiency. BioMedical Engineering OnLine 10:86.

Pagani ED, Alousi AA, Grant AM, Older TM, Dziuban SWJr, Allen PD (1988) Changes in myofibrillar content and Mg-ATPase activity in ventricular tissues from patients with heart failure caused by coronary artery disease, cardiomyopathy, or mitral valve insufficiency. Circulation Research 63(2):380–385.

Palmer LG, Schnermann J (2015) Integrated control of Na transport along the nephron. Clinical Journal of the American Society of Nephrology 10(4):676–687.

Papaioannou TG, Protogerou AD, Stergiopulos N, Vardoulis O, Stefanadis C, Safar M, Blacher J (2014) Total arterial compliance estimated by a novel method and all-cause mortality in the elderly: the PROTEGER study. Age 36:1555–1563.

Payne RB, Levell MJ (1968) Redefinition of the normal range for serum sodium. Clinical chemistry 14(2):172–178.

Perschel FH, Schemer R, Seiler L, Reincke M, Deinum J, Maser-Gluth C, Mechelhoff D, Tauber R, Diederich S (2004) Rapid screening test for primary hyperaldosteronism: ratio of plasma aldosterone to renin concentration determined by fully automated chemiluminescence immunoassays. Clinical Chemistry 50(9):1650–1655.

Petty WJ, Miller AA, McCoy TP, Gallagher PE, Tallant EA, Torti FM (2009) Phase I and pharmacokinetic study of angiotensin-(1-7), an endogenous antiangiogenic hormone. Clinical Cancer Research 15(23):7398–7404.

Pfisterer ME, Battler A, Zaret BL (1985) Range of normal values for left and right ventricular ejection fraction at rest and during exercise assessed by radionuclide angiocardiography. European Heart Journal 6:647–655.

Pichler GP, Amouzadeh-Ghadikolai O, Leis A, Skrabal F (2013) A critical analysis of whole body bioimpedance spectroscopy (BIS) for the estimation of body compartments in health and disease. Medical Engineering and Physics 35:616–625.

Porthan K, Viitasalo M, Hiltunen TP, Väänänen H, Dabek J, Suonsyrjä T, Hannila-Handelberg T, Virolainen J, Nieminen MS, Toivonen L, Kontula K, Oikarinen L (2009) Short-term electrophysiological effects of losartan, bisoprolol, amlodipine, and hydrochlorothiazide in hypertensive men. Annals of Medicine 41(1):29–37.

Pralong WF, Hunyady L, Várnai P, Wollheim CB, Spät A (1992) Pyridine nucleotide redox state parallels production of aldosterone in potassium-stimulated adrenal glomerulosa cells. Proceedings of the National Academy of Sciences of the United States of America 89:132–136.

Prentice TC, Siri W, Berlin NI, Hyde GM, Parsons RJ, Joiner EE, Lawrence JH (1952) Studies of total body water with tritium. Journal of Clinical Investigation 31(4):412–418.

Proshin AP, Solodyannikov YuV (2006) Mathematical modeling of blood circulation system and its practical application. Automation and Remote Control 67(2): 329–341.

Pruett WA, Clemmer JS, Hester RL (2016) Validation of an integrative mathematical model of dehydration and rehydration in virtual humans. Physiological Reports 4(22):e13015.

Prys-Roberts C, Meloche R, Foëx P (1971) Studies of anaesthesia in relation to hypertension. I. Cardiovascular responses of treated and untreated patients. British Journal of Anaesthesia 43:122–137.

Raimann JG, Zhu F, Wang J, Thijssen S, Kuhlmann MK, Kotanko P, Levin NW, Kaysen GA (2013) Comparison of fluid volume estimates in chronic hemodialysis patients by bioimpedance, direct isotopic, and dilution methods. Kidney International 85:898–908.

Rastegar A (1990) Serum Potassium. In: Clinical methods: The history, physical, and laboratory examinations. 3rd edition. Editors: Walker HK, Hall WD, Hurst JW. Boston: Butterworths.

Ratwatte S, Anderson J, Strange G, Corrigan C, Collins N, Celermajer DS, Dwyer N, Feenstra J, Keating D, Kotlyar E, Lavender M, Whitford H, Whyte K, Williams T, Wrobel JP, Keogh A, Lau EM, PHSANZ Registry (2020) Pulmonary arterial hypertension with below threshold pulmonary vascular resistance. Eur Respir J 56(1):1901654.

Reid IA (1996) Angiotensin II and baroreflex control of heart rate. Physiology 11(6):270–274.

Reynolds RM, Padfield PL, Seckl JR (2006) Disorders of sodium balance. BMJ 332:702–705.

Ribeiro-Oliveira AJr, Nogueira AI, Pereira RM, Boas WW, Dos Santos RA, Simões e Silva AC (2008) The renin-angiotensin system and diabetes: an update. Vascular Health and Risk Management 4(4):787–803.

Riegger GA, Liebau G, Kochsiek K (1982) Antidiuretic hormone in congestive heart failure. Am J Med 72:49–52.

Rodgers KE, Oliver J, diZerega GS (2006) Phase I/II dose escalation study of angiotensin 1-7 [A(1-7)] administered before and after chemotherapy in patients with newly diagnosed breast cancer. Cancer Chemotherapy and Pharmacology 57:559–568.

Rosalina TT, Bouwman RA, van Sambeek MRHM, van de Vosse FN, Bovendeerd PHM (2019) A mathematical model to investigate the effects of intravenous fluid administration and fluid loss. Journal of Biomechanics 88:4–11.

Roumelioti ME, Glew RH, Khitan ZJ, Rondon-Berrios H, Argyropoulos CP, Malhotra D, Raj DS, Agaba EI, Rohrscheib M, Murata GH, Shapiro JI, Tzamaloukas AH (2018) Fluid balance concepts in medicine: principles and practice. World Journal of Nephrology 7(1):1–28.

Safar ME, Chau NP, Weiss YA, London GM, Milliez PL (1976) Control of cardiac output in essential hypertension. The American Journal of Cardiology 38:332–336.

Safar ME, London GM (1985) Venous system in essential hypertension. Clinical Science 69:497–504.

Saghiv M, Sagiv M (2017) Response of left ventricular volumes and ejection fraction during different modes of exercise in health and CAD patients. International Journal of Clinical Cardiology 1:51–56.

Schmitt F, Martinez F, Brillet G, Nguyen-Khoa T, Brouard R, Sissmann J, Lacour B, Grunfeld JP (1998) Acute renal effects of AT1-receptor blockade after exogenous angiotensin II infusion in healthy subjects. Journal of Cardiovascular Pharmacology 31(2):314–321.

Seidlerová J, Staessen JA, Maillard M, Nawrot T, Zhang H, Bochud M, Kuznetsova T, Richart T, Van Bortel LM, Struijker-Boudier HA, Manunta P, Burnier M, Fagard R, Filipovský J (2006) Association between arterial properties and renal sodium handling in a general population. Hypertension 48:609–615.

Senzaki H, Chen C-H, Kass DA (1996) Single-beat estimation of end-systolic pressure-volume relation in humans. A new method with the potential for noninvasive application. Circulation 94(10):2497–2506.

Shoucri RM (2013) Ejection fraction and ESPVR. A study from a theoretical perspective. International Heart Journal 54:318–327.

Shoucri RM (2015) End-systolic pressure-volume relation, ejection fraction, and heart failure: theoretical aspect and clinical applications. Clinical Medicine Insights: Cardiology 9(S1):111–120.

Simón MA, Díez J, Prieto J (1991) Abnormal sympathetic and renal response to sodium restriction in compensated cirrhosis. Gastroenterology 101:1354–1360.

Skøtt P, Hother-Nielsen O, Bruun NE, Giese J, Nielsen MD, Beck-Nielsen H, Parving HH (1989) Effects of insulin on kidney function and sodium excretion in healthy subjects. Diabetologia 32:694–699.

Skrabal F, Arnot RN, Joplin GF (1973) Equations for the prediction of normal values for exchangeable sodium, exchangeable potassium, extracellular fluid volume, and total body water. British Medical Journal 2:37–38.

Skrabal F (1974) Half-life of plasma renin activity in normal subjects and in malignant hypertension. Wiener klinische Wochenschrift 52:1173–1174.

Škrtić M, Lytvyn Y, Yang GK, Yip P, Lai V, Silverman M, Cherney DZ (2015) Glomerular haemodynamic profile of patients with Type 1 diabetes compared with healthy control subjects. Diabetic medicine 32:972–979.

Smilde TDJ, Damman K, van der Harst P, Navis G, Westenbrink BD, Voors AA, Boomsma F, van Veldhuisen DJ, Hillege HL (2009) Differential associations between renal function and "modifiable" risk factors in patients with chronic heart failure. Clinical Research in Cardiology 98:121–129.

Smith D, Engel B, Diskin AM, Spanel P, Davies SJ (2002) Comparative measurements of total body water in healthy volunteers by online breath deuterium measurement and other near-subject methods. The American Journal of Clinical Nutrition 76:1295–1301.

Sobol BJ, Kessler RH, Rader B, Eichna LW (1959) Cardiac, hemodynamic and renal functions in congestive heart failure during induced peripheral vasodilatation; relationship to Starling's law of the heart in man. Journal of Clinical Investigation 38(3):557–578.

Solodyannikov YuV (1994) Elements of mathematical modeling and identification of blood circulation system. Samara: Samara University, 316 p. (In Russ.)

Sperry BW, Campbell J, Yanavitski M, Kapadia S, Tang WHW, Hanna M (2017) Peripheral venous pressure measurements in patients with acute decompensated heart failure (PVP-HF). Circulation: Heart Failure 10:e004130.

Stefanadis C, Manolis A, Dernellis J, Tsioufis C, Tsiamis E, Gavras I, Gavras H, Toutouzas P (2001) Acute effect of clonidine on left ventricular pressure-volume relation in hypertensive patients with diastolic heart dysfunction. Journal of Human Hypertension 15:635–642.

Stellbrink C, Breithardt OA, Franke A, Sack S, Bakker P, Auricchio A, Pochet T, Salo R, Kramer A, Spinelli J, PATH-CHF Investigators, CPI Guidant Congestive Heart Failure Research Group (2001) Impact of cardiac resynchronization therapy using hemodynamically optimized pacing on left ventricular remodeling in patients with congestive heart failure and ventricular conduction disturbances. J Am Coll Cardiol 38(7):1957–1965.

Suga H, Sagawa K, Shoukas AA (1973) Load independence of the instantaneous pressure-volume ratio of the canine left ventricle and effects of epinephrine and heart rate on the ratio. Circulation Research 32: 314–322.

Surveyor I (1969) Sodium, potassium and water metabolism in myxedema. Postgraduate Medical Journal 45:659–663.

Suzuki M, Utida H, Yamaguchi Y, Yamamoto H, Sakai S, Sakai O (1991) A case with renal tubular damage: differentiation of Na reabsorption function by lithium clearance. Japanese Journal of Medicine 30(4):363–366.

Tarazi RC, Dustan HP, Frohlich ED (1969) Relation of plasma to interstitial fluid volume in essential hypertension. Circulation 40:357–366.

Thenappan T, Prins KW, Pritzker MR, Scandurra J, Volmers K, Weir EK (2016) The critical role of pulmonary arterial compliance in pulmonary hypertension. Annals of the American Thoracic Society 13(2):276–284.

Tkacova R, Hall MJ, Liu PP, Fitzgerald FS, Bradley TD (1997) Left ventricular volume in patients with heart failure and Cheyne-Stokes respiration during sleep. Am J Respir Crit Care Med 156:1549–1555.

Treacher DF, Leach RM (1998) Oxygen transport – 1. Basic principles. BMJ 317:1302–1306.

Trip P, Kind T, van de Veerdonk MC, Marcus JT, de Man FS, Westerhof N, Vonk-Noordegraaf A (2013) Accurate assessment of load-independent right ventricular systolic function in patients with pulmonary hypertension. The Journal of Heart and Lung Transplantation 32:50–55.

Tsuda A, Ishimura E, Uedono H, Ochi A, Nakatani S, Morioka T, Mori K, Uchida J, Emoto M, Nakatani T, Inaba M (2018) Association of Albuminuria With Intraglomerular Hydrostatic Pressure and Insulin Resistance in Subjects With Impaired Fasting Glucose and/or Impaired Glucose Tolerance. Diabetes Care 41(11):2414–2420.

Tu W, Eckert GJ, Pratt JH, Jan Danser AH (2012) Plasma levels of prorenin and renin in blacks and whites: their relative abundance and associations with plasma aldosterone concentration. American Journal of Hypertension 25(9):1030–1034.

Uttamsingh RJ, Leaning MS, Bushman JA, Carson ER, Finkelstein L (1985) Mathematical model of the human renal system. Medical and Biological Engineering and Computing 23:525–535.

Valabhji J, Donovan J, Kyd PA, Schachter M, Elkeles RS (2001) The relationship between active renin concentration and plasma renin activity in Type 1 diabetes. Diabetic medicine 18:451–458.

van der Bel R, Coolen BF, Nederveen AJ, Potters WV, Verberne HJ, Vogt L, Stroes ES, Krediet CT (2016) Magnetic resonance imaging-derived renal oxygenation and perfusion during continuous, steady-state angiotensin-II infusion in healthy humans. Journal of the American Heart Association 5:e003185.

van Straten A, Vliegen HW, Lamb HJ, Roes SD, van der Wall EE, Hazekamp MG, de Roos A (2005) Time course of diastolic and systolic function improvement after pulmonary valve replacement in adult patients with tetralogy of Fallot. Journal of the American College of Cardiology 46(8):1559–1564.

Walser M, Duffy BJJr, Griffith HW (1956) Body fluids in hypertension and mild heart failure. Journal of the American Medical Association 160(10):858–864.

Wambach G, Götz S, Suckau G, Bönner G, Kaufmann W (1987) Plasma levels of atrial natriuretic peptide are raised in essential hypertension during low and high sodium intake. Wiener klinische Wochenschrift 65:232–237.

Wang H, Weng C, Chen H (2017) Positive association between KCNJ5 rs2604204 (A/C) polymorphism and plasma aldosterone levels, but also plasma renin and angiotensin I and II levels, in newly diagnosed hypertensive Chinese: a case-control study. Journal of Human Hypertension 31(7):457–461.

Wei CM, Heublein DM, Perrella MA, Lerman A, Rodeheffer RJ, McGregor CG, Edwards WD, Schaff HV, Burnett JrJC (1993) Natriuretic peptide system in human heart failure. Circulation 88:1004–1009.

Wennesland R, Brown E, Hopper JJr, Hodges JLJr, Guttentag OE, Scott KG, Tucker IN, Bradley B (1959) Red cell, plasma and blood volume in healthy men measured by radiochromium (Cr51) cell tagging and hematocrit: influence of age, somatotype and habits of physical activity on the variance after regression of volumes to height and weight combined. Journal of Clinical Investigation 38(7):1065–1077.

Widya RL, van der Meer RW, Smit JW, Rijzewijk LJ, Diamant M, Bax JJ, de Roos A, Lamb HJ (2013) Right ventricular involvement in diabetic cardiomyopathy. Diabetes Care 36:457–462.

Willassen Y, Ofstad J (1980) Renal sodium excretion and the peritubular capillary physical factors in essential hypertension. Hypertension 2(6):771–779.

Wong F, Massie D, Hsu P, Dudley F (1994) Renal response to a saline load in well-compensated alcoholic cirrhosis. Hepatology 20:873–881.

Wright SP, Moayedi Y, Foroutan F, Agarwal S, Paradero G, Alba AC, Baumwol J, Mak S (2017) Diastolic pressure difference to classify pulmonary hypertension in the assessment of heart transplant candidates. Circ Heart Fail 10:e004077.

Wright SP, Opotowsky AR, Buchan TA, Esfandiari S, Granton JT, Goodman JM, Mak S (2018) Flow-related right ventricular to pulmonary arterial pressure gradients during exercise. Cardiovascular Research 115(1):222–229.

Yarmohammadi H, Erinjeri JP, Brown KT (2015) Embolization of metastatic neuroendocrine tumor resulting in clinical manifestations of syndrome of inappropriate secretion of antidiuretic hormone. Journal of Vascular and Interventional Radiology 26:533–537.

Zhang W, Kovács SJ (2008) The diastatic pressure-volume relationship is not the same as the end-diastolic pressure-volume relationship. American Journal of Physiology-Heart and Circulatory Physiology 294: H2750–H2760.

Zhang J, Chen J, Cheong B, Pednekar A, Muthupillai R (2019) High frame rate cardiac cine MRI for the evaluation of diastolic function and its direct correlation with echocardiography. Journal of Magnetic Resonance Imaging 50(5):1571–1582.

Ziegler MG (2018) Atherosclerosis and blood pressure variability. Hypertension 71:403–405.

Zullo MA (2002) Characteristics of the acute rise of atrial natriuretic factor during ventricular pacing. CHEST 121:1942–1946.
